# Controlling Aggregation‐Induced Emission by Sterics in Nitrogen–Boron–Nitrogen (NBN)‐Doped Tetraphenylethylene‐Like Molecular Propellers

**DOI:** 10.1002/chem.202502551

**Published:** 2026-01-29

**Authors:** Nico Groß, Bojan Tanović, Frank Hampel, Henry Dube, Ani Ozcelik

**Affiliations:** ^1^ Department of Chemistry and Pharmacy Friedrich‐Alexander‐Universität Erlangen‐Nürnberg Erlangen Germany

**Keywords:** aggregation‐induced emission, aggregation‐induced quenching, maximum behavior, molecular propellers, nitrogen‐boron‐nitrogen‐doped tetraphenylethylene

## Abstract

The phenomenon of aggregation‐induced emission (AIE) serves as a promising tool to harness and boost emission properties in luminescent materials. Here, we report nitrogen–boron–nitrogen (NBN)‐substituted molecular propellers with different degrees of steric hindrance. Photoluminescence measurements demonstrated that each compound presents AIE behavior in THF/water mixtures, where the less sterically hindered derivative was characterized by higher emission enhancement due to a more pronounced restriction of intramolecular rotations (RIR). A less common maximum behavior was observed upon further addition of water, and this became more pronounced for the more hindered derivative driven by stronger intermolecular electronic effects. Steric hindrance thus serves as an effective design tool to enhance maximum AIE behavior in molecular propellers and gain more precise solvent control.

## Graphical abstract

Nitrogen–boron–nitrogen (NBN)‐substituted molecular propellers are presented as structural framework for aggregation induced emission (AIE) applications. Steric hindrance is revealed to steer maximum‐AIE behavior, leading to refined control over assembly‐induced luminescence.

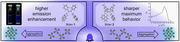

## 1 Introduction

Luminescent materials have garnered considerable attention owing to their promising applications in materials science, optoelectronics, and biosensing, among others. Traditional organic luminophores typically present intense light emission in dilute solution as discrete molecules, yet in aggregate and solid states their photoluminescence is oftentimes diminished or even entirely quenched because of their planar nature and strong aromatic stacking interactions. This effect is known as aggregation‐caused quenching (ACQ), and it is undesirable from the point of many practical applications, e.g., in organic light‐emitting diode (OLED) technologies [1, 2, 3, 4]. On the other hand, aggregation of rotatable molecular platforms possessing deliberate degrees of freedom for intramolecular motions can be used to reverse this behavior in their favor. In such cases aggregation leads to enhanced emission properties, as deexcitation via intramolecular motions is inhibited in the restricted aggregated state. Here, both the restriction of intramolecular rotations (RIR) and the restriction of intramolecular vibrations (RIV) are important sub‐mechanisms for restriction of intramolecular motions (RIM). The dominance of one mechanism over the other strongly depends on the particular molecular structure, and the resulting luminescent behavior is called aggregation‐induced emission (AIE) [3, 5, 6]. After Tang et al. delineated the concept in 2001 for hexaphenysilole (HPS) [7], to date numerous AIE luminogens, or AIEgens, have been reported. Typically, their molecular design relies on fusing phenyl rotor moieties with chromophore‐based stators (i.e., double bonds or phenyl rings) in a twisted geometry. Such molecular design unlocks the radiative decay pathway of electronic excitation when confining phenyl rotor movements in aggregates or solid states. Tetraphenylethylene (TPE) [8], for instance, represents a common structural motif in AIE studies, and its AIE behavior stems from synergistic effects of twisted conformation and RIR similar to HPS [7] or other propeller‐shaped AIEgens [2, 5, 9]. In most instances, an organic solvent is used to fully dissolve AIE molecules, resulting in unhindered motions and low fluorescence output. Upon adding water, aggregation is induced and commonly increasing the water content renders stronger AIE effects [5, 10, 11]. However, there are other examples where the fluorescence intensity diminishes again after exceeding a certain water content [12, 13, 14, 15, 16, 17]. The resulting *maximum AIE effect* offers the possibility to increase solvent control and thus tune emission effects more precisely by the intrinsic polarity of the environment.

Besides the utility of organoboron compounds in synthetic chemistry, boron‐containing π‐systems are well‐established molecular platforms for tailoring optoelectronic properties. Due to the vacant *p*‐orbital and related electron‐accepting ability together with Lewis acidity, boron‐containing π‐systems have been widely employed as emissive organic materials and colorimetric sensors over the past two decades. While there are different strategies to further increase the stability of these boron‐doped molecules, replacing a carbon atom in a C−B bond by a nitrogen heteroatom is especially attractive. Compared to its isoelectronic and isosteric C═C congener, both electron‐acceptor boron and electron‐donating nitrogen in polarizable NB functionality can govern the nature of its double bond and aromatic character [18, 19, 20, 21, 22]. Particularly, the NBN‐doped 1,8‐diaminonaphtyl boronamide (Bdan) family serves as a robust boron‐masking agent and performs well in terms of chemoselectivity in Suzuki−Miyaura coupling reactions [23, 24, 25]. Following the pioneering studies by Suginome and colleagues [23, 24] more recently their potential as fluorophores have been reviewed in diazaboryl‐naphthyl‐ketones [26], pyrene‐ [19], octyl‐ and cyclohexyl‐ [15] or phenol‐functionalized [27] Bdan derivatives. The peripheral editing in some of these and other Bdan families not only allowed for AIE behavior, but also they acted as chemical and colorimetric sensor platforms toward explosives, anions, or persistent pollutants [15, 18, 19, 26, 27].

In this work, we report the synthesis and AIE properties of NBN‐doped molecular propellers as hybrid TPE and Bdan systems. Their structures and photophysical properties are studied by X‐ray diffraction, UV/Vis and fluorescence spectra, and quantum yield measurements, respectively. By combining these experimental results with dynamic light scattering (DLS) and theoretical calculations, we showcase how steric hindrance can control maximum AIE behavior.

## 2 Results and Discussions

**FIGURE 1:**
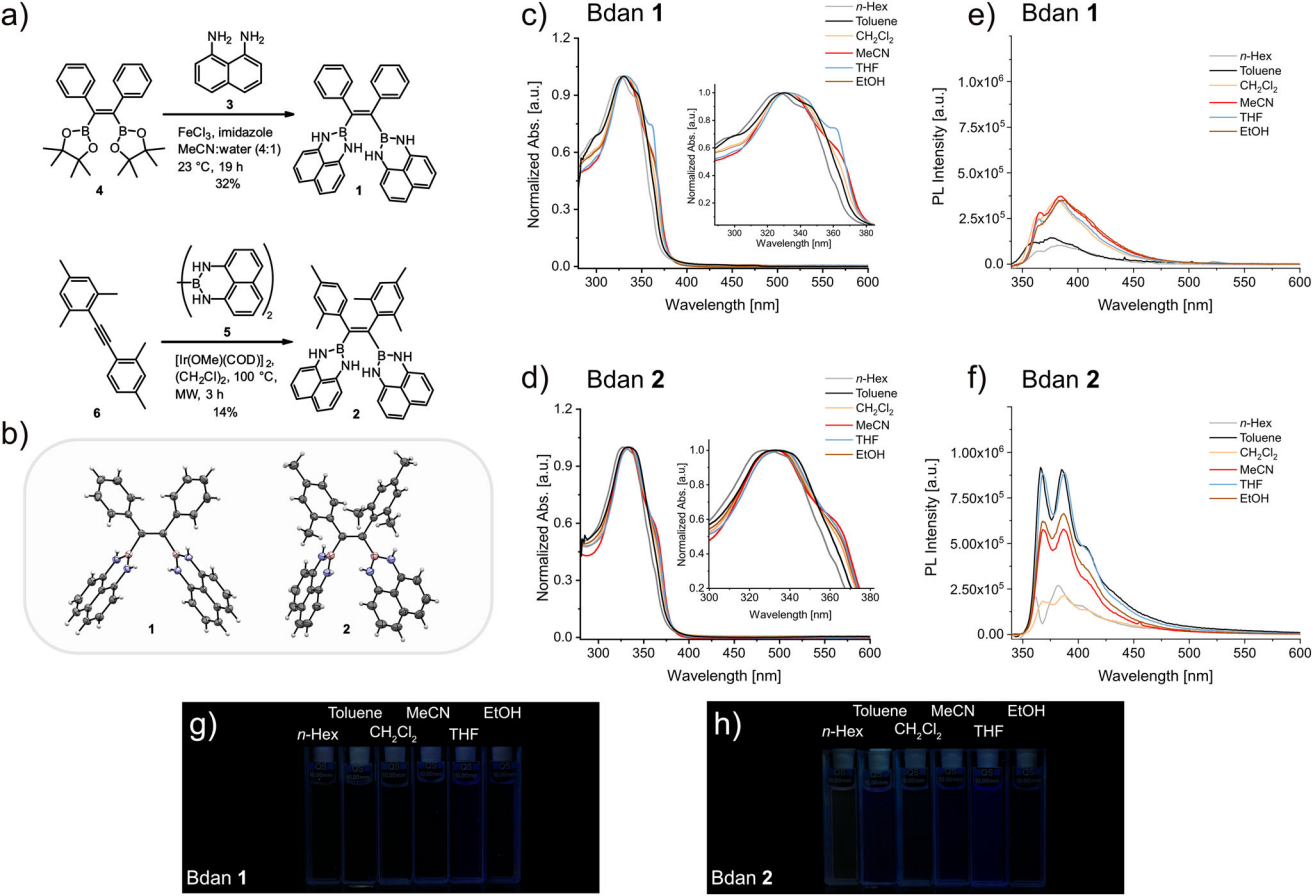
Synthesis and photophysical investigation of Bdans **1** and **2**. (a) Synthesis of Bdans **1** and **2** from precursors **3–6**. (b) Structures of Bdan **1** and **2** in the crystalline state with ellipsoids drawn at the 50% probability level. Hydrogen atoms colored white, carbon atoms grey, boron atoms pink, and nitrogen atoms lilac. UV/Vis absorption and fluorescence spectra of (c), (e) Bdan **1** and (d), (f) Bdan **2,** respectively, in different solvents presenting insignificant solvatochromism effects in both absorption and emission. Photographs of emission (g) and (h) in different solvents (*c* = 5 ⋅ 10^−6^ M) were taken under irradiation with a 340 nm LED.

To reveal the influence of steric encumbrance on AIE properties, NBN‐substituted **1** and **2** were equipped with different groups on the rotor phenyl‐blades, and the synthesis of each compound is depicted in Figure 1. The desymmetrization strategy of phenyl rings in Bdan **2** was chosen to reach an optimal balance between synthetic ease and maximizing steric crowding. Whereas Bdan **1** was earlier prepared *via* Na‐promoted reductive 1,2‐diboration of tolane [28], here we undertook a different synthetic approach and used a FeCl_3_‐imidazole‐water catalytic system [29] to transform Bpin **4** to Bdan **1** in 32% yield. More steric demand in Bdan **2**, on the other hand, called for an alternative route, and [Ir(OMe)COD]_2_‐catalyzed diborylation of internal alkyne **5** with B_2_dan_2_ in (CH_2_Cl)_2_ under microwave‐assisted conditions successfully afforded the target compound in 14% yield. Each derivative was fully characterized by common spectroscopic techniques and their double bond configurations were assigned by X‐ray diffraction analysis and/or 1D NOESY experiments (for further details, see the Supporting Information).

With the target Bdans in hand, their photophysical properties were first investigated by UV/Vis spectroscopy (Figures 1c, 1d). The UV/Vis spectra of **1** and **2** in THF show the absorption maxima at 333 nm and 336, respectively. Compared to the archetypal TPE AIEgen (*λ*_max_  =  308 nm in THF) [30], the absorption maxima of these Bdans are red‐shifted by ca. 27 nm and such absorption fingerprint can be attributed to the replacement of electron‐rich phenyl subunits by NBN‐based heterocycles. For compound **1**, a shoulder peak located at 361 nm is also more pronounced than that of **2** in THF, and it is possible to explain this absorption refinement by the symmetry breaking of inherent propeller conformation rather than a charge‐transfer character of the lowest‐energy absorption band. Such effects are known [31, 32, 33] and can be ascribed here to increased conformational dynamics within less hindered **1**, where torsional disorder of the phenyl blades reduces contribution of the propeller conformation with a higher‐order *C*_2_ symmetry and leads to inhomogeneous broadening. Solvatochromism experiments for both Bdans **1** and **2** were conducted in order to probe the contribution of possible charge transfer in the Franck‐Condon region of the excited state. Only small spectral changes were observed, showing again that the excited state is not gaining significant polarity. The spectral shape of compound **1** is almost identical in *n*‐Hex and toluene, where the absorption maximum is the most blue‐shifted in *n*‐Hex and located at 326 nm. More polar solvents like CH_2_Cl_2_ and EtOH result in very similar absorption behavior. On the other hand, the shoulder peak at 361 nm is increased in MeCN and becomes more prominent in THF. In the case of **2**, the absorption spectra in *n*‐Hex and toluene are comparable; only slight differences were distinguished by varying the solvent polarity from CH_2_Cl_2_ to EtOH.

Luminescence in different organic solvents was investigated next for Bdans **1** and **2** (Figures 1e, 1f). Noticeable variation in fluorescence was observed, which correlated qualitatively with the polarity and viscosity of the solvents. When increasing polarity and viscosity, the fluorescence intensities of each compound increased. No strong shifts of emission maxima were observed by changing the solvents. The latter finding corroborates well on the low polarity of the S_1_ excited state minimum.

**FIGURE 2:**
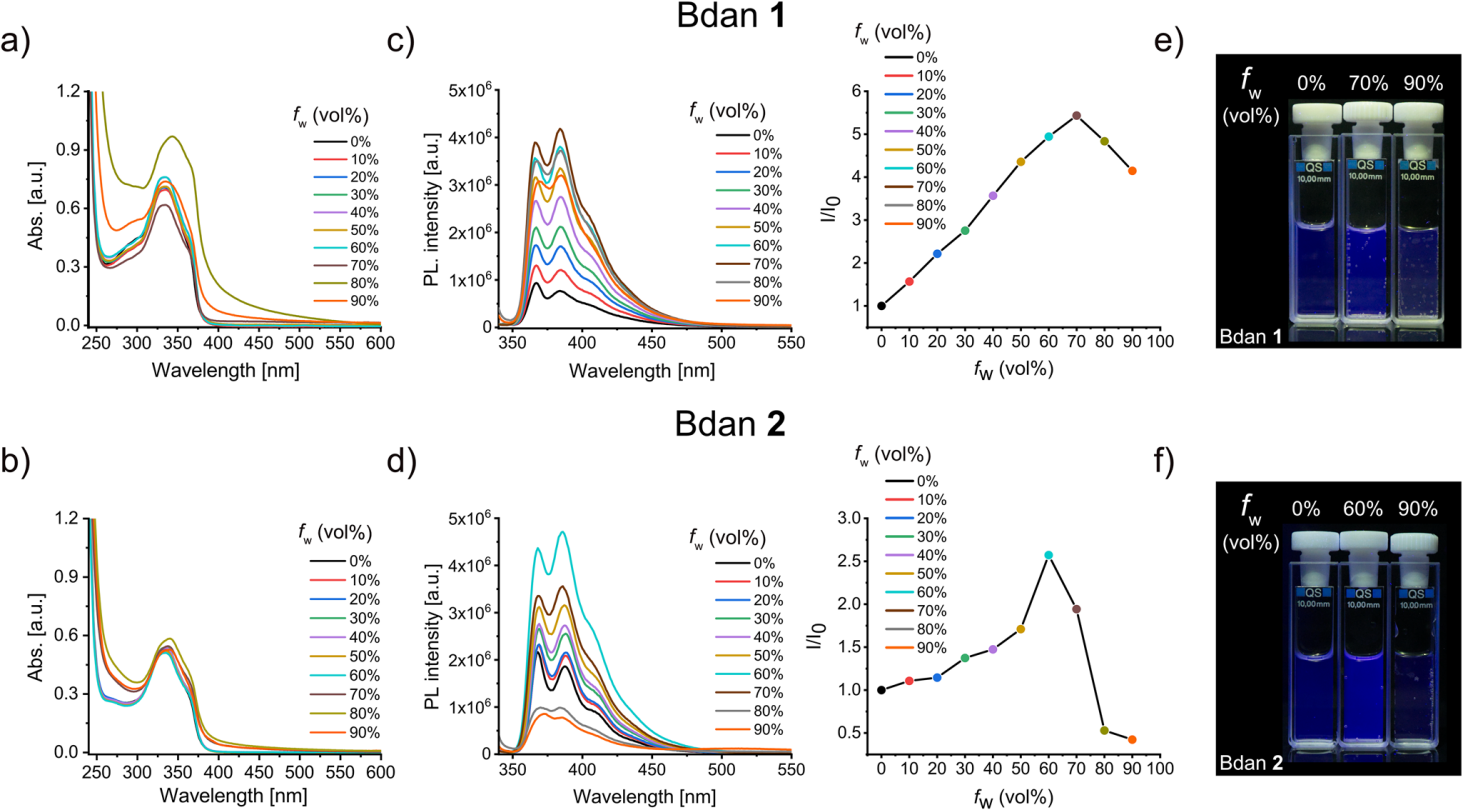
UV/Vis absorption spectra of (a) Bdan **1** and (b) Bdan **2** measured at a concentration of 10^−5^ M in different THF/water mixtures. Fluorescence measurements (left) and plots of relative PL intensity in dependence of water fraction (right) for (c) Bdan **1** and (d) Bdan **2** at a concentration of 10^−6^ M. Distinct maximum behavior for AIE is observed at medium water content of the solvent for Bdans **1** and **2**. The maximum effect is sharper for the sterically hindered derivative Bdan **2**. Photographs of emission for different water fractions of (e) Bdan **1** and (f) Bdan **2** (*c*  =  10^−5^ M) were taken under irradiation with a 340 nm LED.

As compounds **1** and **2** resemble TPE‐Bdan hybrid luminogens and possess very distinct steric crowding at phenyl rotors around a double‐bond stator, their AIE behavior was next addressed by conjunct UV/Vis and fluorescence spectroscopy. Each Bdan derivative is soluble in THF as *good* solvent and they are insoluble in water as *poor* solvent, overall making the THF/water system a suitable environment for driving aggregate formation. Prior to AIE studies, UV/Vis measurements of Bdans **1** and **2** in THF were performed at different concentrations to confirm that aggregation does not take place in this organic solvent alone. Since a linear relationship between the absorbance and concentration was evidenced and no precipitation was visible by the naked eye, these Bdan derivatives were dissolved as monomers in the concentration range under study (Figures S17 and S20 in the Supporting Information). The aggregation effects on the photophysical properties were explored by increasing the water fraction (*f*_w_, in volume percentage) from 0% to 90% in THF and subsequently recording UV/Vis and PL spectra. According to Figure 2, in the absorption spectra of Bdan **1,** the shoulder band at ca. 360 nm becomes somewhat visible upon *f*_w_ = 60%, and further gradual increase in water content, particularly at *f*_w_ = 80% and 90%, leads to a broad band tailing into the visible region of the electromagnetic spectrum with no significant bathochromic shift of the absorption maximum. For Bdan **2**, a similar trend with the formation of a broad absorption band and long‐wavelength tailing was evidenced starting from 70% water in THF. Overall, these results suggest that after certain water content Bdans **1** and **2** form rather undefined aggregates rather than well‐defined morphologies as in J‐ or H‐aggregates [34, 35, 36].

Figures 2c, 2d show the subsequent fluorescence measurements on Bdans **1** and **2** in both THF and THF/water mixtures. The shape of the fluorescence spectra is almost identical for both molecules and emission enhancement was observable upon increasing *f*_w_ until the most emissive state was reached. Further increase in water content led to a drop in the emission response, hinting at ACQ behavior. Considering the spectral shapes, herein reported NBN‐doped molecules feature Bdan‐type fingerprint emission bands in the range of 350−400 nm with no shoulder tailing at longer wavelengths (for example, see refs [15, 22, 26, 27]) and their aggregation‐driven emission is explained as monomer‐like effect due to the absence of a new fluorescence band [37]. Compared to Bdan **1**, the fluorescence spectrum of **2** in pure THF is characterized by a twofold higher emission intensity, hinting at RIM mechanisms that are already partially operative for Bdan **2** in solution due to the high degree of steric hindrance within the molecule. The Bdans also differ in the necessary water content for driving their AIE behavior. A *f*_w_ = 70% was needed for a fourfold increase in fluorescence in the case of Bdan **1**, but *f*_w_  =  60% was sufficient for Bdan **2** to access the most emissive state with twofold enhancement upon comparing to their discrete monomer solutions. Yet, in Bdan **2** ACQ significantly dominates over AIE at *f*_w_ = 90% and establishes the strongest *turn‐off* state with fivefold drop in fluorescence intensity as compared to the most emissive state (*f*_w_ = 60%). The distinct emission behaviors of Bdans **1** and **2** were further quantified by fluorescence quantum yield ($\phi_{f}$) measurements in different solvents. These include pure THF, the particular THF/water mixture (*f*_w_) producing the most emissive state of Bdans **1** and **2**, and the mixture at which fluorescence is quenched again at *f*_w_ = 90%. While recorded quantum yields were moderate in all cases, they are in line with the fluorescence measurements described above. Increasing *f*_w_ from 0% to 70% for Bdan **1** gave rise to an enhancement of $\phi_{f}$ from 0.7% to 2.6% while $\phi_{f}$ dropped slightly to 2.3% for *f*_w_ = 90%. This small change in fluorescence is in agreement with the observed fluorescence intensity changes and the corresponding visual appearance of the samples. As mentioned earlier, Bdan **2** exhibited higher fluorescence efficiency in THF solution ($\phi_{f}$ = 2.6%) compared to Bdan **1**, and its fluorescence quantum yield only doubled in the most emissive state ($\phi_{f}$ = 5.5% at *f*_w_ = 60%) before fluorescence was efficiently quenched for f_w_ = 90% ($\phi_{f}$ = 1.3%). When comparing the *onset* behavior, a sharper and thus better‐defined maximum behavior for the AIE effect in response to *f*_w_ is evidenced for Bdan **2**. The drastic difference of fluorescence efficiency of Bdan **2** between *f*_w_ = 60% and 90% can be accounted for by significantly different aggregation trends at these water fractions. DLS measurements at *f*_w_ = 60% (affording the most emissive state) disclosed a second distinct population of small assemblies (up to 2.5 nm) in addition to aggregates of similar size as evidenced for Bdan **1**. Since increasing the water fraction to 90% leads to the vanishing of the small aggregate fractions and is accompanied by fluorescence quenching, the sharp maximum behavior observed for Bdan **2** at *f*_w_  =  60% is likely caused by the interplay of AIE and ACQ mechanisms. The presence of smaller assemblies in the most emissive state hints at looser confinement where AIE mechanisms are operative to further rigidify the structure. However, intermolecular interactions dominate in the tightly packed aggregated state and drive ACQ for higher *f*_w_.

**FIGURE 3:**
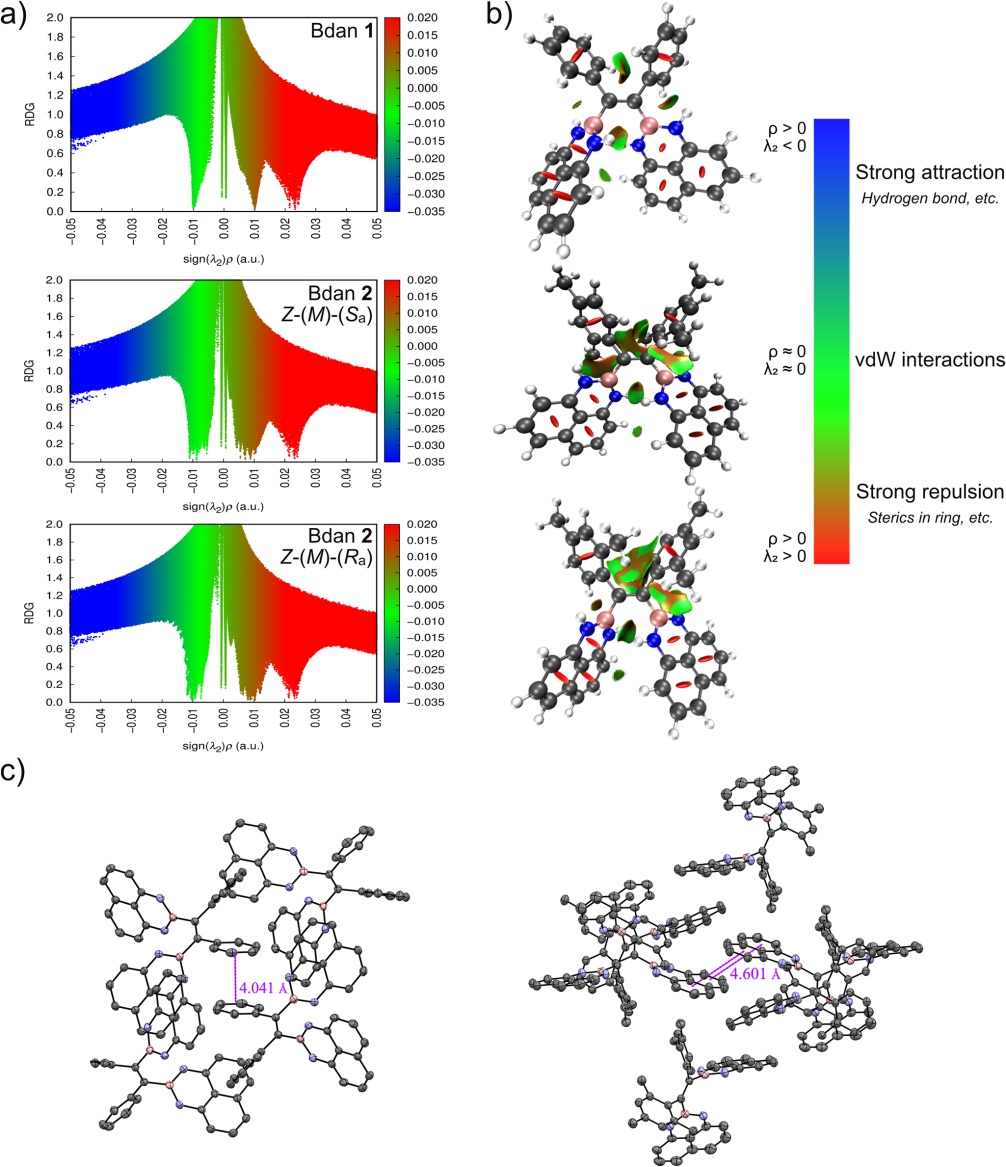
Experimental and theoretical investigations of intra‐ and supramolecular interactions of Bdan **1** and Bdan **2**. (a) Reduced density gradient and (b) non‐covalent interaction plots (M06‐2X/Def2TZVP level of theory) of Bdan **1** (top) and *Z*‐(*M*)‐(*S*a) (middle) and *Z*‐(*M*)‐(*R*a) (bottom) isomers of Bdan **2** monomers. In addition to strong repulsion of rings (red spikes and spheres), the presence of attractive vdW dispersion is evidenced by green spikes and colored disks. Methyl‐substitution in Bdan **2** results in stronger vdW interactions (extended green spikes and regions) and partial steric effects (extended orange spikes and regions), as shown for two possible isomers *Z*‐(*M*)‐(*S*a)‐**2** and *Z*‐(*M*)‐(*R*a)‐**2**. The absence of blue spikes, as well as disks, indicates that no strong intramolecular attractions like hydrogen bonding exist in these Bdan derivatives. The related color coding for each interaction is given in the color scale bar [42]. (c) Packing modes in the crystalline state for Bdan **1** (left) and Bdan **2** (right). Hydrogen atoms were omitted for clarity, and distances of aromatic residues in stacked arrangement are highlighted.

The significant differences between the emission properties of the Bdans can be justified by the conformational space and molecular packing modes because of their inherent molecular structures: in the aggregate state locking rotational freedom about phenyl blades affords a stronger AIE effect overall in Bdan **1**, while the related single bond rotations of Bdan **2** are already more restricted within the discrete monomers. The PL spectra measurements in THF under the same experimental conditions further support this hypothesis, and decorating phenyl blades with bulky methyl groups gives rise to twofold higher fluorescence intensity for fully dissolved Bdans. The nature of each molecule and resulting packing modes, on the other hand, clearly affect the prominence of ACQ over AIE behavior, as evidenced by solid‐state fluorescence measurements that revealed non‐emissive behavior for both compounds in the densely‐packed state. According to X‐ray diffraction analysis, in the crystalline state Bdan **1** adopts supramolecular assemblies mainly driven by multiple CH–π interactions between the phenyl and naphthyl moieties and parallel‐displaced aromatic stacking of phenyl units (Figure 3c). While CH–π interactions are known to be efficient in restricting molecular rotation and enabling AIE mechanisms [5], electronic interactions between stacking aromatic moieties open nonradiative channels and are likely responsible for ACQ as observed for Bdan **1** in the solid state (Figure S25 in the Supporting Information). Even though the presence of methyl groups in Bdan **2** enhances fluorescence emission for fully dissolved molecules, they particularly hamper potential CH–π interactions capable of stabilizing a specific aggregation mode that favors emission. In fact, the crystal structure of Bdan **2** features naphthyl moieties in close and stacked confinements (Figure 3c), ultimately leading to efficient ACQ caused by aromatic intermolecular interactions for aggregates of high *f*_w_ and in the densely‐packed arrangement of molecules in the solid state. Additionally, compared to Bdan **1** aromatic interactions in Bdan **2** are amplified since analysis of key structural parameters uncovered a longer C═C distance for the central double bond and a smaller torsion angle for Bdan moiety in compound **2**, resulting in stronger conjugation compared to Bdan **1** (for more details, see Table S3 and S4 in the Supporting Information). Consequently, Bdan **1** resembles more the TPE‐type character, and its AIE effect still persists over ACQ at *f*_w_ = 90%, in sharp contrast to Bdan **2**. Similarly, these NBN‐doped heterocycles present hydrolytic stability and at certain THF/water mixtures aggregation effects and motion restrictions are solely responsible for their most emissive states rather than decomposition or other processes (Figures S30–S33 in the Supporting Information).

Theoretical calculations were performed in order to shed light on the molecular structures, intramolecular interactions, atropisomerization process, as well as the supramolecular aggregation and AIE behavior of Bdans **1** and **2**. Systematic conformational analyses were conducted, combining both semi‐empirical and DFT levels. In the case of compound **1** with the *Z* double bond configuration, two degenerate minima conformers adopting either (*P*)‐ or (*M*)‐helicities were found as the expression of propeller chirality. This means that the phenyl and Bdan rotor blades orthogonally bound to the central double bond are twisted in either a clockwise or counterclockwise direction. A small barrier for helix inversion inhibits the isolation of each enantiomeric form, which is similar to bare TPE (*ΔG* ^‡^ = 6.5 kcal/mol for helix inversion) [38]. For the *Z*‐isomer of Bdan **2**, the desymmetrization of one of the phenyl rings *via* 2,4‐dimethyl substitution adds atropisomerism and diastereotopic groups, but the resulting (*R*a)‐ or (*S*a)‐configured isomers are conformationally unstable, and their separation is again precluded. Based on variable‐temperature ^1^H NMR experiments in CDCl_3_, with the estimated coalescence temperature (*T*_c_) = –45 °C and exchange rate constant ($k_{exch}$) = 350.3 s^–^^1^, the corresponding Gibbs free energy of activation for the atropisomerization process was calculated to be *ΔG* ^‡^  =  10.58 kcal/mol using the modified Eyring equation [39]. The obtained experimental value is comparable with a correlated rotation resembling a *trans* two‐ring‐flip mechanism, in which the mesitylene and Bdan blades flip by passing through the plane containing the double bond. The remaining desymmetrized 2,4‐dimethyl and Bdan rings do not flip and pass through a plane perpendicular to the reference double‐bond plane [40, 41]. The transition state of such *trans* two‐ring‐flip‐like threshold mechanism is characterized by the *ortho* CH group of the 2,4‐dimethyl phenyl blade positioning itself perpendicular to the mesitylene ring, and *ΔG* ^‡^ for the atropisomerization process was computed as 10.70 kcal/mol at the B3LYP/6‐31G(d) level of theory. Screening different DFT functionals also revealed that Z‐(*M*)‐(*S*a)/*Z*‐(*P*)‐(*R*a) is the global minimum, whereas *Z*‐(*M*)‐(*R*a)/*Z*‐(*P*)‐(*S*a) possesses higher energy varying from 1.90 to 2.39 kcal/mol depending on the level of theory (for further details, see the Supporting Information).

The minima structures obtained from the conformational search were further used to illustrate the degree of steric bulk in each monomeric Bdan derivative. Reduced density gradient (RDG) and non‐covalent interaction (NCI) plots were thus generated for the monomers at the M06‐2X/Def2TZVP level of theory [43], since these methods are useful to determine and visualize non‐covalent interactions including hydrogen bonding or other strong forces, van der Waals (vdW), and repulsive interactions [42, 44]. It can be seen from Figures 3a, 3b that intramolecular non‐covalent interactions in both Bdans are governed by weak attractive vdW dispersion interactions and steric effects among the four rotor blades. Repulsion is also localized on each ring, as indicated by red spikes in the colored RDG scatter plot and the related central red spheres in the NCI plots. Bdan **1** is characterized by attractive vdW interactions and partial effects due to the green and orange regions between phenyl blades and NBN heterocycles in the NCI plot, respectively. These properties are much more pronounced for the two minima conformers of Bdan **2**. Here, larger attractive regions of vdW interaction and significantly increased steric effects with darker orange colors are observed between *ortho* Me‐substituted phenyl rings and the NBN‐doped heterocyclic moieties. The orthogonal disposition of the Me group appended to the atropisomeric phenyl blade leads to expanded interaction regions for *Z*‐(*M*)‐(*S*a)‐**2** and *Z*‐(*M*)‐(*R*a)‐**2**. Twofold interaction regions between each phenyl blade and the NBN‐doped heterocycle fragment for *Z*‐(*M*)‐(*S*a)‐**2** are observed. The theoretical results therefore support the steric‐induced stronger twisting and partial steric effects in Bdan **2**, which are likely inhibiting a tighter aggregation as opposed to Bdan **1**.

In conclusion, we present NBN‐doped TPE‐like hybrid molecular propellers **1** and **2** with different degrees of steric hindrance. The twofold Bdan substitution strategy induces about 27 nm redshift compared to the archetypal TPE AIEgen. As TPE‐Bdan hybrid luminogens, different degrees of sterics control their AIE behavior. Both derivatives show a maximum luminosity behavior upon gradual addition of water to their THF solutions driven exclusively by RIM mechanism. This maximum behavior is much better refined in the case of the sterically hindered derivative Bdan **2**, leading to a sharp increase as well as decrease in luminosity at different water/THF mixtures. The advanced behavior observed for Bdan **2** is correlated with a looser aggregated state and stronger intermolecular electronic effects as compared to the sterically less hindered Bdan **1**, overall resulting in higher sensitivity to solvent changes, aggregate growth, and ACQ. This work demonstrates how steric hindrance in AIEgens can be deployed for a refined tuning of luminescence behavior upon aggregation, and it should also be transferable to other AIE systems.

## Author Contributions

A.O. and H.D. designed and coordinated the project. B.T. performed initial synthesis and characterization of precursors, as well as target compounds and established microwave‐assisted conditions for the synthesis of sterically demanding Bdan **2**. B.T. also performed initial absorption, photoluminescence and NMR experiments, and the related data analysis. N.G. conducted the improved synthesis of Bdan **1**, purified and crystallized compounds **1** and **2** for X‐ray diffraction analysis, and further investigated photophysical properties of **1** and **2** in‐depth by conducting absorption, photoluminescence, NMR and DLS measurements. A.O. investigated the atropisomerization of Bdan **2** by experimental and quantum chemical calculations. A.O. and H.D. wrote the manuscript and edited the Supplementary Information. All contributors discussed, edited and improved the present study, the manuscript and the Supplementary Information.

## Conflicts of Interest

The authors declare no conflict of interest.

## Supporting information



Details of experimental procedures, characterization data (NMR, UV/Vis and PL spectra), DLS analysis, computational studies, and additional data are provided in the Supporting Information.

## Data Availability

All relevant data of this article have been included in the Supporting Information. Crystal structure data have been deposited at the CCDC https://www.ccdc.cam.ac.uk.
