# Supplementary material for: Controlling Aggregation‐Induced Emission by Sterics in Nitrogen–Boron–Nitrogen (NBN)‐Doped Tetraphenylethylene‐Like Molecular Propellers
Source: Chemistry. 2026 Jan 29;32(14):e02551. doi: 10.1002/chem.202502551 (PMC13088003; doi:10.1002/chem.202502551)
Supplement: Supplementary file 1 — Details of experimental procedures, characterization data (NMR, UV/Vis and PL spectra), DLS analysis, computational studies, and additional data are provided in the Supporting Information. [file CHEM-32-e02551-s001.pdf]

Supplementary Information

Controlling Aggregation-Induced Emission by Sterics in Nitrogen–Boron–Nitrogen (NBN)-Doped Tetraphenylethylene-Like Molecular Propellers

Nico Groß,+ [a] *Bojan Tanović,* + † [a] *Frank Hampel,*[a] *Henry Dube** [a] *and Ani Ozcelik** [a]

[a] Friedrich-Alexander-Universität Erlangen-Nürnberg, Department of Chemistry and Pharmacy, Nikolaus-Fiebiger-Str. 10, 91058 Erlangen, Germany.

+ Both authors contributed equally

† *Bojan Tanović* passed away between 15th and 17th of October 2024. His contributions as an author are listed in the Author Contributions section.

Table of Contents

1. Materials and Methods 3

2. Synthesis of Compounds 6

Synthesis of Bdans 6

2-ethynyl-1,3,5-trimethylbenzene (10) 7

2-((2,4-dimethylphenyl)ethynyl)-1,3,5-trimethylbenzene (6) 7

(*Z*)-1,2-diphenyl-1,2-bis(4,4,5,5-tetramethyl-1,3,2-dioxaborolan-2-yl)ethane (4) 9

1*H*,1'*H*,3*H*,3'*H*-2,2'-binaphtho[1,8-*de*][1,3,2]diazaborinine (5) 9

(*Z*)-1,2-bis(1*H*-naphtho[1,8-*de*][1,3,2]diazaborinin-2(3*H*)-yl)-1,2-diphenylethene (1) 10

(Z)-2,2'-(1-(2,4-dimethylphenyl)-2-mesitylethene-1,2-diyl)bis(2,3-dihydro-1*H*-naphtho[1,8-*de*][1,3,2]diazaborinine) (2) 14

3. Structures in the Crystalline State 19

Bdan 1 19

Bdan 2 21

4. UV/Vis Spectra 25

5. Fluorescence Measurements 27

5.1. Measurements in Solution 27

5.2. Measurements in Solid State 30

5.3. Fluorescence Quantum Yield Measurements 31

6. Dynamic Light Scattering Measurements 34

7. Stability Tests on Bdans 36

8. Variable-Temperature ^{1}H NMR Measurements 38

9. Theoretical Calculations 41

9.1. General Consideration for Conformational Analysis 41

9.2. Conformational Analysis on Bdan 1 41

9.3. Conformational Analysis on Bdan 2 43

9.4. Non-Covalent Interactions and Reduced Density Gradient Analysis 46

9.5. Theoretical Studies on the Atropisomerization in Bdan 2 48

10. NMR Spectra of Synthesized Compounds 52

11. Cartesian Coordinates of Optimized Structures 55

12. References 71

1. Materials and Methods

General considerations. All reactions involving air- or moisture-sensitive compounds were carried out in dry reaction vessels under an inert atmosphere of nitrogen using anhydrous solvents and standard Schlenk techniques unless otherwise stated. All oxygen- and moisture-sensitive liquids and anhydrous solvents were transferred *via* a syringe.

Reagents and solvents were obtained from Sigma-Aldrich, Acros Organics, BLD Pharmatech, Alfa Aeasar, TCI, ChemPur, VWR and Carl Roth in the qualities puriss., p.a., or purum and used as received. Technical solvents were further distilled on a rotary evaporator (Heidolph Hei-VAP) prior to use for column chromatography. Reaction progress was monitored on Merck SiO_2 60 F-254 aluminum TLC plates. To determine retention factors, R_f values, compound spots were visualized by irradiation with UV light (254 nm or 366 nm).

Microwave-assisted reactions were conducted using a Biotage microwave synthesizer (Biotage Initiator+) in Biotage vials.

Flash column chromatography was performed on SiO_2 60 (Merck, particle size 0.063-0.200 mm; or Macherey-Nagel, particle size 0.04-0.063 mm) and distilled technical solvents were employed.

High performance liquid chromatography (HPLC) was performed on a Shimadzu HPLC system consisting of a LC-20AP solvent delivery module, a CTO-20A column oven, an SPD-M20A photodiode array UV/Vis detector, and a CBM-20A system controller using a preparative SiO_2 column (particle size 5 μm) from Macherey-Nagel and HPLC grade solvents (*n*-Hex and EtOAc) from Sigma-Aldrich, VWR, and Carl Roth.

Atmospheric pressure photoionization (APPI) mass spectra were measured on a MicroTOF II (Bruker) instrument. Signals are reported in *m/z* units and the molecular ion is reported as M^+.

^{1}H and ^{13}C NMR spectra were obtained on a Bruker Avance 300 (^{1}H: 300 MHz, ^{13}C: 75 MHz), Bruker Avance 400 (^{1}H: 400 MHz, ^{13}C: 100 MHz) or Bruker Avance 500 (^{1}H: 500 MHz, ^{13}C: 125 MHz) spectrometer. Deuterated solvents were provided by Sigma-Aldrich and used as received. Chemical shifts δ are given in parts per million [ppm] and referenced to the solvent ($CDCl_3$: δ_H = 7.26 ppm δ_C = 77.16 ppm, CD_2Cl_2: δ_H = 5.32 ppm, δ_C = 53.84 ppm, THF-d_8: δ_H = 3.58 ppm δ_C = 67.57 ppm). Resonance multiplicity is

indicated as s (singlet), d (doublet), t (triplet), q (quartet), sept (septet) and m (multiplet). Chemical shifts are given in parts per million (ppm). Coupling constant values (*J*) are given in Hertz (Hz).

Infrared spectra (IR) were measured on a 660-IR (Varian, ATR mode) spectrometer. Characteristic IR absorptions are reported in cm^{-1} and labelled as strong (s), medium (m) or weak (w).

Emission spectra were obtained by measuring on a FluoroMax-4 spectrofluorometer. The spectra were recorded in quartz cuvettes (1 cm) and spectral grade solvents obtained from Merck and VWR. Emission wavelengths are reported in nm. As excitation wavelength, either 333 nm or 336 nm was chosen.

Dynamic light scattering measurements (DLS) were performed on a Zetasizer Advance Ultra (Malvern Panalytical) equipped with a 633 nm He-Ne laser. Samples were measured at 30 ºC in filtered (0.2 μm) spectral grade solvents using a 1 cm quartz cuvette. For THF/water mixtures with water fraction of 60% and 70%, parameters were chosen according to the literature.[1] Only the parameters of water were considered for samples with 90% water fraction.

UV/Vis spectra were recorded on an Agilent Cary 60 spectrophotometer. The spectra were recorded in quartz cuvettes (1 cm) and spectral grade solvents obtained from Merck and VWR. Absorption wavelengths (λ) are reported in nm.

Melting points (mp) were measured on a Büchi B-540 melting point apparatus in open capillaries.

Quantum chemical calculations were performed with the software package Gaussian 09 Rev. A.02[2] and Gaussian 16 Rev. B.01.[3] Optimized structures were visualized with Gaussview 6.0.16. The geometry optimizations were carried out only in the ground state and all obtained minima, as well as transition states were verified by subsequent frequency calculations. Ground state minima structures possess no imaginary frequency, while transition states are characterized by only one negative frequency corresponding to a first-order saddle point on the potential energy surface (PES). The relative energies of each isomer were obtained with respect to the lowest energy structure. DFT energies were corrected by a thermal free energy correction to give the relative Gibbs free energies (ΔG^0_{rel}) of the respective isomers. Reduced density gradient (RDG) and non-covalent

interaction (NCI) analyses were performed using the Multiwfn software package, version 3.8.[4] Structures and NCI plots were visualized using VMD 1.9.3. Colored RDG plots were generated by Gnuplot 5.4.

X-ray diffraction analysis was performed on a SuperNova Atlas diffractometer using Cu-Kα-radiation for Bdan **1**, and on a XtaLAB Synergy R, HyPix-Arc 100 diffractometer using Cu-Kα-radiation for Bdan **2.**

2. Synthesis of Compounds

Synthesis of Bdans

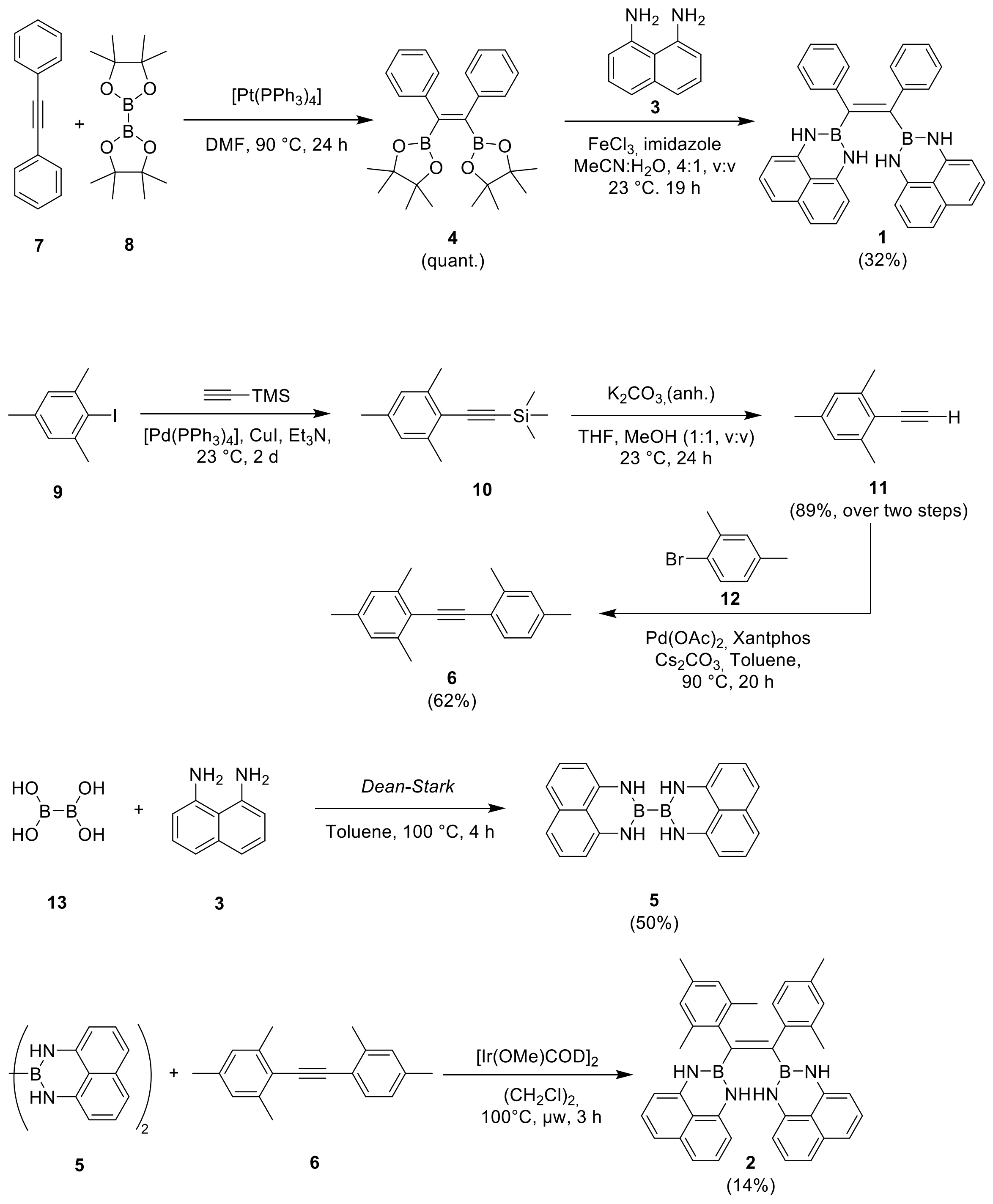

Scheme 1. Synthetic routes for Bdans **1** and **2**.

2-ethynyl-1,3,5-trimethylbenzene (10)

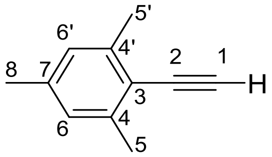

Compound **9** (200 mg, 0.81 mmol, 1.0 equiv.), [Pd(PPh_3)$_4$] (47.0 mg, 0.04 mmol, 0.05 equiv.) and CuI (16.0 mg, 0.08 mmol, 0.1 equiv.) were placed in a Schlenk flask, suspended in Et_3N (3.0 mL) and purged with N_2. The mixture was cooled to 0 °C and trimethylsilylacetylene (0.18 mL, 1.25 mmol, 1.5 equiv.) was added dropwise at this temperature. The mixture was then allowed to warm up to 23 °C and stirred for 48 h. The crude product was filtered through a short pad of SiO_2 (Eluent: CH_2Cl_2). After removing the solvent in *vacuo*, the residue was purified *via* flash column chromatography (SiO_2, *i*-Hex, 100%). The isolated product **10** and anhydrous K_2CO_3 (299 mg, 2.16 mmol, 3.0 equiv.) were re-suspended in a mixture of MeOH (1.9 mL) and THF (1.9 mL). The mixture was stirred for 26 h at 23 °C before water (20 mL) was added. The aqueous phase was extracted with Et_2O (3 x 30 mL), the combined organic layers were washed with brine (2 x 20 mL) and dried over anhydrous Na_2SO_4. After solvent removal, compound **11** (92.3 mg, 0.64 mmol, 89%) was obtained as yellow oil.

***R*f** (SiO_2, *i*-Hex 100%) = 0.54.

^{1}H NMR (400 MHz, $CDCl_3$,) δ / ppm = 6.86 (s, 2H), 3.45 (s, 1H), 2.41 (s, 6H), 2.28 (s, 3H). The spectroscopic data are identical to those reported.[5]

Note: Intermediates **10** and **11** are volatile and should be handled carefully during solvent evaporation.

2-((2,4-dimethylphenyl)ethynyl)-1,3,5-trimethylbenzene (6)

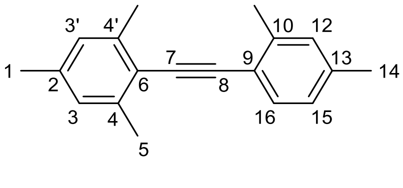

Compound **11** (339 mg, 2.35 mmol, 1.0 equiv.) was dissolved in toluene (4.00 mL) and the solution was transferred to a dry Schlenk flask together with 1-bromo-2,4-dimethylbenzene **12** (0.38 mL, 2.82 mmol, 1.2 equiv.), anhydrous Cs_2CO_3 (6.12 g, 18.8 mmol, 8.0 equiv.), Pd(OAc)$_2$ (52.8 mg, 0.24 mmol, 0.1 equiv.) and Xantphos (272 mg,

0.47 mmol, 0.2 equiv.). After adding toluene (19.5 mL), the resulting suspension was purged with N_2 and stirred for 20 h at 90 °C. Once cooling down to 23 ºC, the mixture was transferred to a separatory funnel charged with a saturated aqueous solution of NH_4Cl and the aqueous layer was extracted with CH_2Cl_2 (3 x 50 mL). The combined organic phases were washed with brine (50 mL) and dried over anhydrous Na_2SO_4. After removing the solvent, the residue was purified by flash column chromatography (SiO_2, *i*-Hex, 100%) and compound **10** (362 mg, 1.46 mmol, 62%) was obtained as off-white solid.

***R*f** (SiO_2, *i*-Hex, 100%) = 0.44.

^{1}H NMR (400 MHz, $CDCl_3$,) *δ* / ppm = 7.41 (d, *J* = 7.8 Hz, 1H, H–C(16)), 7.06 (s, 1H, H–C(12)), 6.99 (d, *J* = 7.8 Hz, 1H, H–C(15)), 6.91 (s, 2H, H–C(3) and H–C(3')), 2.50 (s, 3H, H–C(11)), 2.49 (s, 6H, H_3–C(5) and H_3–C(5')), 2.34 (s, 3H, H_3–C(14)), 2.30 (s, 3H, H_3–C(1)).

^{13}C NMR (100 MHz, $CDCl_3$) *δ* / ppm = 140.0 (C(10)), 139.6 (C(6)), 138.1 (C(4)), 137.6 (C(13)), 131.8 (C(16)), 130.5 (C(12)), 127.8 (C(3)), 126.5 (C(15)), 121.0 (C(9)), 120.6 (C(2)), 96.4 (C(8)), 90.7 (C(7)), 21.6 (C(14)), 21.5 (C(1)), 21.3 (C(11)), 21.1 (C(5)).

HR-MS (APPI), [M^+]: *m/z* calcd.:249.1638 for $[C_{19}H_{21}]^+$, found: 249.1639.

IR: *v* / cm^{-1} = 3004 (w), 2969 (w), 2940 (m), 2911 (w), 2849 (w), 2726 (w), 1609 (m), 1557 (w), 1507 (s), 1495 (m), 1452 (m), 1433 (m), 1373 (w), 1302 (w), 1289 (w), 1158 (w), 1033 (w), 1012 (w), 945 (w), 929 (w), 869 (w), 849 (s), 813 (s), 726 (m), 573 (m), 558 (m), 502 (w).

Melting Point: 63 °C.

(*Z*)-1,2-diphenyl-1,2-bis(4,4,5,5-tetramethyl-1,3,2-dioxaborolan-2-yl)ethane (4)

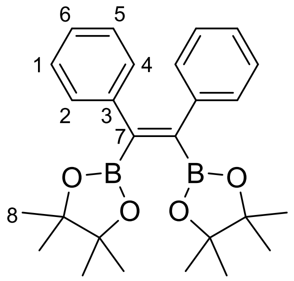

Tolane **7** (500 mg, 2.81 mmol, 1.0 equiv.), B_2pin_2 **8** (785 mg, 3.10 mmol, 1.1 equiv.), and [Pt(PPh_3)$_4$] (99.5 mg, 0.08 mmol, 0.03 equiv.) were submitted to an evacuated 20 mL microwave flask. After the addition of dry DMF (10 mL), the reaction mixture was purged with N_2 for 10 minutes and it was stirred at 90 °C for 24 h. Once cooled down to 23 °C, the crude was filtered through a short pad of Celite (Eluent: EtOAc). Product **9** (1.21 g, 2.81 mmol, quantitative conversion) was obtained as white solid and it was employed in the next step without further purification.

***R*f** (SiO_2, *i*-Hex:EtOAc, 90:10, v:v) = 0.63.

^{1}H NMR (400 MHz, CD_2Cl_2) δ / ppm = 7.10 – 7.03 (m, 6H, ArH), 6.92 – 6.88 (m, 4H, ArH), 1.31 (s, 24H, H–C(8)). The spectroscopic data are identical to those previously reported.[6]

1*H*,1'*H*,3*H*,3'*H*-2,2'-binaphtho[1,8-*de*][1,3,2]diazaborinine (5)

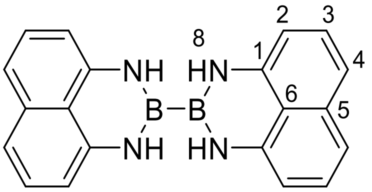

Tetrahydroxydiboron **13** (283 mg, 3.16 mmol, 1.0 equiv.) and diamine **3** (1.00 g, 6.32 mmol, 2.0 equiv.) were placed in a flame-dried Schlenk flask and dissolved in toluene (12 mL). The system was equipped with a Dean-Stark trap and refluxed at 90 °C under N_2 atmosphere. The resulting mixture was allowed to cool to 23 °C and the solvent was removed in *vacuo*. The residue was re-suspended in hot EtOAc (50 mL) and the suspension was filtered off. After removing the solvent, the residue was purified via flash column chromatography (SiO_2, *i*-Hex:EtOAc, 85:15, v:v) to yield compound **8** (530 mg, 1.59 mmol, 50%) as pale pink solid.

***R*f** (SiO_2, *i*-Hex:EtOAc, 85:15, v:v) = 0.26.

^{1}H NMR (400 MHz, $CDCl_3$,) *δ* / ppm = 7.14–7.07 (m, 4H), 7.05–6.99 (m, 4H), 6.32 (d, *J* = 7.1 Hz, 4H), 6.00 (br s, 4H, 4 × N–H). The spectroscopic data are identical to those previously reported.[7]

HR-MS (APPI), [M$^+$]: *m/z* calcd.:335.1634 for $[C_{20}H_{17}B_2N_4]^+$, found: 335.1636.

(*Z*)-1,2-bis(1*H*-naphtho[1,8-*de*][1,3,2]diazaborinin-2(3*H*)-yl)-1,2-diphenylethene (1)

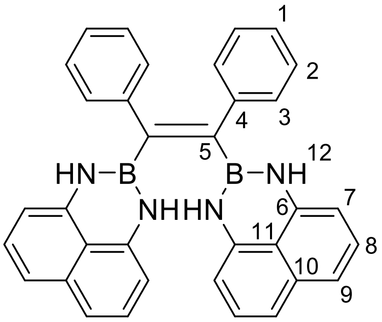

Compound **9** (40.0 mg, 0.93 mmol, 1.0 equiv.), 1,8-diaminonaphthalene (32.3 mg, 0.20 mmol, 2.2 equiv.), $FeCl_3$ (2.50 mg, 0.02 mmol, 0.16 equiv.), and imidazole (25.3 mg, 0.37 mmol, 4.0 equiv.) were suspended in a mixture of MeCN (1.20 mL) and water (0.30 mL). The mixture was left to stir at 23 °C for 19 h. EtOAc (20 mL) was added, and the phases were separated. The aqueous phase was extracted with EtOAc (2 x 20 mL). The combined organic phases were washed with sat. aq. NaCl solution (20 mL) and dried over Na_2SO_4. After solvent removal, the residue was purified by flash column chromatography (SiO_2, *i*-Hex:EtOAc, gradient from 92:8 to 90:10, v:v) to yield compound **1** (15.1 mg, 29.5 mmol, 32%) as pale yellow solid.

Rf (SiO_2, *i*-Hex:EtOAc, 90:10, v:v) = 0.25.

^{1}H NMR (400 MHz, CD_2Cl_2,) *δ* / ppm = 7.20–7.08 (m, 6H, ArH), 7.06–6.96 (m, 12H, ArH), 6.23 (dd, *J* = 7.2, 1.1 Hz, 4H, H–C(7)), 5.96 (br s, 4H, 4 x N–H).

^{13}C NMR (100 MHz, CD_2Cl_2,) *δ* / ppm = 142.7, 141.2, 136.6, 130.0, 128.4, 128.0, 126.6, 120.0, 118.3, 106.4

The spectroscopic data are identical to those reported in the literature.[8]

Single crystals of *Z*-**1** for X-ray diffraction analysis could be obtained by recrystallization from CH_2Cl_2 and *i*-Hex mixture (1:1).

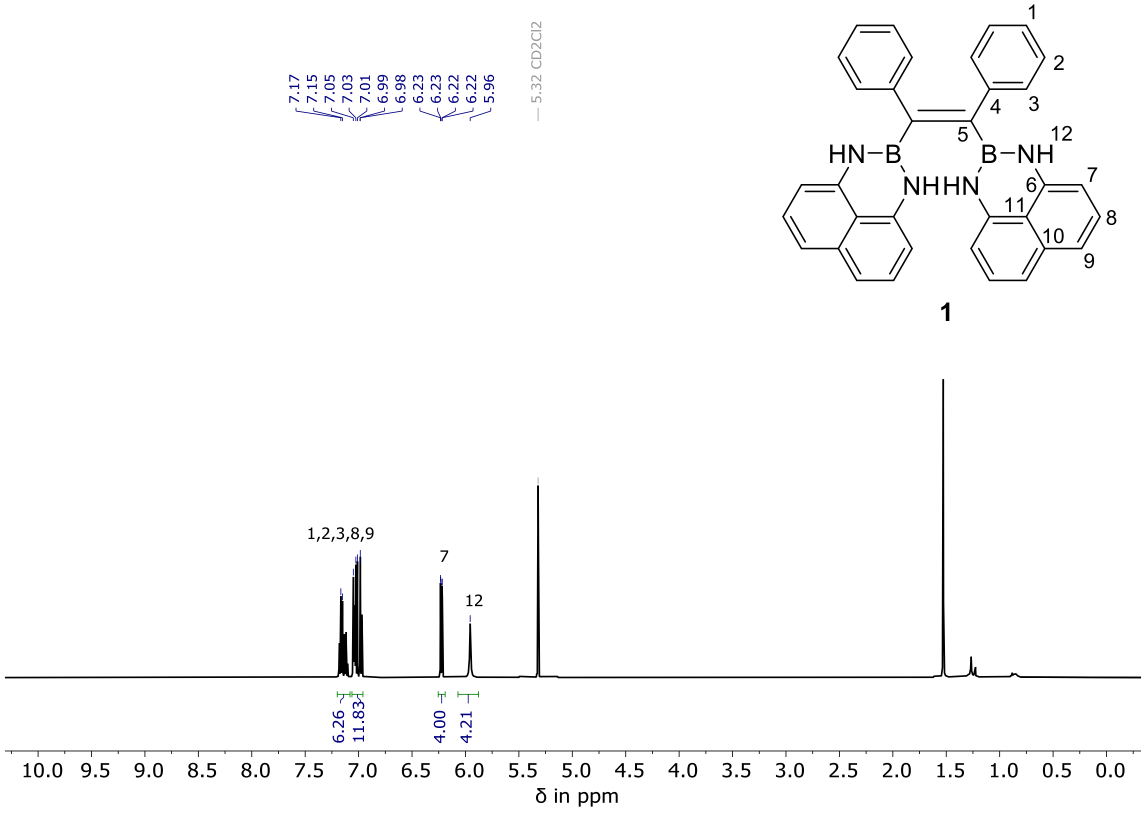

Figure S1. ^{1}H NMR spectrum (500 MHz, CD_2Cl_2, 23 °C) of compound **1**.

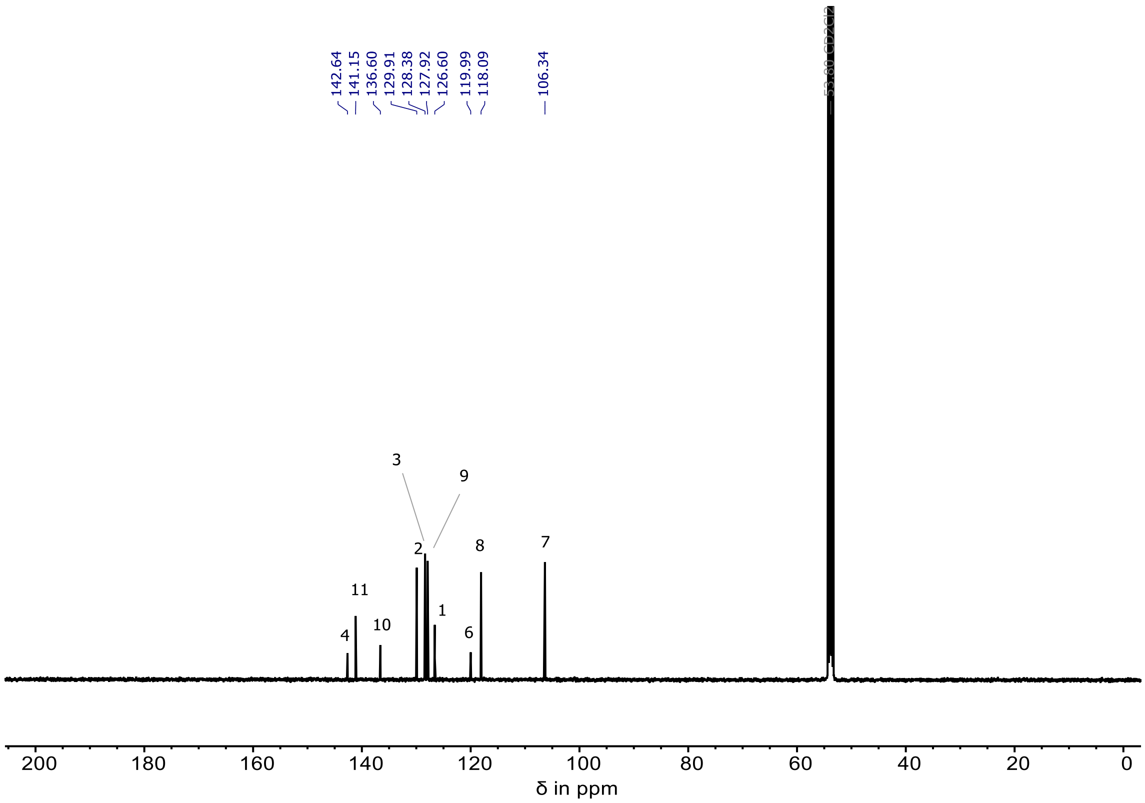

Figure S2. ^{13}C NMR spectrum (126 MHz, CD_2Cl_2, 23 °C) of compound **1**.

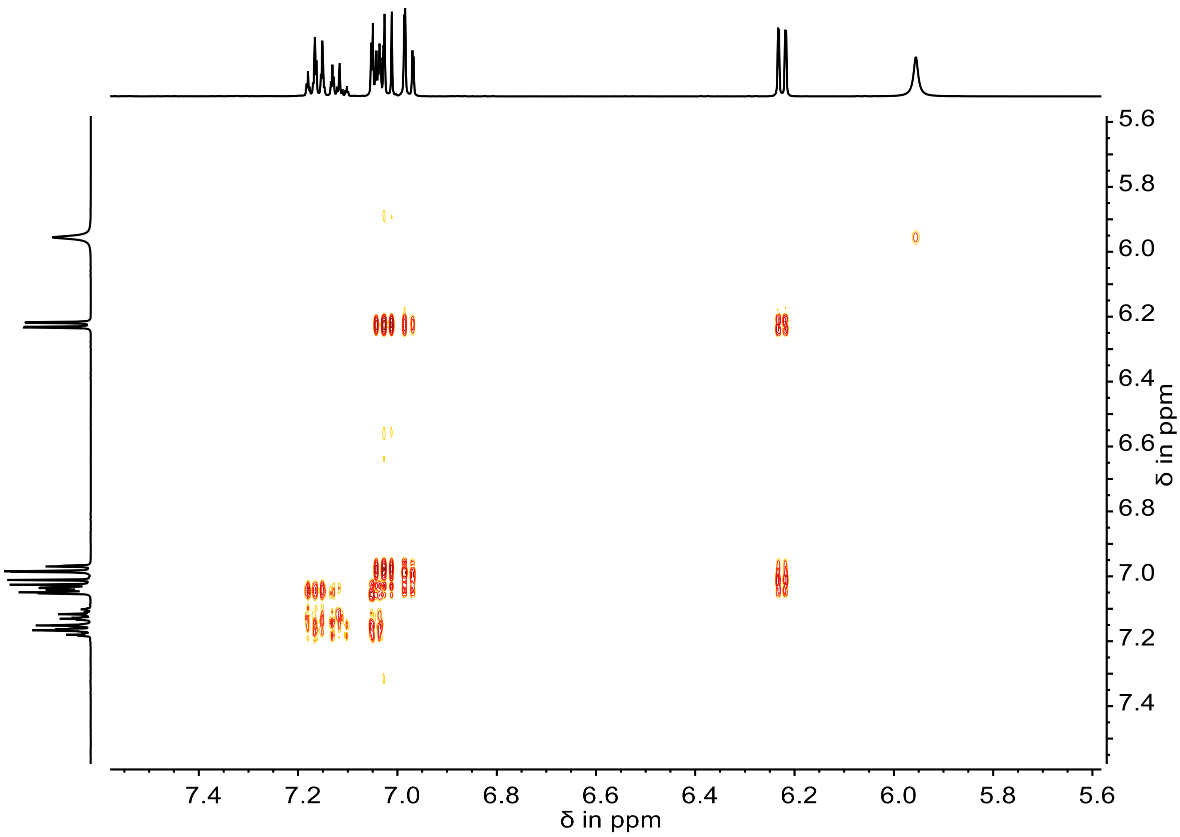

Figure S3. ^{1}H-^{1}H COSY NMR spectrum (500 MHz, CD_2Cl_2, 23 °C) of compound **1**.

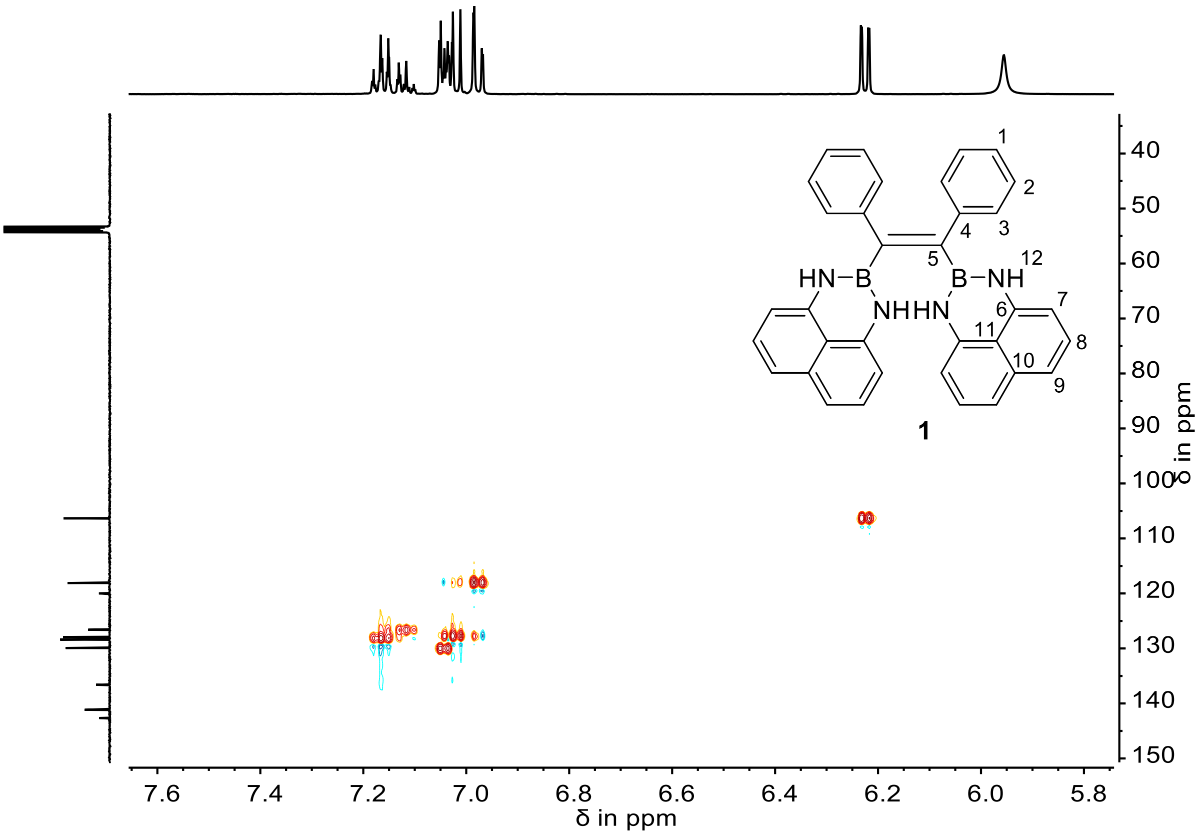

Figure S4. ^{1}H-^{13}C HSQC NMR spectrum (500 MHz, CD_2Cl_2, 23 °C) of compound **1**.

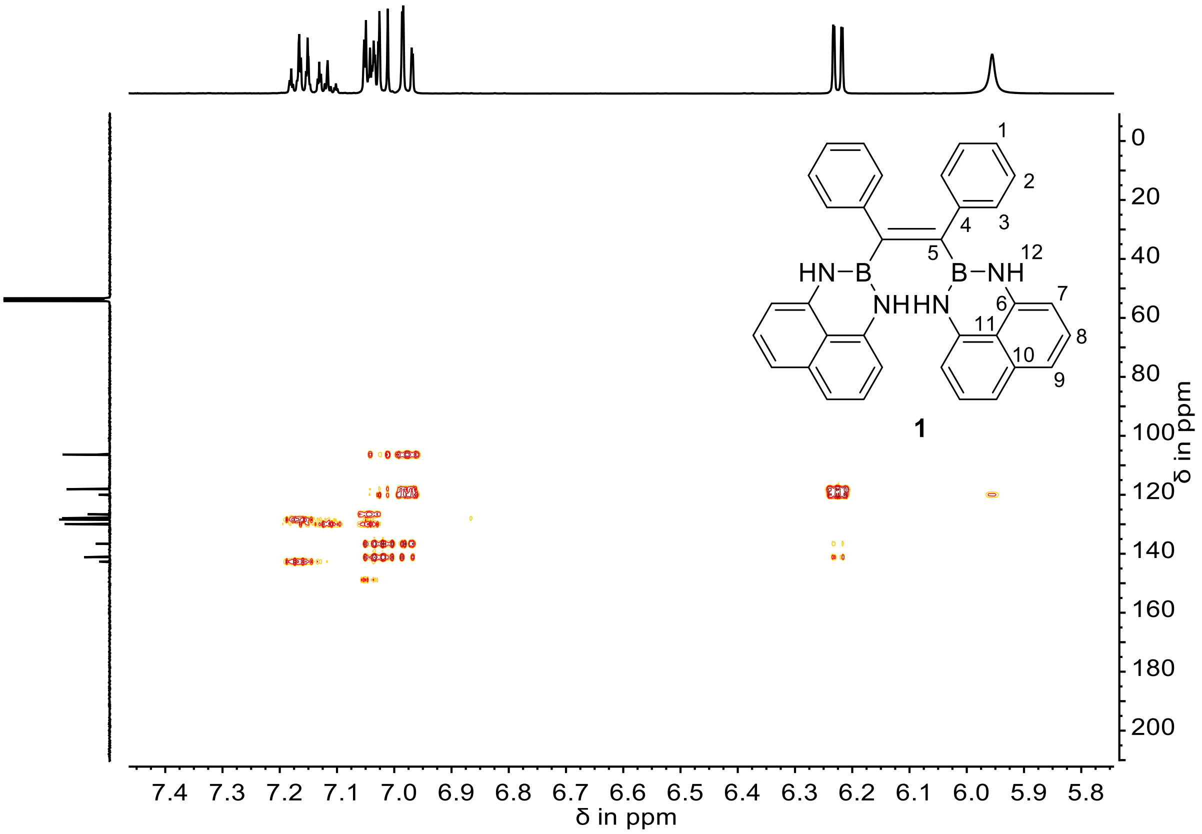

Figure S5. ^{1}H-^{13}C HMBC NMR spectrum (500 MHz, CD_2Cl_2, 23 °C) of compound **1**.

(Z)-2,2'-(1-(2,4-dimethylphenyl)-2-mesitylethene-1,2-diyl)bis(2,3-dihydro-1*H*-naphtho[1,8-*de*][1,3,2]diazaborinine) (2)

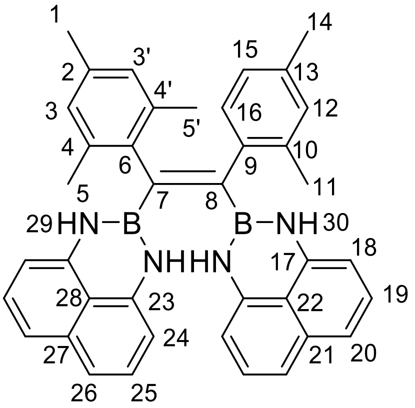

Compound **5** (200 mg, 0.80 mmol, 1.0 equiv.), compound **4** (296 mg, 0.88 mmol, 1.1 equiv.), and $[Ir(OMe)COD]_2$ (16.0 mg, 0.02 mmol, 0.03 equiv.) were suspended in 1,2-dichloroethane (2.40 mL) and heated for 3 h to 100 °C by microwave irradiation under N_2 atmosphere. The crude solution was filtered through a short pad of Celite (eluent: EtOAc), and the solvent was removed. Purification *via* flash column chromatography (SiO_2, *i*-Hex:EtOAc, gradient from 98:2 to 90:10, v:v) followed by HPLC using a preparative Macherey-Nagel Nucleosoil SiO_2 column (*i*-Hex:CH_2Cl_2, 70:30, v:v, flow rate: 15 mL min^{-1}) gave propeller **2** (67.6 mg, 0.12 mmol, 14%) as pale yellow solid.

R_f (SiO_2, *i*-Hex:EtOAc, 90:10, v:v) = 0.36

^{1}H NMR (500 MHz, CD_2Cl_2,) δ / ppm = 7.06–6.92 (m, 8H, H-C(19), H-C(20) H-C(25), H-C(26)), 6.90 (s, 1H, H-C(12)), 6.76 (s, 2H, H-C(3) and H-C(3')), 6.72–6.65 (m, 2H, H-C(15), H-C(16)), 6.23 (dd, J = 7.1, 1.2 Hz, 2H, H-C(18) or H-C(24)), 6.14 (dd, J = 7.2, 1.2 Hz, 2H, H-C(18) or H-C(24)), 5.84 (br s, 2H, 2 x N-H), 5.76 (br s, 2H, 2 x N-H), 2.32 (s, 3H), 2.23–2.17 (m, 12H).

^{13}C NMR (126 MHz, CD_2Cl_2,) δ / ppm = 141.3, 141.2, 136.7, 136.6, 136.0, 135.8, 131.5, 129.1, 128.7, 128.0, 128.0, 126.2, 120.0, 118.1, 117.9, 106.3, 106.2, 30.1, 21.1, 21.0, 20.9. Due to the quadrupole nature of B, C atoms directly bound to it could not be observed.

Note: Due to the structural complexity, stereodynamic nature and overlap of NMR signals of the compound, certain assignment of the proton and carbon atoms could not be achieved. The obtained chemical shifts and integrals are in good agreement with the values expected for such systems.

HR-MS (APPI), [M$^+$]: *m/z* calcd.: 582.3121 for $[C_{39}H_{36}B_2N_4]^+$, found 582.3120.

IR: v / cm^{-1} = 3404 (m), 2951 (w), 2921 (m), 2850 (w), 1722 (w), 1627 (m), 1593 (s), 1556 (w), 1531 (s), 1461 (m), 1403 (s), 1373 (s), 1355 (m), 1327 (m), 1279 (m), 1187 (s), 1165 (m), 1077 (m), 1061 (m), 1034 (w), 851 (m), 818 (s), 795 (s), 719 (s), 699 (m).

Melting Point: decomposition at 243 °C.

Single crystals of *Z*-**2** for X-ray diffraction analysis could be obtained by recrystallization from CH_2Cl_2 and heptane mixture (1:1).

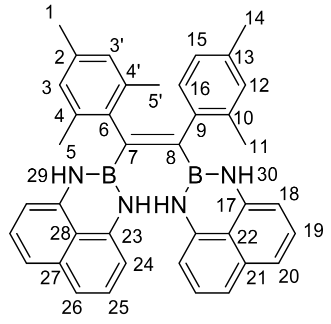

2

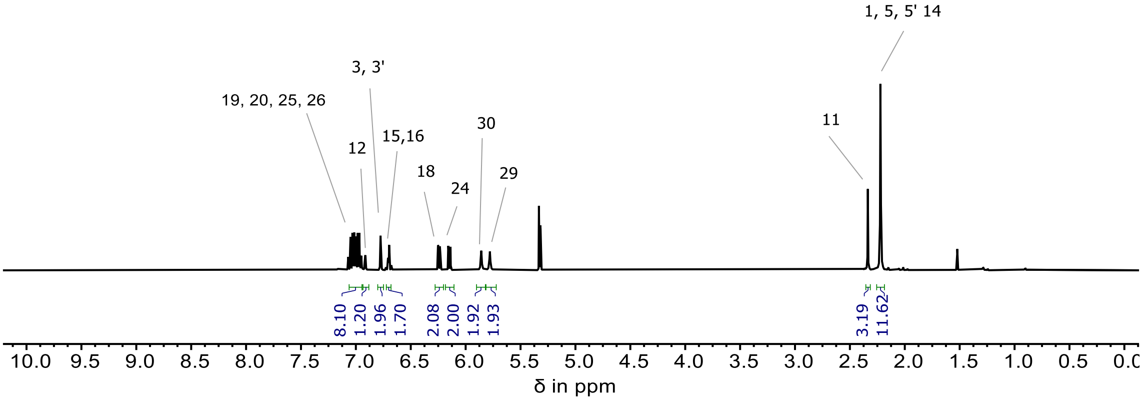

Figure S6. ^{1}H NMR spectrum (400 MHz, CD_2Cl_2, 23 °C) of compound **2**.

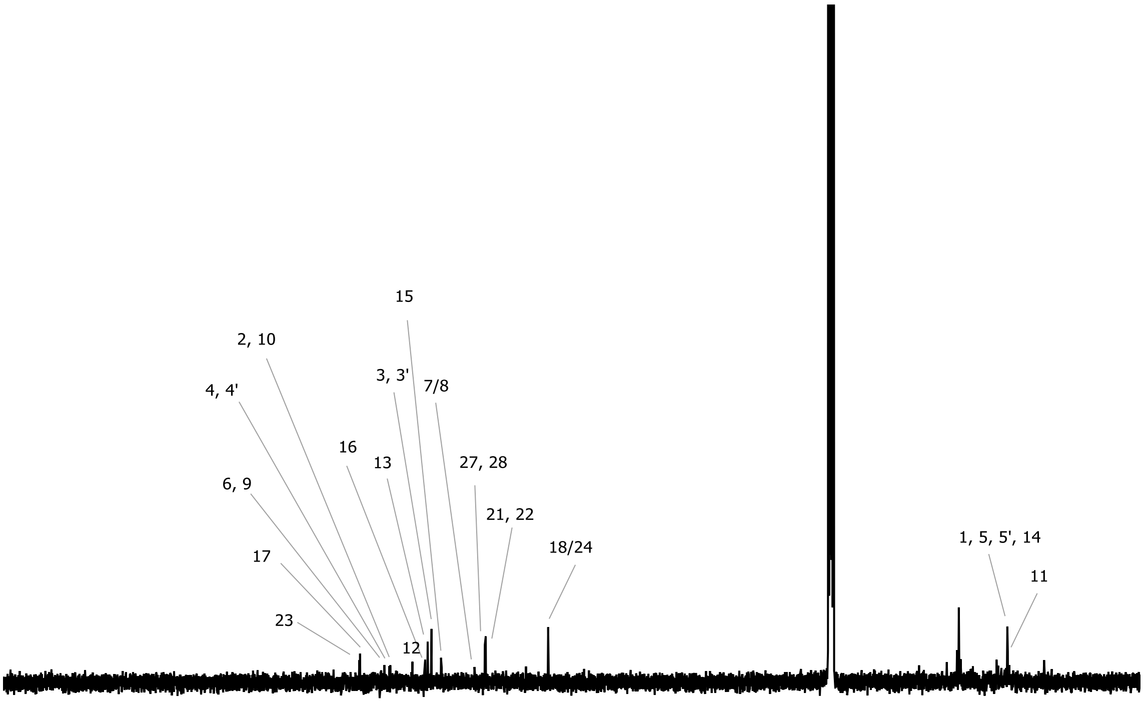

Figure S7. ^{13}C NMR spectrum (126 MHz, CD_2Cl_2, 23 °C) of compound **2**.

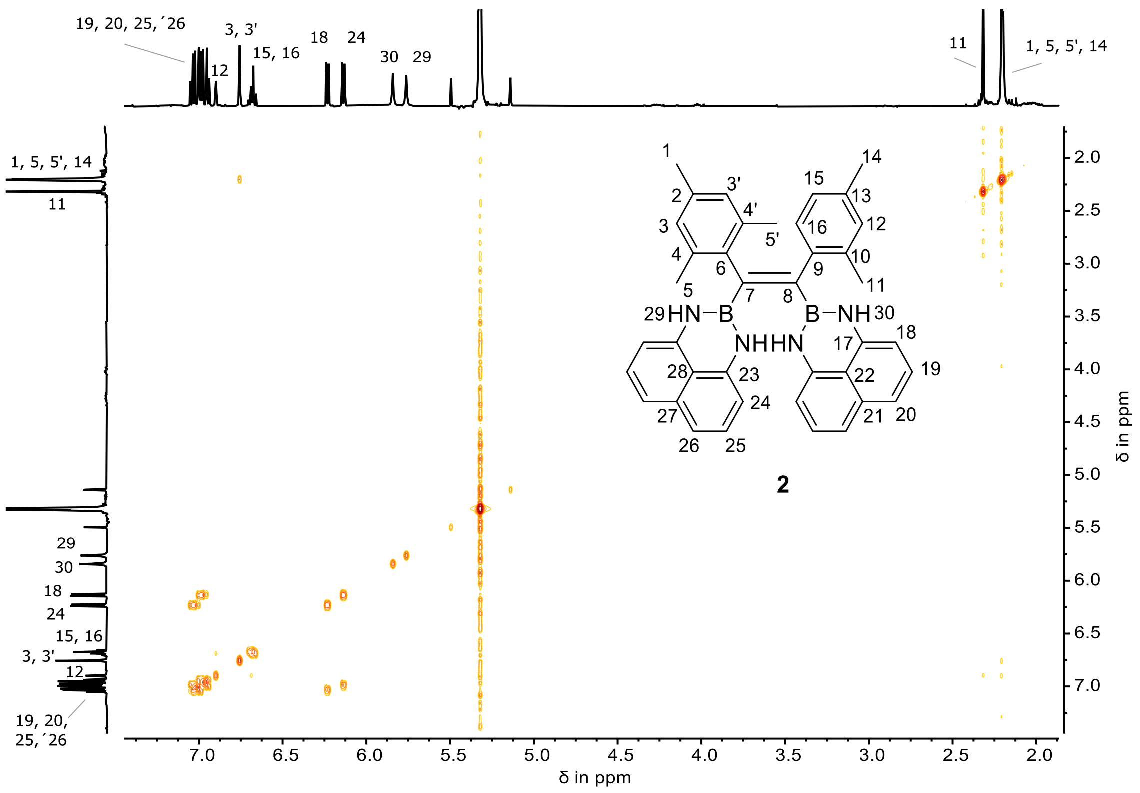

Figure S8. ^{1}H-^{13}C COSY NMR (500 MHz, CD_2Cl_2, 23 °C) spectrum of compound **2**.

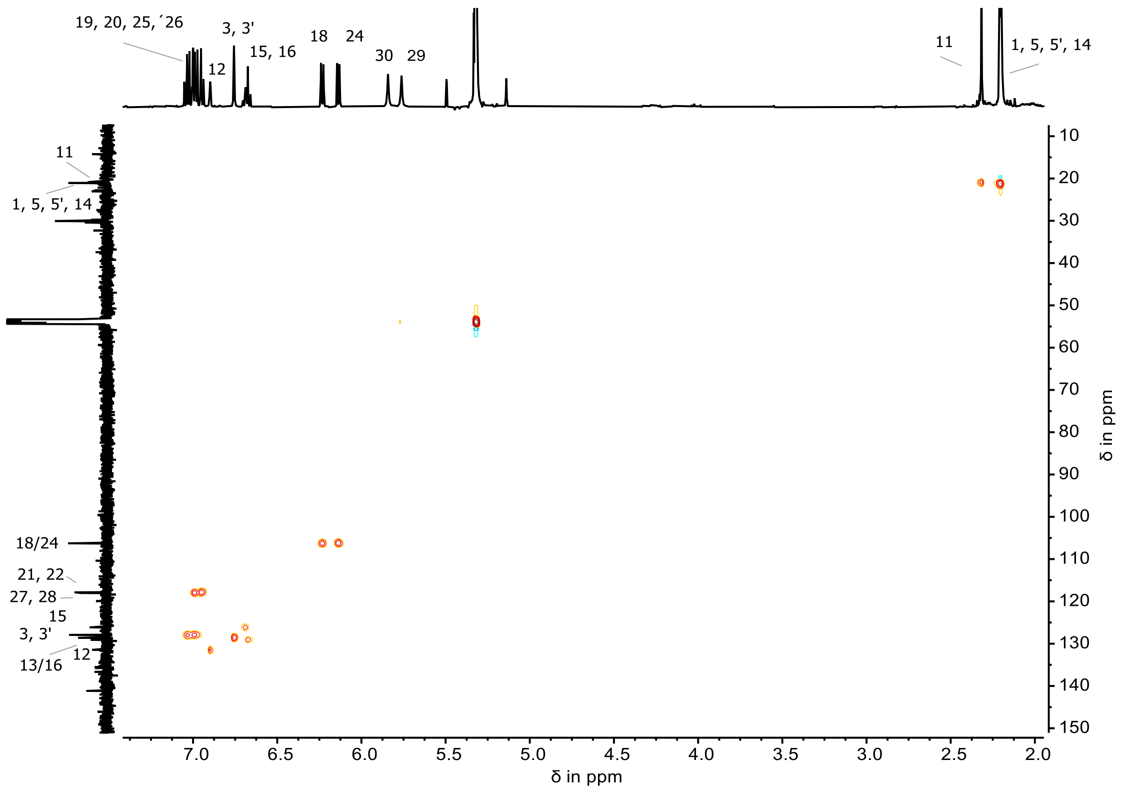

Figure S9. ^{1}H-^{13}C HSQC NMR spectrum (500 MHz, CD_2Cl_2, 23 °C) of compound **2**.

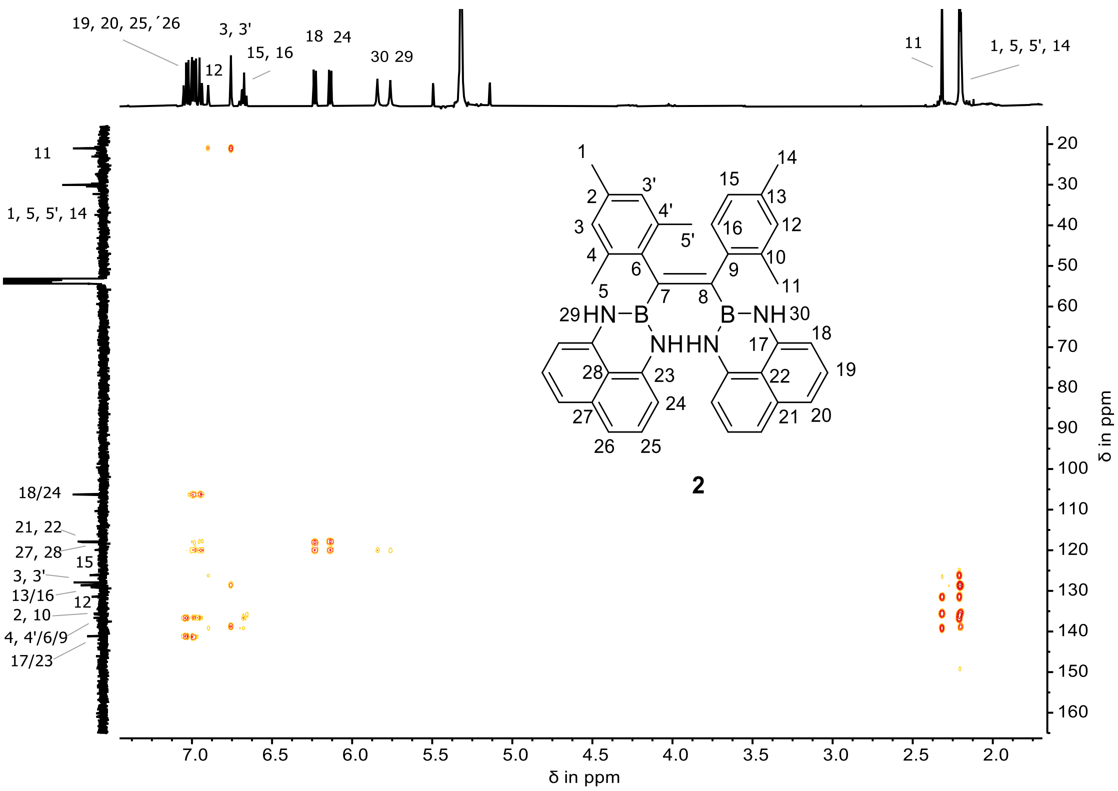

Figure S10. ^{1}H-^{13}C HMBC NMR spectrum (500 MHz, CD_2Cl_2, 23 °C) of compound **2**.

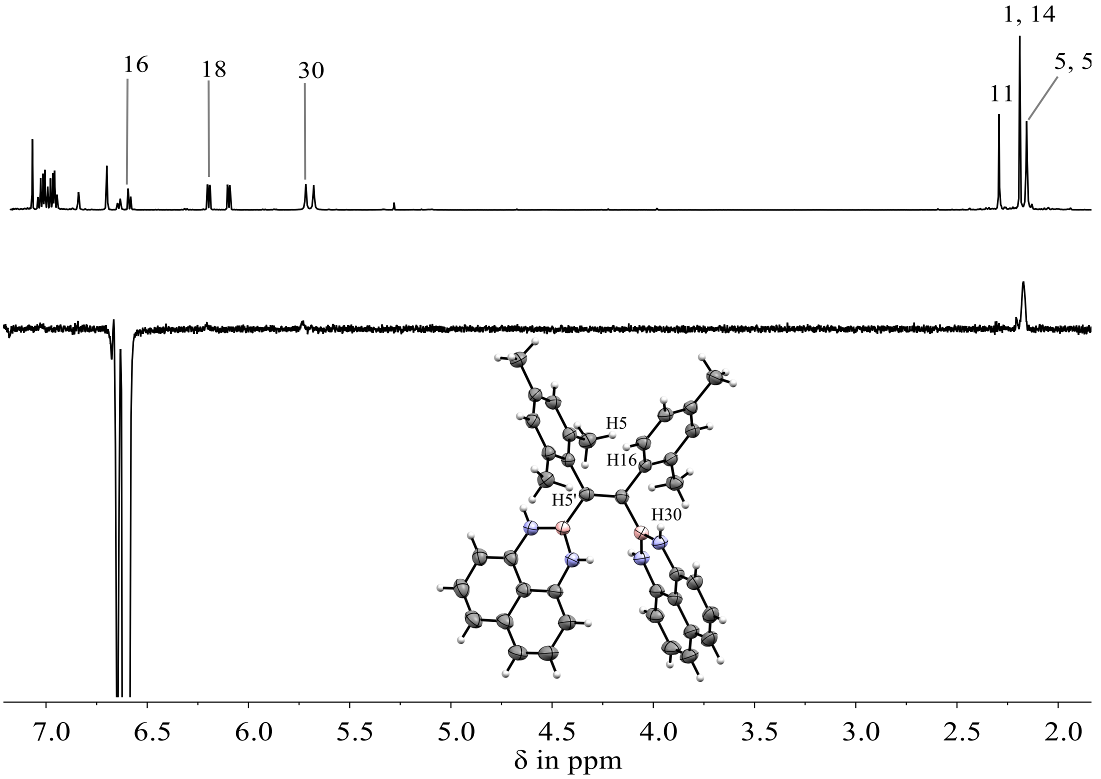

Figure S11. 1D NOE experiment ($CDCl_3$, 600 MHz, 40 °C) of Bdan **2** confirming *Z* configuration. Double bond configuration was assigned by the couplings of aromatic proton H-C16 (irradiated) with H-C5 and H-C5'.

3. Structures in the Crystalline State

Bdan 1

Single transparent and colorless block-shaped crystals of Bdan **1** recrystallized from a mixture of CH_2Cl_2 and *i*-Hex by slow evaporation technique. A suitable crystal with dimensions 0.23 × 0.16 × 0.11 mm^3 was selected and mounted on a mylar loop in perfluoroether oil on a SuperNova, Dual, Cu at home/near, Atlas diffractometer. The crystal was kept at a steady T = 153.1(4) K during data collection. The structure was solved with the Olex2.solve 1.5[9] solution program using iterative methods and by using Olex2 1.5[10] as the graphical interface. The model was refined with Olex2.refine 1.5[9] using full matrix least squares minimization on $\boldsymbol{F}^2$. Further crystallographic data are given in Table S1.

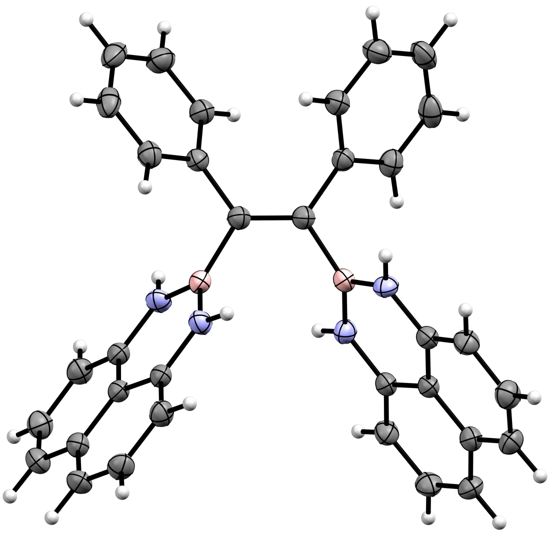

Figure S12. ORTEP representation of *Z*-**1** at 50% probability level.

Table S1. Crystallographic data of *Z*-**1**.

CCDC Number	2480142
Formula	$C_{34}H_{26}B_2N_4$
$D_{calc.}$/ g cm^{-3}	1.301
μ/mm^{-1}	0.589
Formula Weight	512.261
Color	clear light colourless
Shape	block-shaped
Size/mm^3	0.23×0.16×0.11
T/K	153.1(4)
Crystal System	orthorhombic
Space Group	*Pccn*
a/Å	10.0290(2)
b/Å	14.2600(3)
c/Å	18.2809(3)
α/°	90
β/°	90
γ/°	90
V/Å^3	2614.42(9)
Z	4
Z'	0.5
Wavelength/Å	1.54184
Radiation type	Cu K□
Θ_{min}/°	5.39
Θ_{max}/°	72.13
Measured Refl's.	7572
Indep't Refl's	2515
Refl's I≥2 σ(I)	2132
R_{int}	0.0371
Parameters	181
Restraints	0
Largest Peak	0.2490
Deepest Hole	-0.2706
GooF	1.0456
wR_2 (all data)	0.1357
wR_2	0.1231
R_1 (all data)	0.0545
R_1	0.0466

Bdan 2

Single transparent yellow block-shaped crystals of Bdan **2** recrystallised from a mixture of CH_2Cl_2 and heptane by solvent layering. A suitable crystal with dimensions $0.04 \times 0.03 \times 0.02$ mm^3 was selected and mounted on a mylar loop in perfluoroether oil on a XtaLAB Synergy R, HyPix-Arc 100 diffractometer. The crystal was kept at a steady T = 100.00(10) K during data collection. The structure was solved with the ShelXT 2018/2[11] solution program using dual methods and by using Olex2 1.5[9] as the graphical interface. The model was refined with ShelXL 2018/3[12] using full matrix least squares minimisation on $\boldsymbol{F}^2$. Further crystallographic data are given in Table S2.

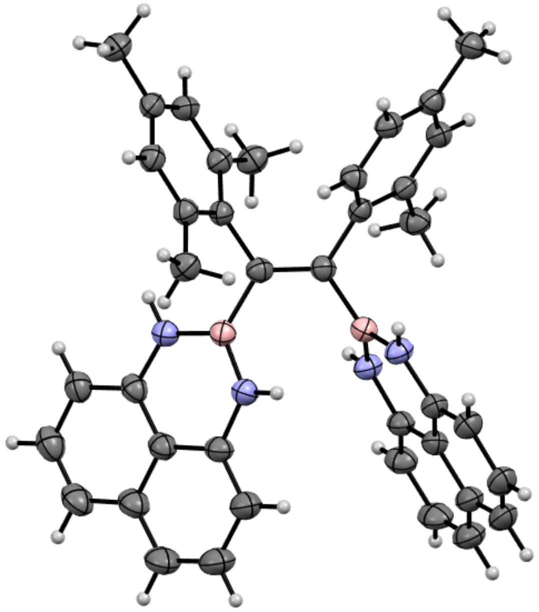

Figure S13. ORTEP representation of *Z*-**2** at 50% probability level.

Table S2. Crystallographic data of *Z*-**2**.

CCDC Number	2480141
Formula	$C_{39}H_{36}B_2N_4$
$D_{calc.}$/ g cm^{-3}	1.235
μ/mm^{-1}	0.550
Formula Weight	582.34
Color	clear light yellow
Shape	block-shaped
Size/mm^3	0.04×0.03×0.02
T/K	100.00(10)
Crystal System	monoclinic
Space Group	$P2_1/n$
a/Å	15.1173(4)
b/Å	11.5274(3)
c/Å	18.2929(5)
α/°	90
β/°	100.803(3)
γ/°	90
V/Å^3	3131.28(15)
Z	4
Z'	1
Wavelength/Å	1.54184
Radiation type	Cu K□
Θ_{min}/°	3.488
Θ_{max}/°	73.224
Measured Refl's.	20091
Indep't Refl's	5975
Refl's I≥2 σ(I)	4296
R_{int}	0.0582
Parameters	412
Restraints	0
Largest Peak	0.506
Deepest Hole	-0.253
GooF	1.041
wR_2 (all data)	0.1460
wR_2	0.1323
R_1 (all data)	0.0775
R_1	0.0529

Table S3. Bond lengths of the central double bond, and bonds connecting aryl and Bdan blades to the central double bond in crystal structures of tetraphenylethylene (TPE)[13], and Bdans **1** and **2** in Å.

	TPE	Bdan **1**	Bdan **2**
C=C double bond	1.346	1.357	1.362
C—aryl	1.490 – 1.501	1.495	1.496 – 1.504
C—B	-	1.577	1.579

Table S4. Torsion angles between central double bond and blades in crystal structures of TPE[13], and Bdans **1** and **2**.

	TPE	Bdan **1**	Bdan **2**
Aryl blade	46.5° – 56.7°	48.7° (α)	55.8° (α) / 64.4° (α’)
Bdan blade	-	69.8° (β)	70.8° (β) / 37.4° (β’)

a) Bdan **1** b) Bdan **2**

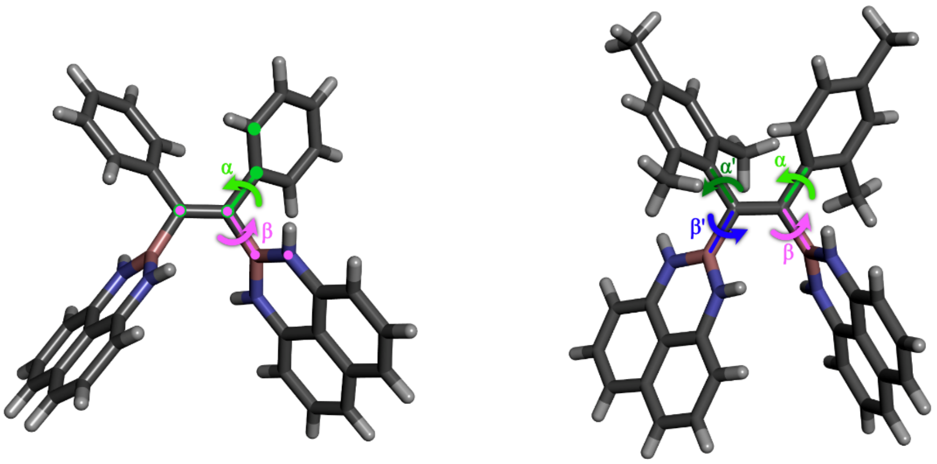

Figure S14. Illustration of measured torsion angles for a) Bdan **1** and b) Bdan **2**.

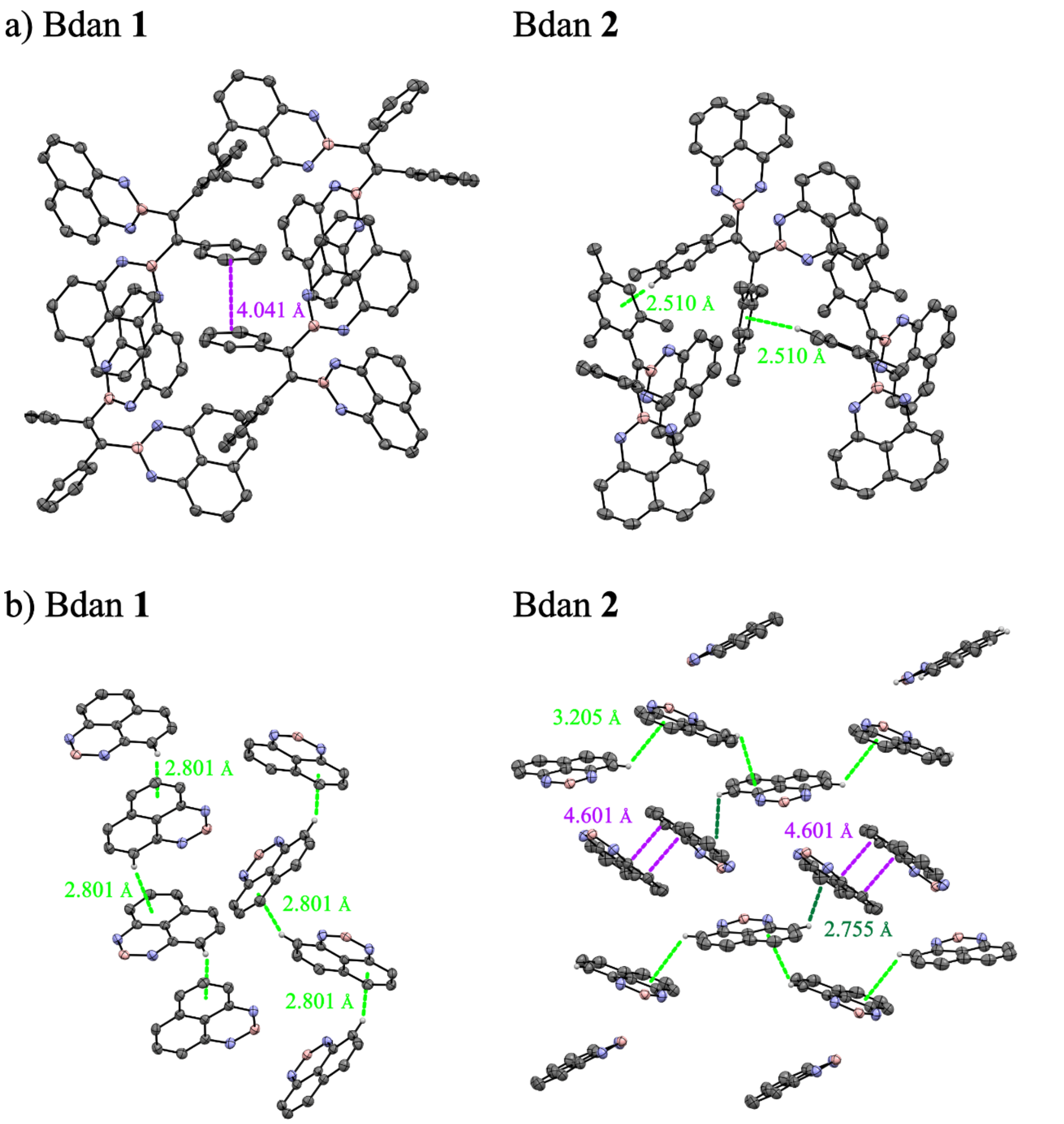

Figure S15. Dominant intermolecular aromatic interactions in the crystal structures of Bdan **1** and **2**. Distances of aromatic interactions were determined using centroids. Hydrogen atoms not involved in CH---π interactions are omitted for clarity. a) Interactions between aryl blades of Bdans **1** reveal parallel stacking arrangement sufficient for fluorescence quenching in the solid state, while aryl blades of Bdan **2** are aligned in a perpendicular fashion due to T-shaped CH---π interactions b) Interactions between Bdan blades of Bdans **1** and **2**. Bdan residues of Bdan **1** interact in a tilted T-shaped arrangement via CH---π, while Bdan blades of Bdan **2** arrange in a parallel manner, hinting at strong intermolecular aromatic interactions as reason for fluorescence quenching in the condensed state.

4. UV/Vis Spectra

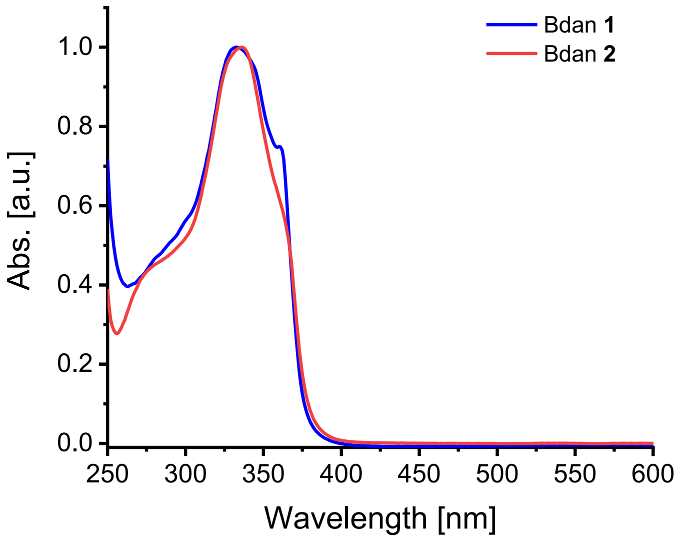

Figure S16. Normalized UV/Vis spectra of Bdan **1** and Bdan **2** measured in THF.

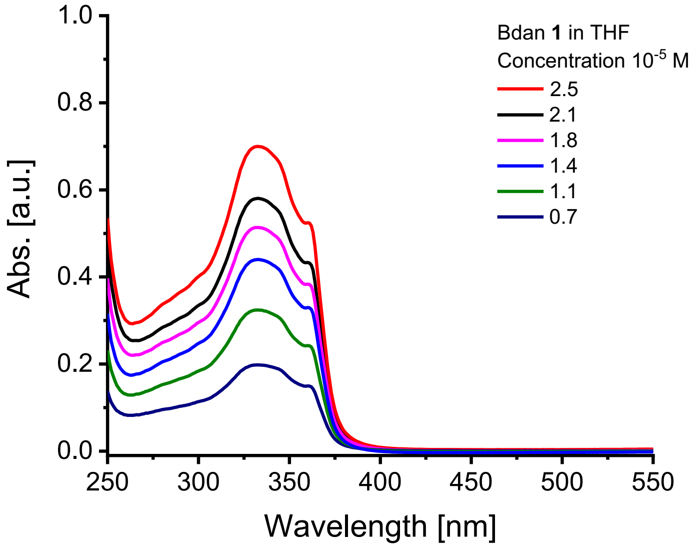

Figure S17. UV/Vis spectra of Bdan **1** in THF measured at different concentrations.

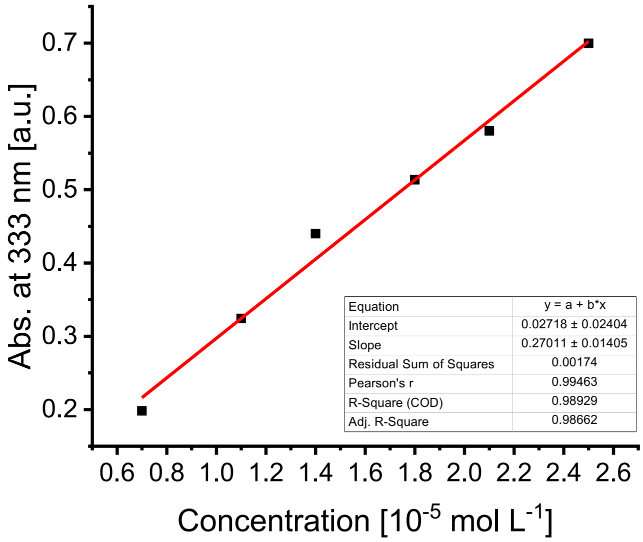

Figure S18. Concentration vs. absorbance plot of Bdan **1** in THF. The linear relationship between concentration and absorbance indicates that no aggregate is formed during the measurements. At 333 nm, ε is calculated as 27,011 L mol^{-1} cm^{-1}.

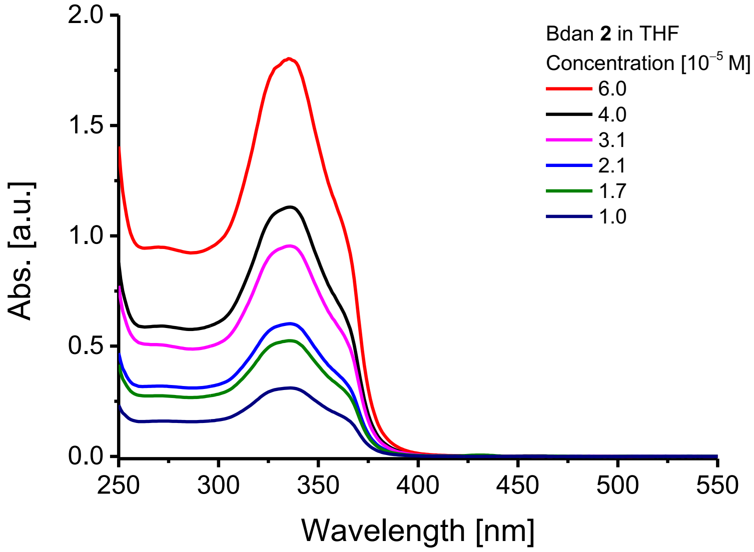

Figure S19. UV/Vis spectra of Bdan **2** in THF measured at different concentrations.

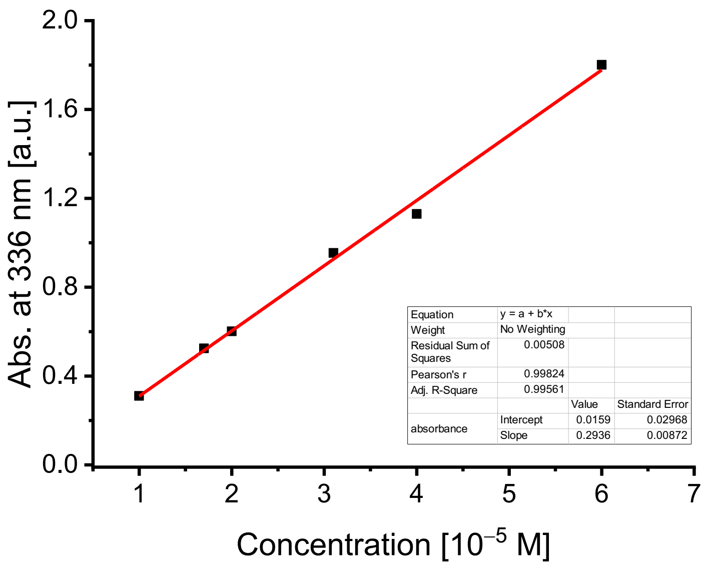

Figure S20. Concentration vs. absorbance plot of Bdan **2** in THF. The linear relationship between concentration and absorbance indicates that no aggregate is formed during the measurements. At 336 nm, ε is calculated as 29,366 L mol^{-1} cm^{-1}.

5. Fluorescence Measurements

5.1. Measurements in Solution

During the measurements of PL spectra with excitation wavelengths of 333 nm for Bdan **1** and 336 nm for Bdan **2**, emission signals were observed corresponding to each solvent used as medium. Due to the dependence of such signal on both the solvent and excitation wavelength, its origin can be attributed to inelastic scattering effects. Therefore, for the reported PL spectra of Bdans **1** and **2**, blank spectra of different solvents or mixtures with varying water fraction were measured and subtracted to remove scattering effects in the samples emission spectra. The results are shown in Figures S21–S24.

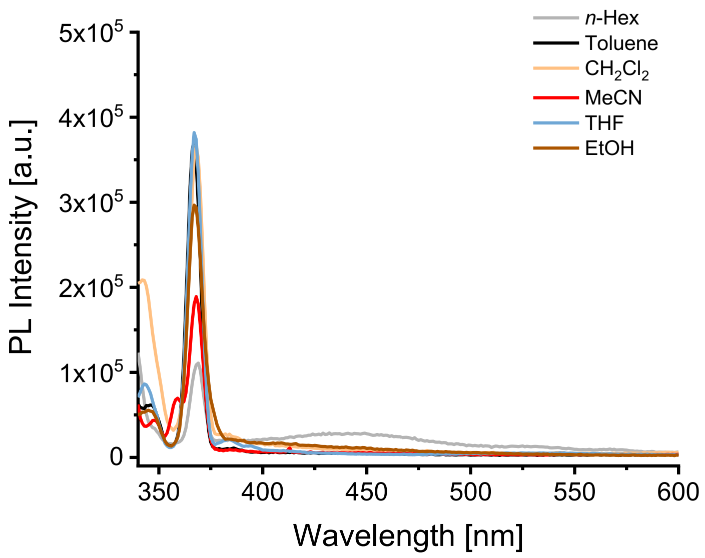

Figure S21 PL spectra of solvents used as blanks for an excitation wavelength of 333 nm.

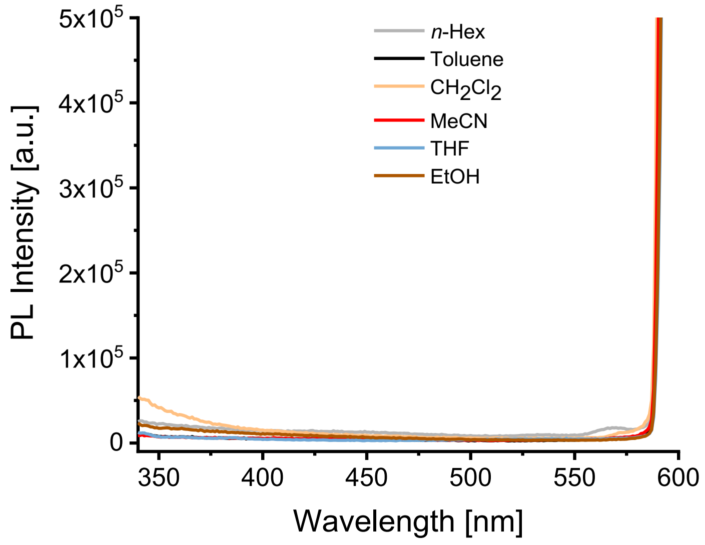

Figure S22. PL spectra of solvents used as blanks for an excitation wavelength of 300 nm.

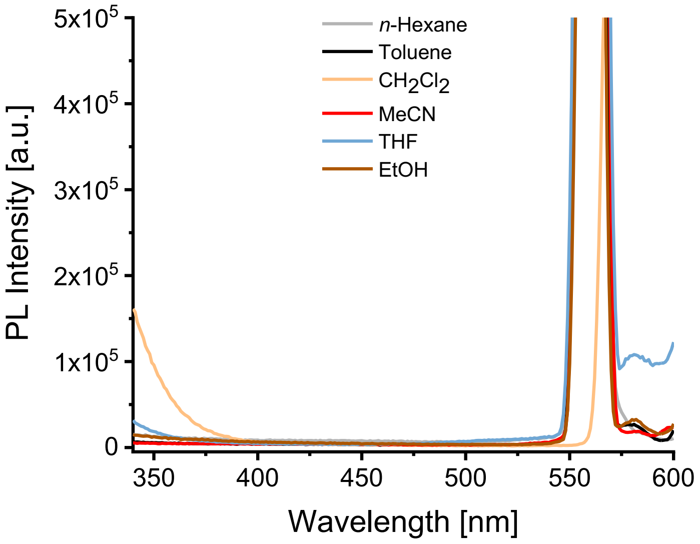

Figure S23. PL spectra of solvents used as blanks for an excitation wavelength of 280 nm.

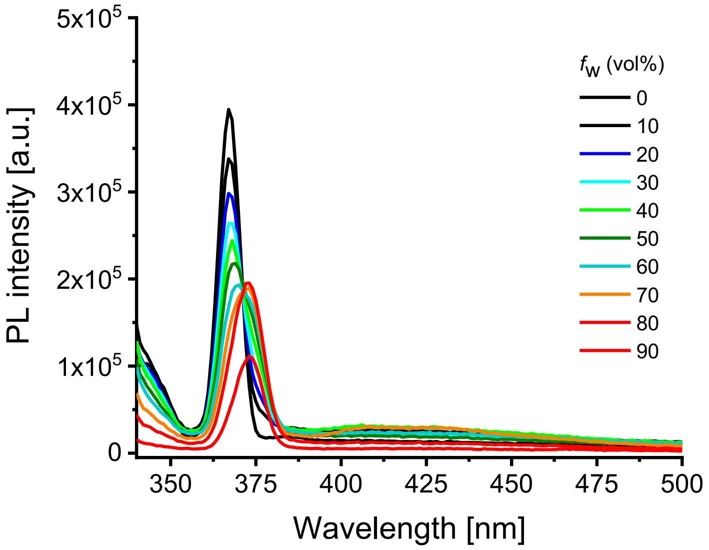

Figure S24. PL spectra of THF/water mixtures with water fractions (f_w = 0–90%) used as blanks for an excitation wavelength of 333 nm.

5.2. Measurements in Solid State

Fluorescence spectra of Bdan **1** and **2** were measured in the solid state and compared to the archetypal TPE AIEgen. A film of each compound was prepared by applying 10 µL of a CH_2Cl_2 solution onto a glass slide. After evaporating the solvent, the fluorescence spectra of the films were recorded at an angle of 75° with respect to the excitation light. As shown in Figure S25, Bdans **1** and **2** were non-emissive in the solid state upon excitation with 333 nm or 336 nm light, respectively. This suggests that ACQ effects dominate in the tightly packed state.

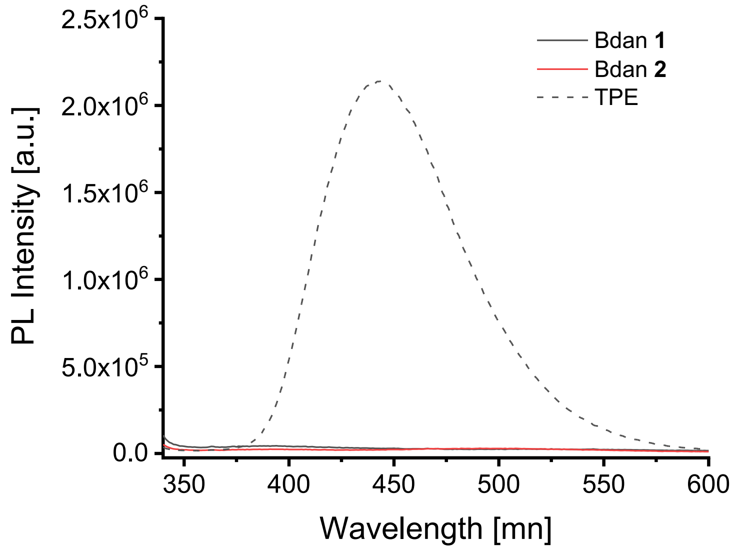

Figure S25. PL spectra of Bdan **1**, Bdan **2** and TPE in the solid state. Excitation wavelengths of 333 nm for TPE and Bdan **1**, and 336 nm for Bdan **2** were chosen.

5.3. Fluorescence Quantum Yield Measurements

To further quantify the AIE properties of Bdans **1** and **2**, fluorescence quantum yields (QY) of compounds **1** and **2** were measured in only THF (f_w = 0), in the respective solvent mixtures producing the most emissive states (f_w = 70% or 60%), and at high water fraction (f_w = 90%). A previously described quinine sulfate was used as standard with known fluorescence QY (ϕ_f = 54.6%).[14] Therefore, solutions of different concentrations of quinine sulfate in 0.1 M H_2SO_4 with absorptions between 0.02 and 0.08 were prepared and their corresponding PL spectra were recorded. For Bdan **1**, THF solutions with f_w = 0%, 70% and 90% were prepared in concentrations matching the absorptions of the quinine sulfate solutions at the excitation wavelength of 333 nm and PL spectra were recorded (Figure S26). Solutions of Bdan **2** were prepared and measured in a similar manner but for f_w = 0%, 60%, and 90% at an excitation wavelength of 336 nm (Figure S27).

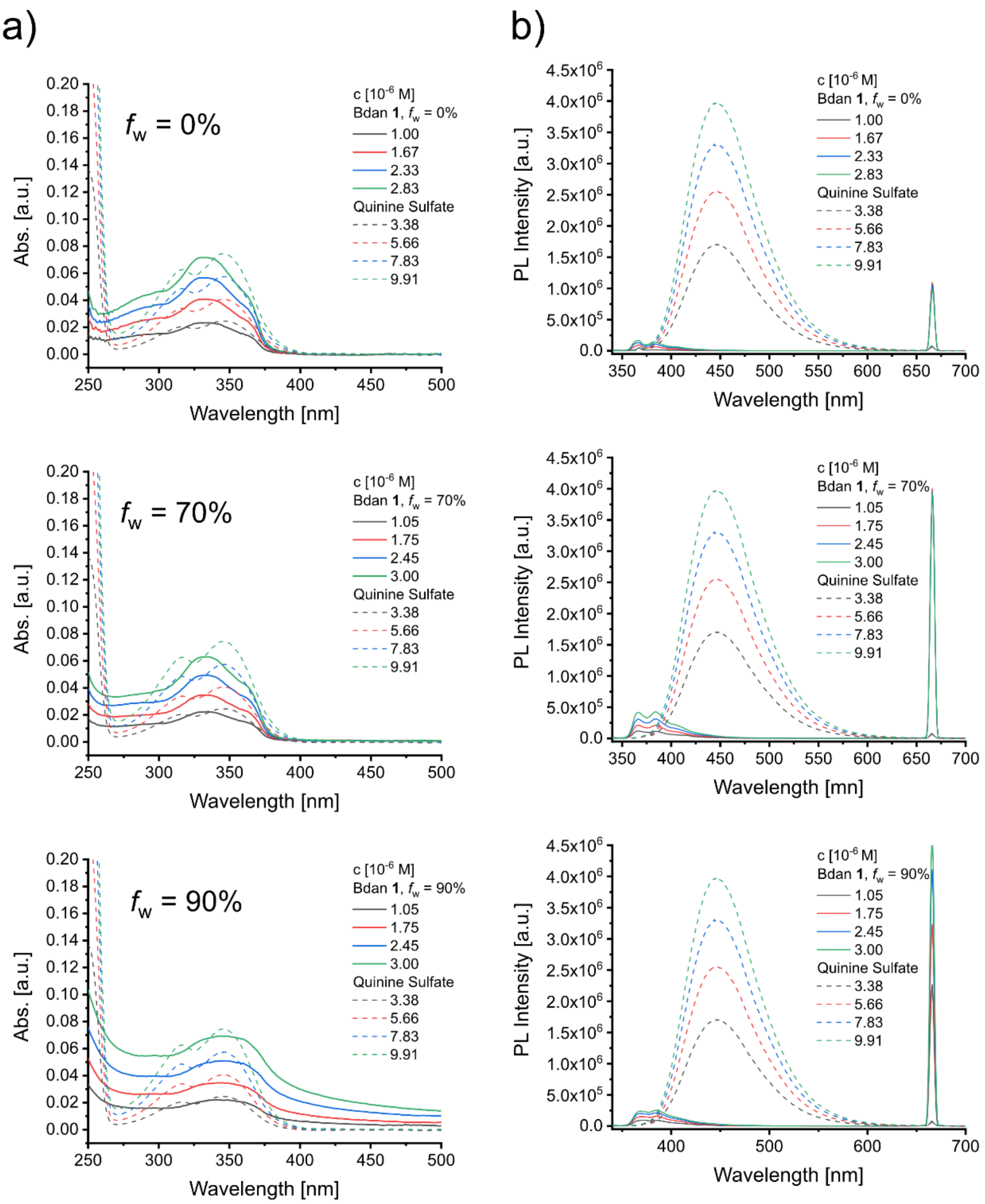

Figure S26. a) Absorption and b) corresponding emission spectra of solutions of Bdan **1** in THF/water (solid line) for f_w = 0%, 70% and 90% and quinine sulfate in 0.1 M H_2SO_4 (dashed line).

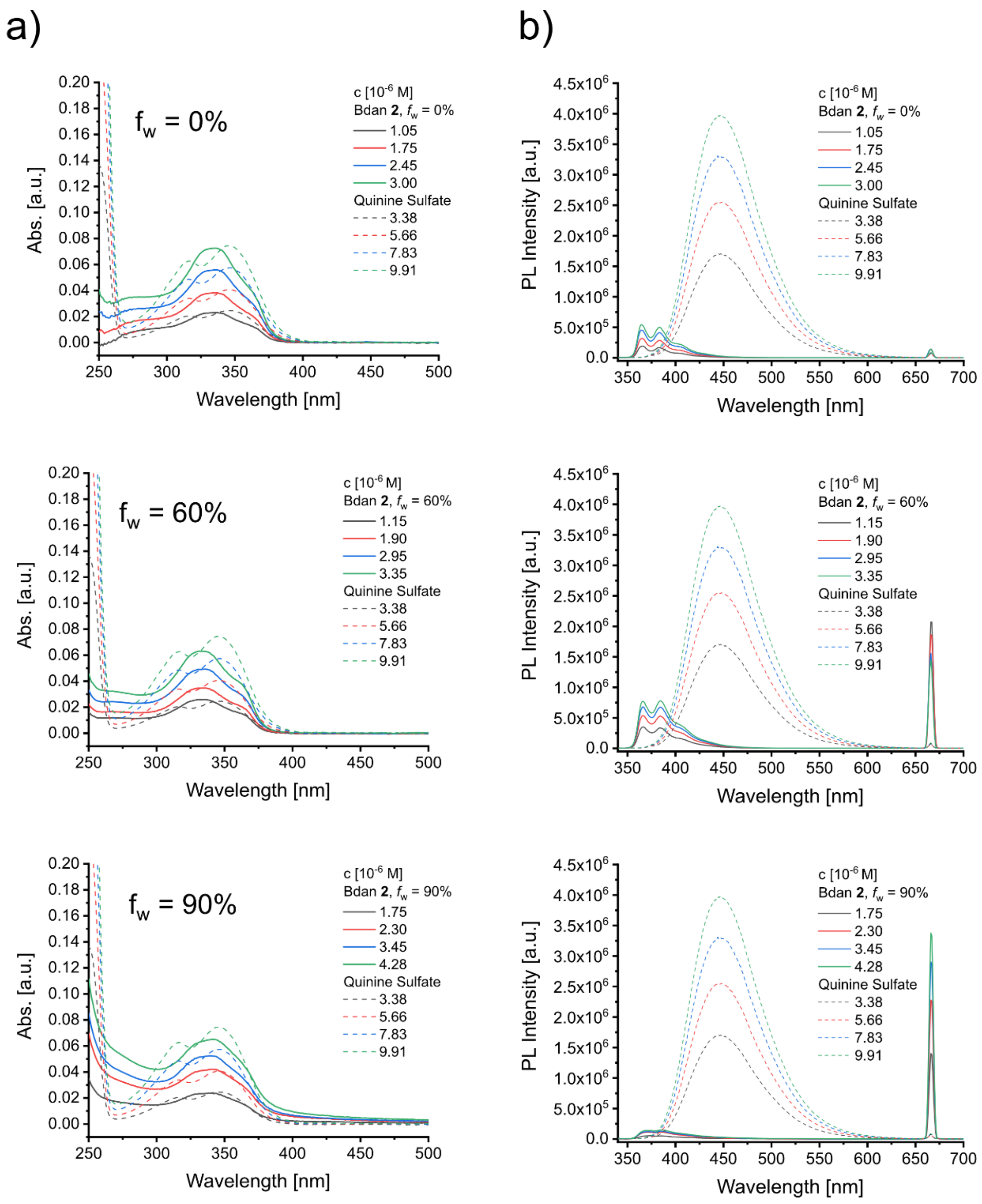

Figure S27. a) Absorption and b) corresponding emission spectra of solutions of Bdan **2** in THF/water (solid line) for f_w = 0%, 60% and 90% and quinine sulfate in 0.1 M H_2SO_4 (dashed line).

With the acquired absorption and emission spectra, fluorescence quantum yields $\phi_{f,x}$ of Bdans **1** and **2** could be determined by calculating the absorption factors at the excitation wavelength for each sample, integrating the emission spectrum and employing the following equation:[15]

$$\phi_{f,x} = \phi_{f,st} \cdot \frac{F_x}{F_{st}} \cdot \frac{f_{st}}{f_x} \cdot \frac{n_x^2(\lambda_{em})}{n_{st}^2(\lambda_{em})} \qquad \text{(Eq. 1)}$$

With the fluorescence quantum yield ϕ_f, the integral photon flux F_x, the absorption factor $f = 1 - 10^{-A(\lambda_{ex})}$, the refractive index of the solvent n, the wavelength λ and the indices x and st denoting sample and standard (quinine sulfate), respectively. Fluorescence quantum yields were calculated for solutions of investigated water fractions of Bdans **1** and **2**, averaged over the different employed concentration and the results are summarized in Table S5.

Table S5. Summary of calculated fluorescence quantum yields for different water fractions of Bdans **1** and **2**.

	ϕ_f (f_w = 0%, only THF)	ϕ_f (f_w = 60% (Bdan **2**) or 70% (Bdan **1**))	ϕ_f (f_w = 90%)
Bdan **1**	0.7 %	2.6%	2.3%
Bdan **2**	2.6%	5.5%	1.3%

6. Dynamic Light Scattering Measurements

To investigate both the particle size and size distribution of aggregates, dynamic light scattering (DLS) measurements were conducted with Bdan **1** and **2** in the THF/water solvent system producing the most emissive state (f_w = 70% or 60%, respectively) and at high water fractions (f_w = 90%), where different samples were tested by keeping the concertation within the 10^{-6} M regime (Figure S28 and S29). For Bdan **1** at intermediate f_w of 70%, the DLS data suggests that resulting aggregates present hydrodynamic radii ranging from 100 to 300 nm. Since the homogeneity of the sample can be quantified by the polydispersity index (PDI), for these measurements, a low PDI (0.02–0.04 over the tested concentrations) indicated a relatively sharp size distribution. Increasing the water fraction to 90% leads to a slightly broader distribution of aggregates in a comparable size regime (Figure S28).

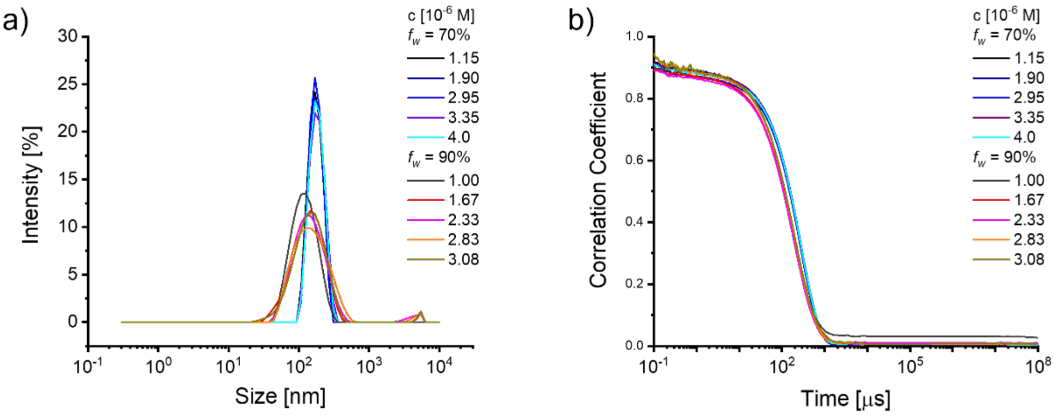

Figure S28. DLS data of Bdan **1** aggregates obtained in THF/water system with f_w = 70% and f_w = 90%. a) Size distribution and b) corresponding correlogram of different aggregated samples with concentrations within the 10^{-6} M regime.

When aggregates of Bdan **2** at the most emissive state at f_w = 60% were investigated by DLS, distinct behavior was clearly observed compared to Bdan **1** (Figure S29). Size distribution as well as the corresponding correlograms indicated the presence of two populations in the aggregated material: one population corresponded to aggregates of comparable size with Bdan **1** and another population consisting of particles with much smaller size (0.8 – 2.5 nm). Bdan **2** seems to be less prone to aggregation under these conditions and aggregates in a less-defined manner at intermediate f_w = 60% compared to Bdan **1** with lower degree of steric hindrance. When the water fraction was increased to f_w = 90%, the population of small aggregates vanished and only aggregates of comparable hydrodynamic radii to Bdan **1** (100–400 nm) were detected.

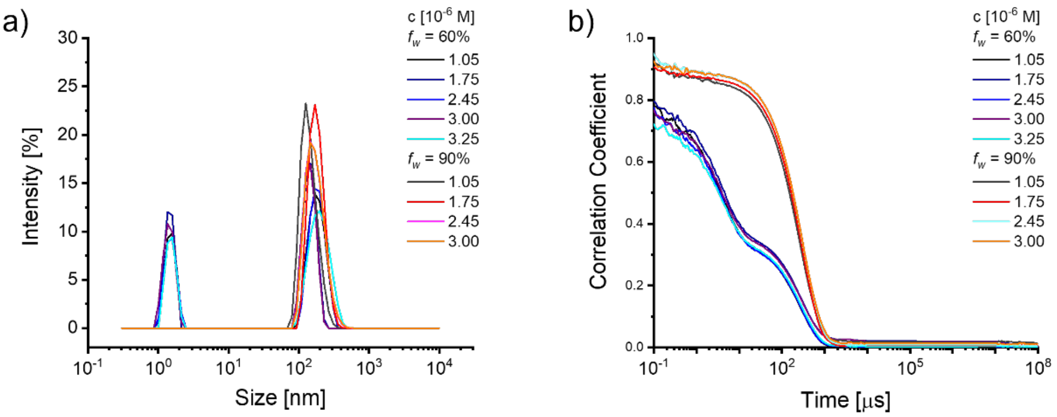

Figure S29. DLS data of Bdan **2** aggregates obtained in THF/water system with f_w = 60% and f_w = 90%. a) Size distribution and b) corresponding correlogram of different aggregated samples with concentrations within the 10^{-6} M regime.

7. Stability Tests on Bdans

NBN-doped 1,8-diaminonaphtyl boronamide (Bdan) is frequently used as a boron-masking form, since it stabilizes the empty *p* orbital of this main group element. When compared to other boron-protecting groups, Dan species stand out for their stability towards protodeboration under aqueous media.[16] In the present study, stability tests were conducted for Bdans **1** and **2** in their most emissive states using different THF/water mixtures (f_w = 60% or 70%). To track the changes that might indicate hydrolysis or further decomposition, ^{1}H NMR spectra were recorded at different time intervals (Figures S30 and S31). Similarly, the UV/Vis spectra of Bdans **1** and **2** in their most emissive states were measured before and after measuring photoluminescence to rule out hydrolysis or other degradation processes caused by photoirradiation during AIE experiments (Figures S32 and S33). The obtained results show that Bdans **1** and **2** present both hydrolytic and photochemical stability under these experimental conditions. Their most emissive states arise from motion restriction and aggregation effects rather than undesired by-products.

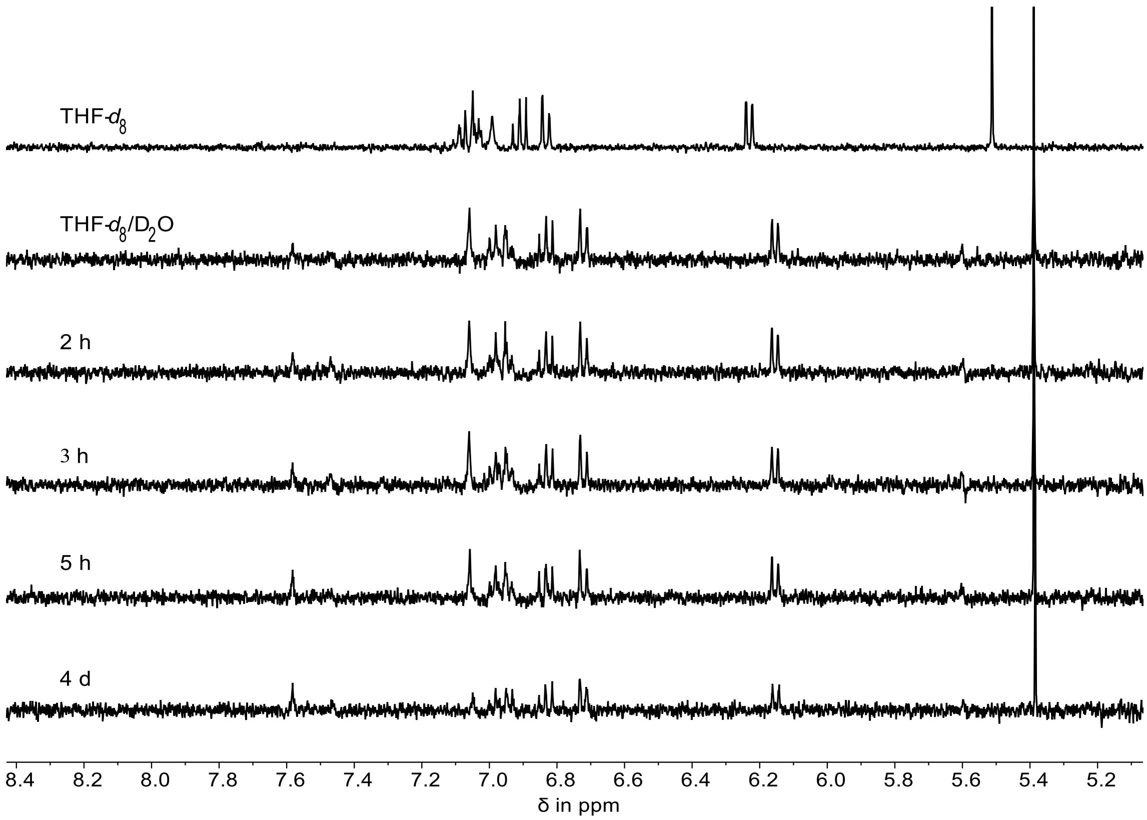

Figure S30. Stacked ^{1}H NMR spectra of Bdan **1** recorded at different time intervals in 70% D_2O/THF-d_8 mixture as the most emissive state.

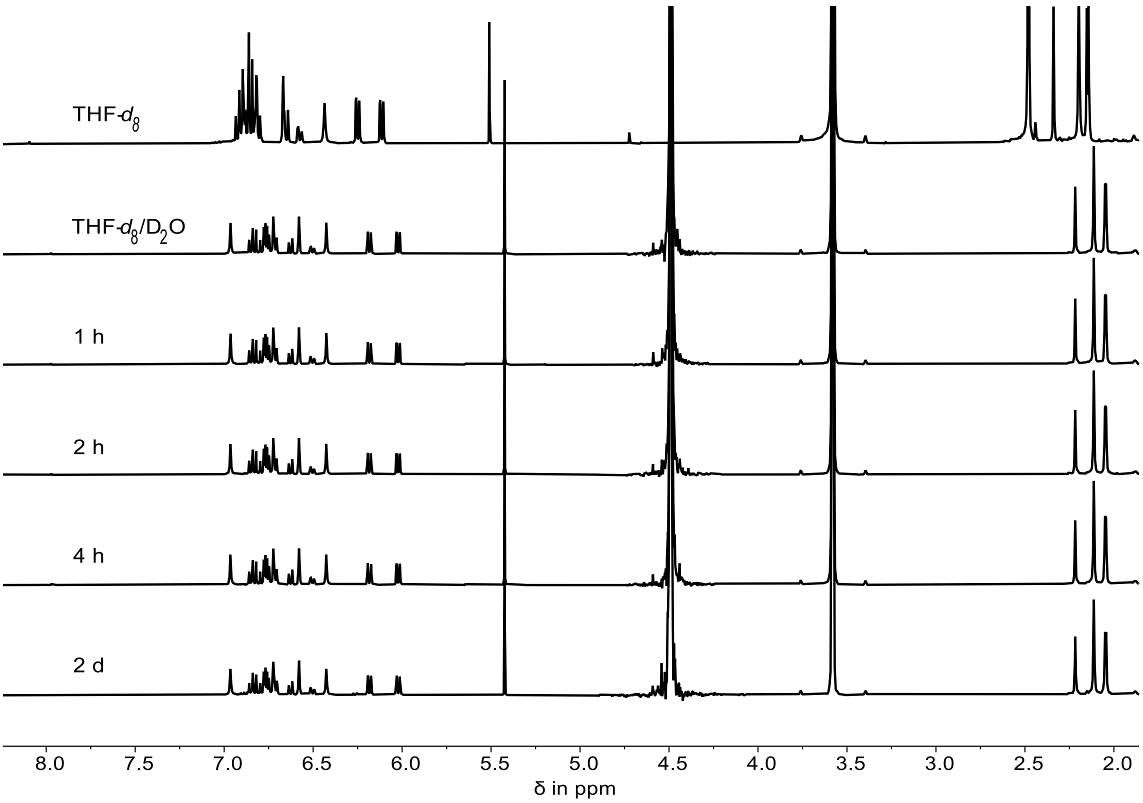

Figure S31. Stacked ^1H NMR spectra of Bdan **2** recorded at different time intervals in 60% D_2O/THF-d_8 mixture as the most emissive state.

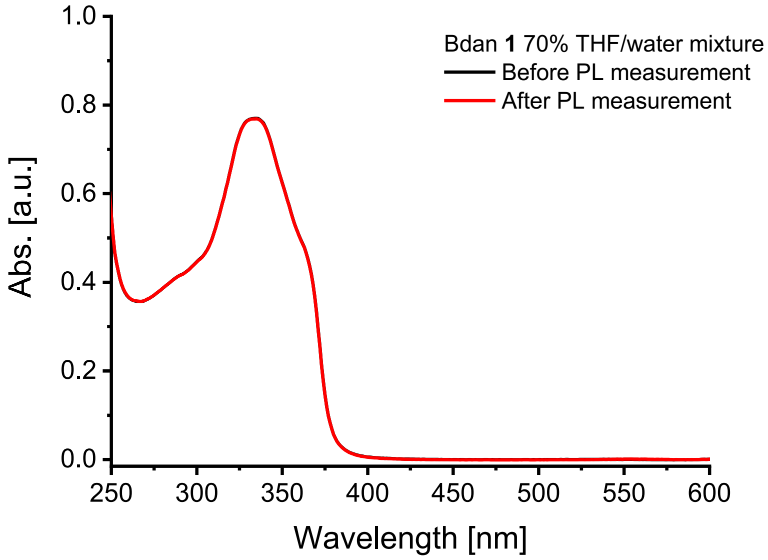

Figure S32. UV/Vis spectra of Bdan **1** in 70% water/THF mixture measured before and after fluorescence measurements.

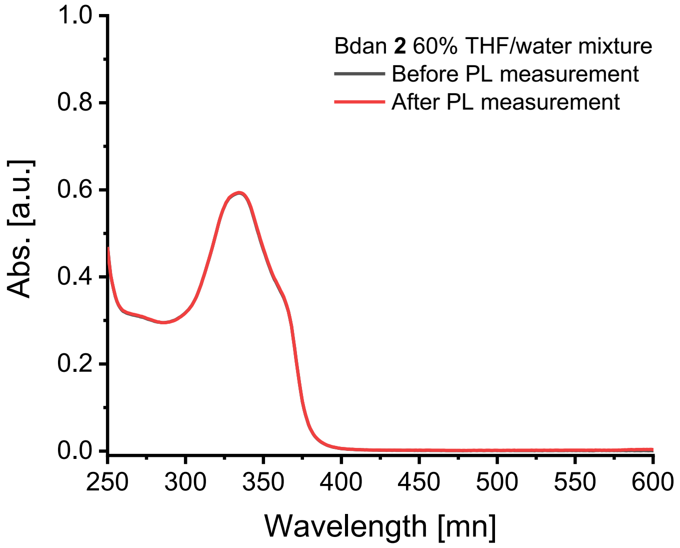

Figure S33. UV/Vis spectra of Bdan **2** in 60% water/THF mixture measured before and after fluorescence measurements.

8. Variable-Temperature ^{1}H NMR Measurements

Variable-Temperature ^{1}H NMR measurements were performed for Bdan **2** in $CDCl_3$ to reveal the rotational barrier associated with rotation of the single bond connecting the desymmetrized 2,4-dimethyl substituted phenyl ring to the central C=C bond (Figures S34–S36). Figure S34 shows that from 25 ºC to 0 ºC a single sharp peak is observed at ca. 6.68 ppm for H3 and H3' of the mesitylene fragment, indicating that the single bond rotation is fast on the NMR time scale. Starting from −10 ºC, this peak gets broadened with apparent decrease in its intensity upon cooling further till −40 ºC by 5 ºC steps. At −45 ºC, the rotational dynamics of the single bond shifts from fast to slow, reaching the maximum broadening and hindering the clear observation of two separate proton signals. This temperature is therefore estimated as coalescence temperature (T_c) for the single bond rotation process of the present system. Further cooling down till −64 ºC leads to a slow atropisomer equilibrium. For example, the splitting of H5 and H5' is clearly evidenced at −60 ºC and −64 ºC, yet a completely unambiguous assignment of these proton peaks located at 2.29 ppm and 1.90 ppm cannot be made (Figure S36).

The Gibbs energy of activation $\Delta G^{\neq}$ for two equally populated species can be easily calculated by the modified Eyring equation:[17] [18]

$$\Delta G^{\neq}(in\, J \cdot mol^{-1}) = R \cdot T_c \cdot [22.96 + \ln\left({}^{T_c}/_{\Delta v}\right) \qquad \text{(Eq. 2)}$$

Where T_c = coalescence temperature in K, R = 8.314 J $K^{-1}mol^{-1}$ (gas constant), an Δv = difference in chemical shifts ($\delta_A - \delta_B$) in Hz at the slow-exchange limit.

Using Eq. 2, with the coalescence temperature T_c = −45 ºC and Δv = 157.7 Hz at slow-exchange limit (measured at −64 ºC) the Gibbs energy of activation for rotation of the single bond, which connects the desymmetrized 2,4-dimethyl phenyl ring to the central C=C bond was calculated as $\Delta G^{\neq}$ = 10.58 kcal/mol.

At the coalescence temperature, the rate of exchange (k_{exch}) can be also calculated as:[19]

$$k_{exch} = \pi \cdot \Delta v/\sqrt{2} \qquad \text{(Eq. 3)}$$

Following Eq. 3, with Δv = 157.7 Hz at T_c = −45 ºC, k_{exch} for the related single bond rotation in Bdan **2** can be obtained as k_{exch} = 350.3 s^{-1}.

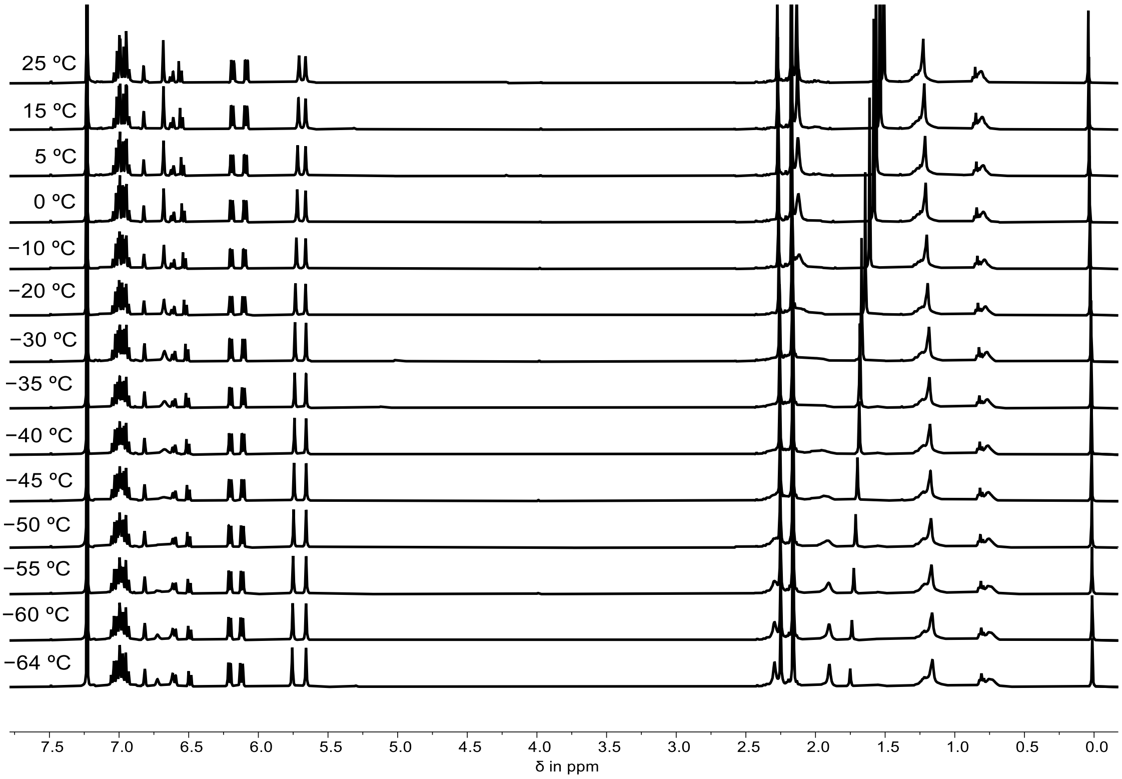

Figure S34. Stacked variable-temperature ^{1}H NMR spectra ($CDCl_3$, 400 MHz) of Bdan **2** measured from 25 ºC to −64 ºC.

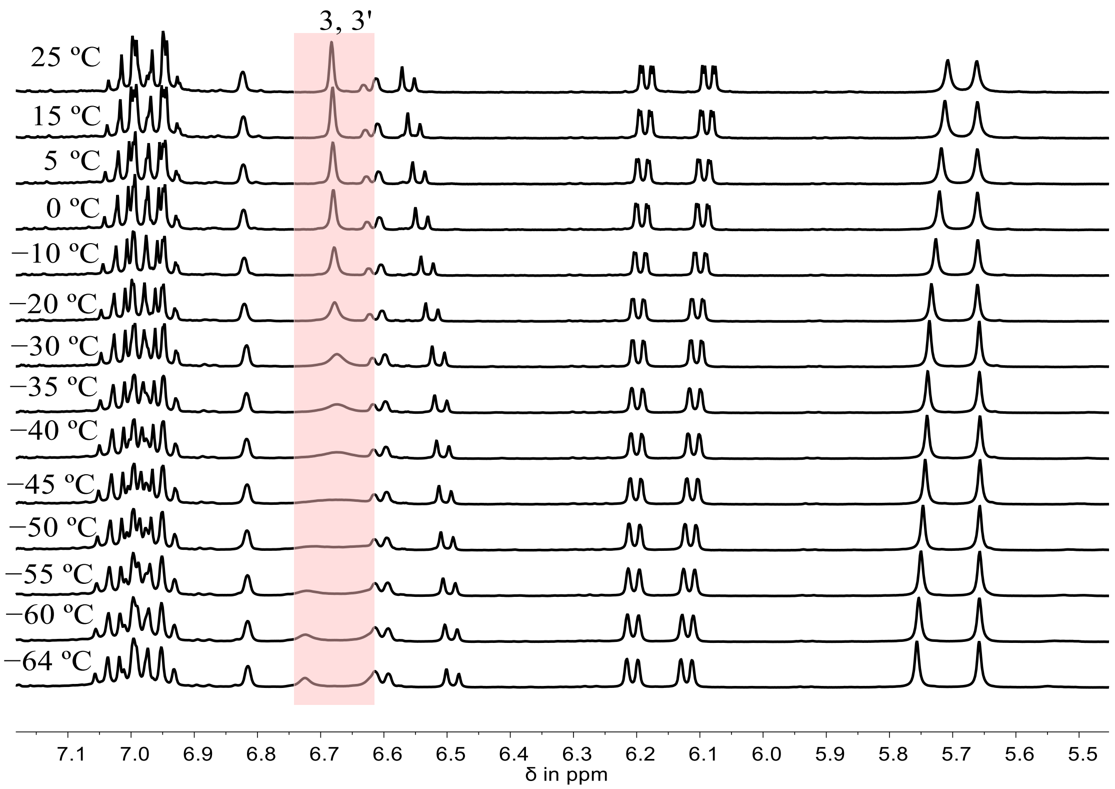

Figure S35. Stacked variable-temperature ^{1}H NMR spectra (aromatic region, $CDCl_3$, 400 MHz) of Bdan **2** measured from 25 ºC to −64 ºC.

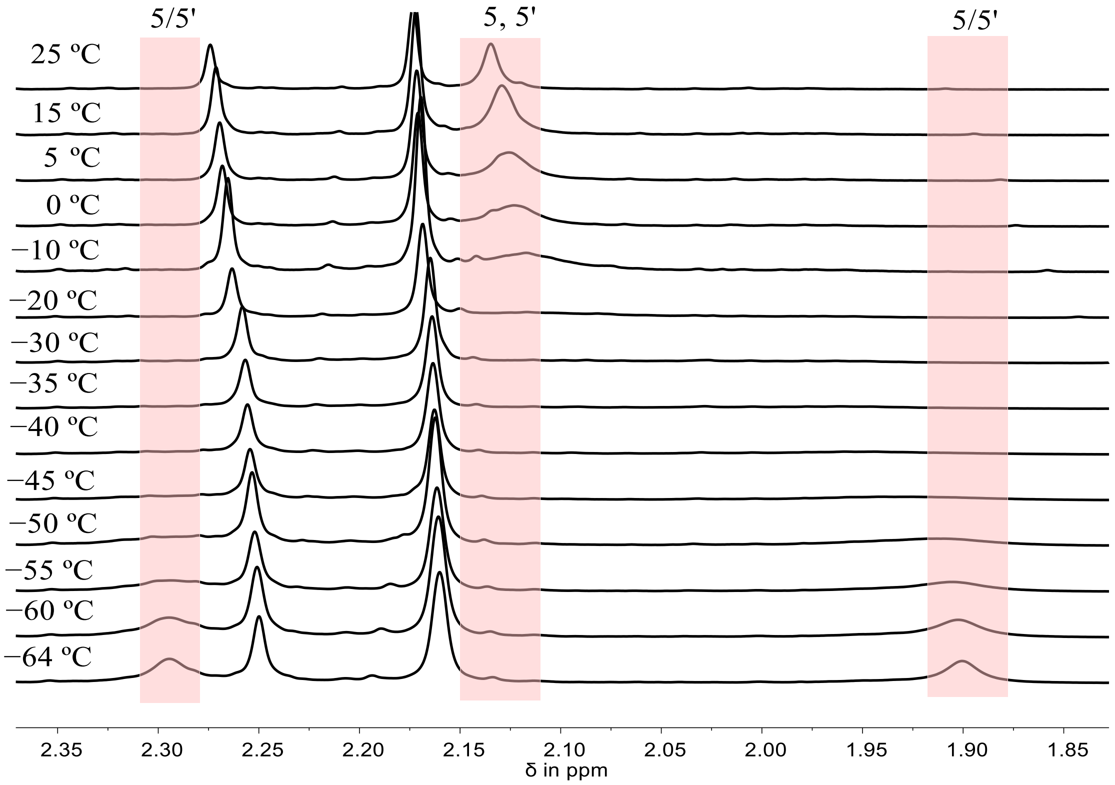

Figure S36. Stacked variable-temperature ^{1}H NMR spectra (aliphatic region, $CDCl_3$, 400 MHz) of Bdan **2** measured from 25 °C to −64 °C.

9. Theoretical Calculations

9.1. General Consideration for Conformational Analysis

The potential energy surface (PES) was explored for Bdans **1** and **2** by considering rotation around four single bonds (*a*, *b*, *c*, *d*; Figure S37) adjacent to the central double bonds in *Z*-configurations, since these degrees of freedom were determined to be significant. The PES was independently scanned by varying each torsional angle from 0° to 360° as well as taking 5° and a total of 36 steps in redundant coordinates at the semi-empirical PM6 level of theory. Once the low energy structures had been localized, they were fully optimized and related harmonic frequencies were calculated at the B3LYP/6-31+G(d,p) level of theory. Such PM6-DFT combined approach significantly reduced the computational cost and time necessary to conduct the conformational analysis. For the minima conformers, M06-2X/Def2TZVP and/or other levels of theory were also screened, as detailed in the following subsections.

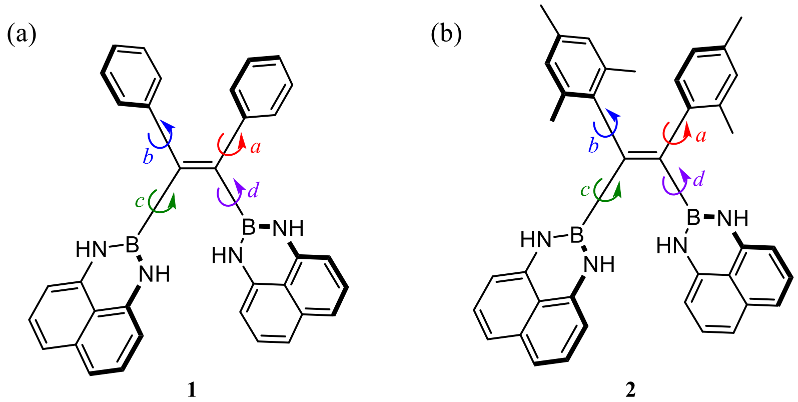

Figure S37. Torsion angles *a*, *b*, *c*, and *d* scanned for the conformational analyses of (a) Bdan **1** and (b) Bdan **2**.

9.2. Conformational Analysis on Bdan 1

In Bdan **1**, the steric repulsion among the *ortho* H atoms with respect to the single bonds of the central core makes each peripheral ring twist to the same direction. Therefore, this compound can adopt (*P*)- and (*M*)-handed dynamic propeller conformations, similar to the archetypal TPE molecule (Figure S38).

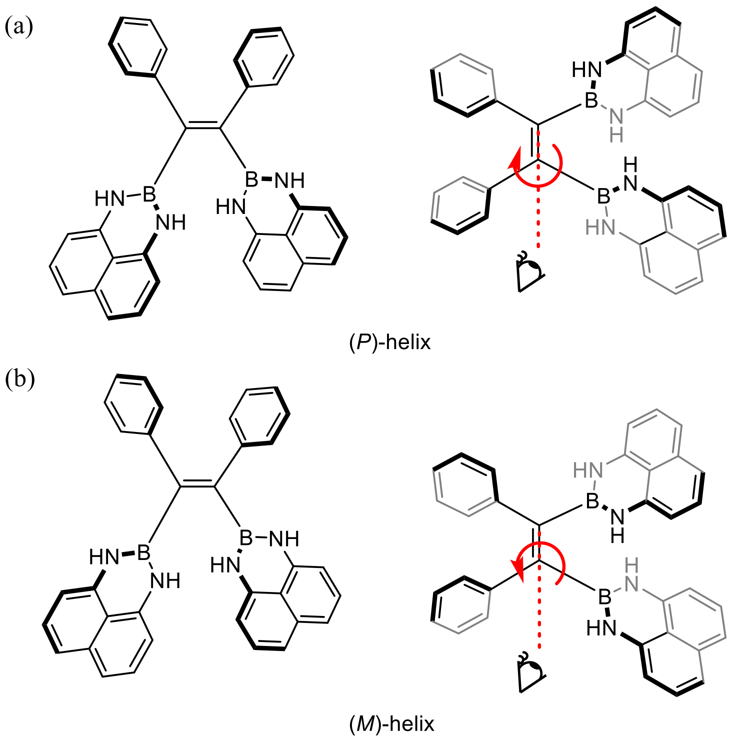

Figure S38. Propeller conformation in Bdan **1** adopting (a) (*P*)- and (b) (*M*)-helicities. The assignment was adopted from the literature for archetypal tetraphenylethylene.[20]

The systematic and independent scanning of *a*, *b*, *c*, and *d* angles at the PM6 level of theory and further geometry optimizations at the B3LYP/6-31+G(d,p) level of theory afforded in total two isomers with equal energy adopting (*P*)- and (*M*)-helicities for *Z*-configured double bond. After scanning of the first angle *a* and identifying two possible conformers adopting opposite helicities, for the remaining independent scans of *b*, *c*, and *d* angles, only the (*M*)-configured propeller conformation was considered for simplicity reasons and the obtained low energy structures were fully optimized. While the scanning of the angles *c*, and *d* seemed to afford slightly different structures for (*P*)-helicity, further optimization showed that these structures are identical both in geometry and energy. After scanning the *d* angle, the obtained minimum structure presenting (*P*)-helicity was further optimized and the frequency calculation was performed both at the B3LYP/6-31+G(d,p) and M06-2X/Def2TZVP levels of theory. According to the semi-empirical and DFT combined theoretical analysis, Bdan **1** presents a small conformational space, where different steps of angle scanning practically give only two conformers adopting (*P*)- or (*M*)-helicities. The results on the conformational analysis of Bdan **1** are summarized in Figure S39.

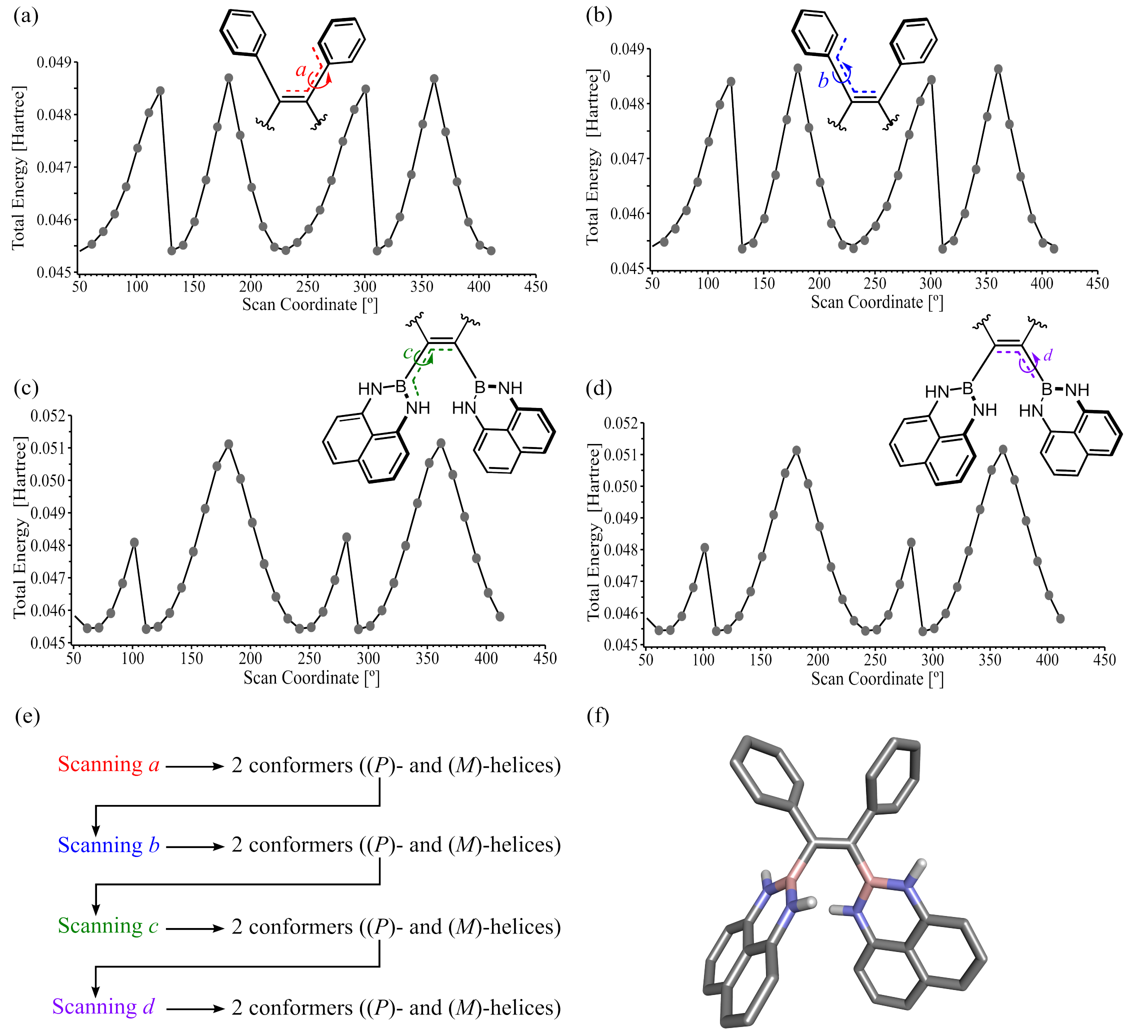

Figure S39. Results on conformational analysis of Bdan **1** combining PM6 with DFT methods. Scan coordinate vs. total energy plots obtained at the semi-empirical PM6 level of theory for (a) torsion angle *a*, (b) torsion angle *b*, (c) torsion angle *c*, (d) torsion angle *d*, (e) schematic representation of the minima conformers revealed from each independent scan, and (f) minimum conformer adopting (*P*)-helix obtained at the M06-2X/Def2TZVP level of theory. Note that each PES exploration was independently carried out by varying the torsional angles from 0º to 360º, where 5º and a total of 36 steps in redundant coordinates were taken.

9.3. Conformational Analysis on Bdan 2

In addition to propeller chirality, the desymmetrization with 2,4-dimethyl substitution of one of the phenyl blades gives rise to atropisomerism in Bdan **2**. The compound thus adopts four different isomers for *Z*-configured double bond. These are *Z*-(*P*)-(*R*a)/*Z*-(*M*)-(*S*a) and *Z*-(*M*)-(*R*a)/*Z*-(*P*)-(*S*a) enantiomeric pairs, as depicted in Figure S40.

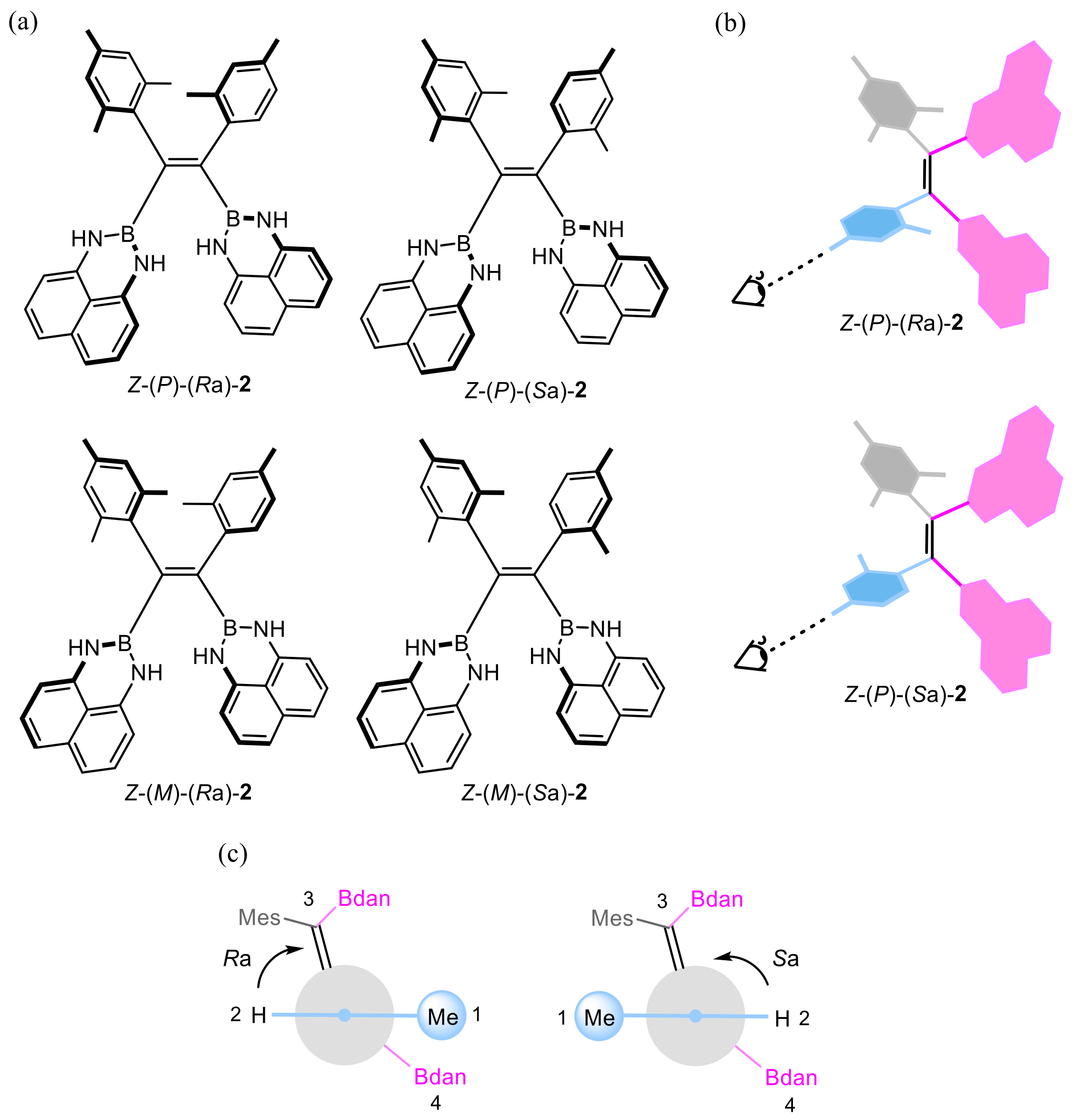

Figure S40. (a) All possible stereoisomers of Bdan **2** for fixed *Z*-configured double bond. (b) Exemplified absolute stereochemistry assignment for *Z*-(*P*)-(*R*a) (top) and *Z*-(*P*)-(*S*a) (bottom) isomers of Bdan **2**. (c) Newman projections for *Z*-(*P*)-(*R*a) (left) and *Z*-(*P*)-(*S*a) (right) atropisomers.

The systematic and independent scanning of *a*, *b*, *c*, and *d* angles at the PM6 level followed by geometry optimizations at the B3LYP/6-31+G(d,p) level of theory showed that the conformational space of Bdan **2** is rather flat, where a single minimum structure could be ascribed for each enantiomeric pair (Figure S41). Starting off with the torsion angle *a*, *Z*-(*P*)-(*R*a), *Z*-(*M*)-(*S*a), *Z*-(*M*)-(*R*a), and *Z*-(*P*)-(*S*a) were encountered as all expected isomers. For sake of simplicity, only one enantiomer of *Z*-(*P*)-(*R*a)/*Z*-(*M*)-(*S*a) and *Z*-(*M*)-(*R*a)/*Z*-(*P*)-(*S*a) isomers were considered for the subsequent angle scans. The scans of *b*, *c*, and *d* angles and comparison of each individual PES exploration steps led

to identification of two different conformers differing in helicities, beginning with i.e. *Z*-(*P*)-(*R*a) or *Z*-(*P*)-(*S*a). After scanning the *d* angle, the obtained minima structures *Z*-(*M*)-(*S*a) and *Z*-(*M*)-(*R*a) were fully optimized using several DFT methods in addition to the B3LYP/6-31+G(d,p) and M06-2X/Def2TZVP, which served as benchmark methods. As can be seen from Table S6 that *Z*-(*P*)-(*R*a)/*Z*-(*M*)-(*S*a) is the global minimum, whereas *Z*-(*P*)-(*S*a)/*Z*-(*M*)-(*R*a) possesses higher energy by 1.90–2.39 kcal/mol depending on the level of theory.

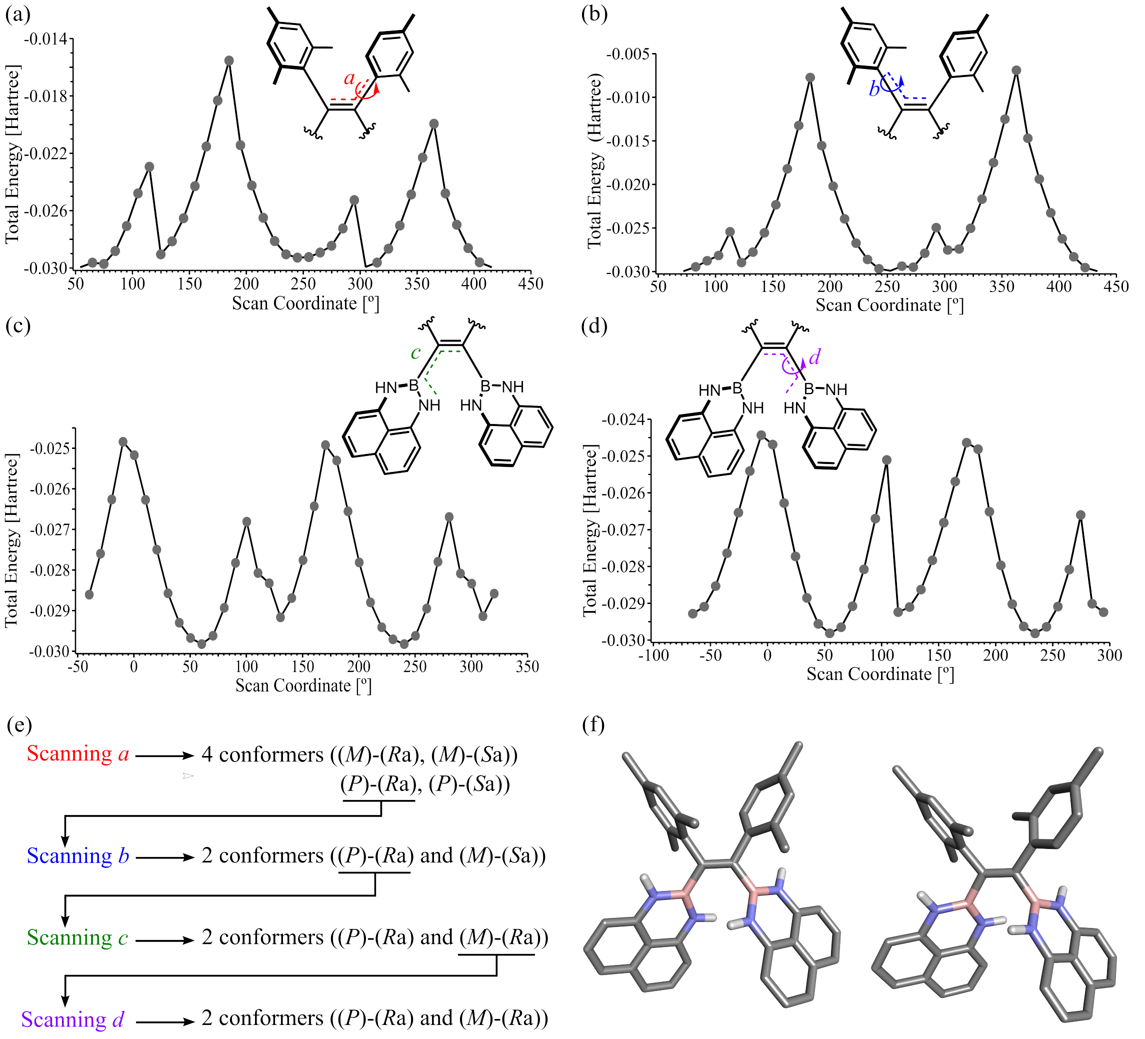

Figure S41. Results on conformational analysis of Bdan **2** combining PM6 with DFT methods. Scan coordinate vs. total energy plots obtained at the semi-empirical PM6 level of theory for (a) torsion angle *a*, (b) torsion angle *b*, (c) torsion angle *c*, (d) torsion angle *d*, (e) schematic representation on the minima conformers revealed from each scan for *Z*-configured double bond. For clarity reasons, only the (*R*a)-configured atropisomer is shown for the results on scanning of torsion angles *b*–*d*. (f) minima conformers Z-(*M*)-(*S*a)-**2** (left) and Z-(*M*)-(*R*a)-**2** obtained at the M06-2X/Def2TZVP level of theory. Note that each PES exploration was independently carried out by varying the torsional angles from 0° to 360°, where 5° and a total of 36 steps in redundant coordinates were taken.

Table S6. Theoretical description of *Z*-(*M*)-(*S*a) and *Z*-(*M*)-(*R*a) isomers of Bdan **2** at different DFT levels.

Level of theory	***Z*-(*M*)-(*S*a)-2**	***Z*-(*M*)-(*R*a)-2**
B3LYP/6-31G(d)	0.00	2.02
B3LYP/6-31+G(d,p)	0.00	2.07
B3LYP/6-311G(d,p)	0.00	2.11
CAM-B3LYP/6-311G(d,p)	0.00	2.10
B3LYP-D3BJ/6-311G(d,p)	0.00	1.90
M06-2X/Def2TZVP	0.00	2.39

9.4. Non-Covalent Interactions and Reduced Density Gradient Analysis

Non-covalent interaction (NCI) analysis was performed using the Multiwfn3.8 package.[4] The key quantity for this method is the reduced density gradient (RDG), which is calculated using Eq. 4:

$$\mathrm{RDG(r)} = \frac{1}{2(3\pi^2)^{\frac{1}{3}}} \frac{|\nabla\rho(r)|}{\rho(r)^{\frac{4}{3}}} \qquad \text{(Eq. 4)}$$

Where ρ(r) stands for the electron density. In case of non-covalent interactions, these weak forces are characterized by their low electron-density features and reduced gradients getting closer to zero. Covalent bonds, on the other hand, present reduced gradients approaching zero but higher electron densities and regions being far from the molecule possess low electron densities. Using these quantities, regions of non-covalent interactions can be therefore unequivocally determined. The sign of the second-largest eigenvalue of the Hessian (sign(λ_2) is decisive for the type of interaction, where a positive sign means steric effects or other repulsive interactions and a negative sign is a characteristics of attractive interactions like hydrogen bonding (Figure S42). Bearing in mind that the electron density is higher in regions of stronger interactions and lower in regions of weaker interactions (e.g., van der Waals interactions), it is possible to distinguish different non-covalent interaction types upon plotting the RDG vs. sign(λ_2)ρ. The isosurfaces of reduced density gradient discloses the areas of intramolecular interactions existing in a molecule.

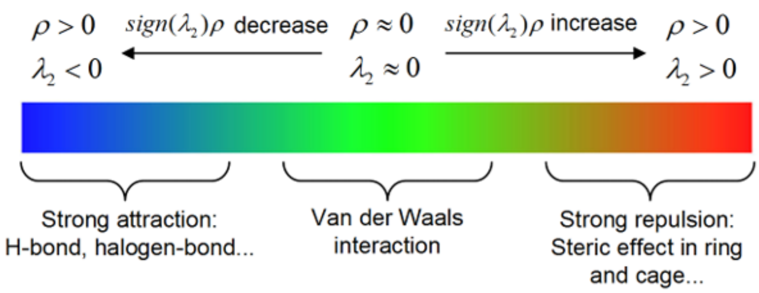

Figure S42. Coloring scheme for RDG scatter plots and isosurfaces.

The isosurfaces of weak interaction regions were visualized for the minima conformers of Bdan **1** and Bdan **2** at the M06-2X/Def2TZVP level of theory by setting (200·200·200) grid points in each direction. In case of hindered Bdan **2**, the NCI-RDG analysis was performed for two possible isomers *Z*-(*M*)-(*S*a) and *Z*-(*M*)-(*R*a) differing in atropisomeric handedness. The results are depicted in Figures S43 and S44.

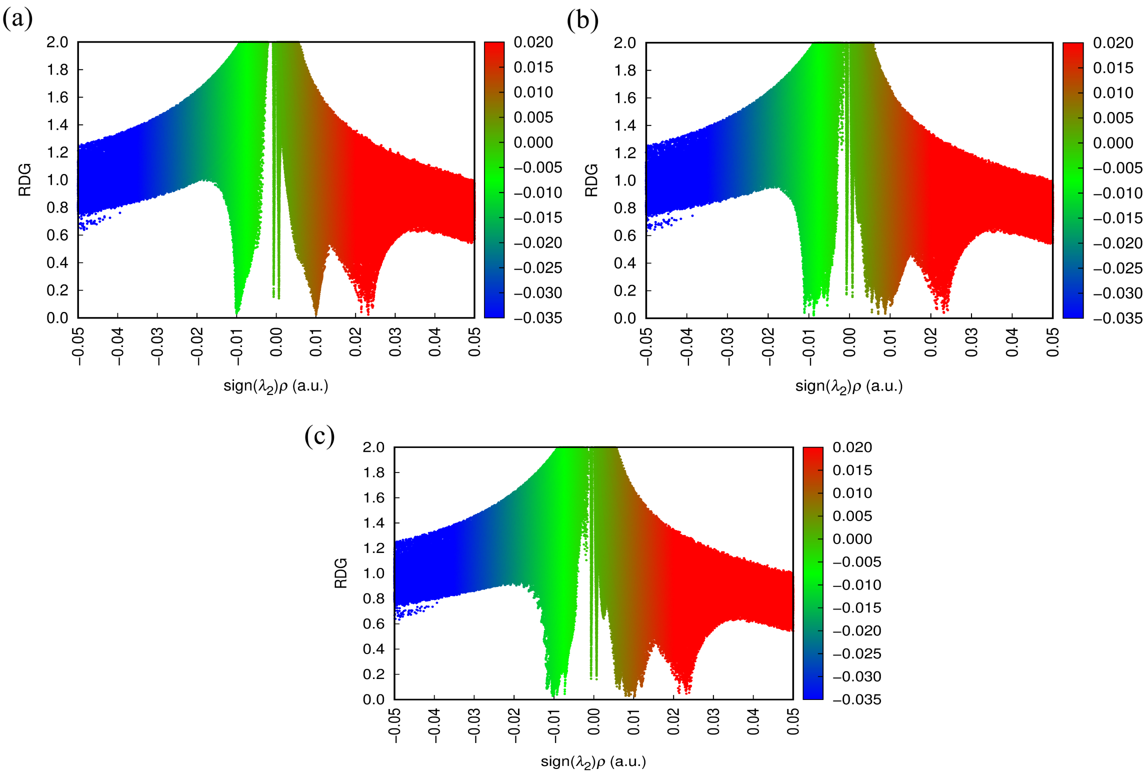

Figure S43. Colored RDG plots for (a) Bdan **1**, (b) *Z*-(*M*)-(*S*a), and (c) *Z*-(*M*)-(*R*a) isomers of Bdan **2** calculated at the M06-2X/Def2TZVP level of theory.

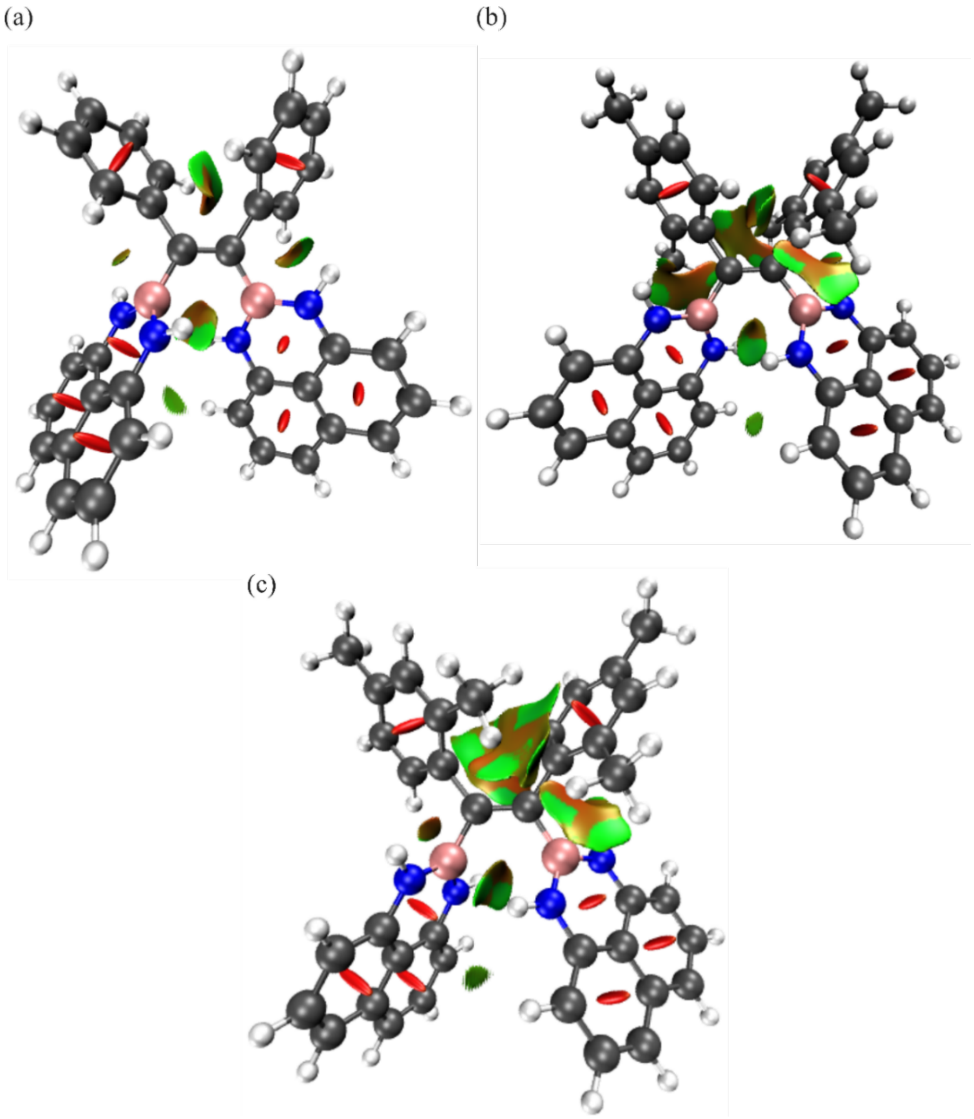

Figure S44. Visualization of regions of non-covalent interactions in Bdans **1** and **2** using the RDG-isosurface of 0.5. (a) Bdan **1**, (b) *Z*-(*M*)-(*S*a), and (c) *Z*-(*M*)-(*R*a) isomers of Bdan **2**.

9.5. Theoretical Studies on the Atropisomerization in Bdan 2

To shed light on the atropisomerization process of Bdan **2**, a PM6-DFT combined approach was utilized. Starting from *Z*-(*M*)-(*S*a)-**2** isomer, the PES exploration involving the torsion angle α associated with the 2,4-dimethyl phenyl ring and double bond was conducted at the PM6 level of theory (Figure S45). By scanning the α angle from 0º to 360º and taking 10º steps allowed for the identification of two possible TS structures (**TS-1** and **TS-2**). On the other hand, the PES exploration at the B3LYP/6-31G(d) level of theory involving the same torsion angle was also carried out by scanning from 0º to 180º and taking 10º steps to reduce computational cost and time. This strategy revealed **TS-3** and **TS-4** structures, where **TS-4** was deduced from **TS-3** and placing the methyl group

of the 2,4-dimethyl phenyl fragment orthogonal with respect to the mesitylene blade. Further geometry optimizations and frequency calculations were performed for all these relevant TS structures at the B3LYP/6-31G(d) level of theory.

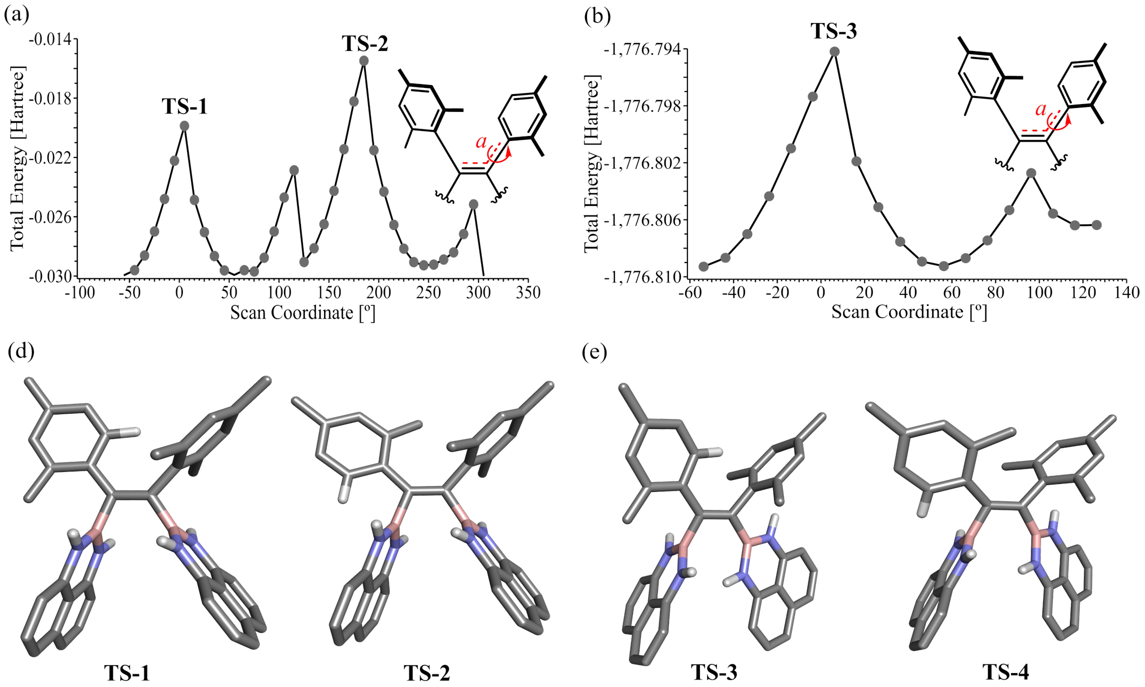

Figure S45. Results of the theoretical analysis of atropisomerization in Bdan **2** combining PM6 with DFT methods. Scan coordinate vs. total energy plots obtained at the (a) semi-empirical PM6 and (b) B3LYP/6-31G(d) level of theory when scanning torsion angle *a*. The PES exploration involved varying torsional angles from 0º to 360º at the PM6 level of theory, yet this was reduced to scanning from 0º to 180º for the B3LYP/6-31G(d) level of theory. In each method, independent scans were carried out starting from the *Z*-(*M*)-(*S*a)-**2** isomer and 5º steps were taken. Optimized transition state geometries (d) **TS-1** and **TS-2** and (e) **TS-3** and **TS-4** obtained at the B3LYP/6-31G(d) level of theory.

According to the theoretical analysis, the atropisomerization process of Bdan **2** involves correlated motions. For the optimized transition state geometries **TS-1** and **TS-2**, only the desymmetrized 2,4-dimethyl phenyl blade is encountered within the plane containing the central double bond. The remaining NBN-doped phenyl and mesitylene blades are placed nearly perpendicular to the reference double-bond plane. These transition states thus resemble the *three ring-flip mechanism* associated with the correlated rotation of vinyl propellers for helicity reversal.[21] [22] [23]The relative Gibbs free energies were computed as 13.69 kcal/mol for **TS-1** and 18.47 kcal/mol for **TS-2** at the B3LYP/6-31G(d) level of theory (Table S7). The destabilization of **TS-2**, compared to **TS-1**, can be justified by the *syn* arrangement of Me group in the desymmetrized phenyl blade with respect to the mesitylene unit. The optimized geometries for **TS-3** and **TS-4**, on the other hand, are

rather comparable with the *trans two-ring flip mechanism* of vinyl propellers. This means that both the NBN-doped phenyl and mesitylene rotate toward the same direction and perpendicular to the double-bond reference plane in *trans* fashion. The ΔG_{rel}^{0} of **TS-3** was calculated as 12.72 kcal/mol and this value was found to be 17.02 kcal/mol for **TS-4**. While the higher energy in **TS-4** can be again explained by the *syn* arrangement of *ortho* Me and mesitylene substituents, **TS-3** should constitute the atropisomerization threshold mechanism with the lowest activation energy of 10.70 kcal/mol. These results corroborate well with $\Delta G^{\neq}$ of 10.58 kcal/mol obtained experimentally from VT ^{1}H NMR experiments on Bdan **2** (for more details, see Section 8 in the Supporting Information). Figure S46 depicts theoretical descriptions of different atropisomerization mechanisms calculated at the B3LYP/6-31G(d) level of theory. Note that the *four-ring flip mechanism* is not considered due to a highly unfavorable proximity of all blades encountered in the reference double-bond plane.

Table S7. Ground-state energy profile of Bdan **2** involving minima conformers and transition states computed at the B3LYP/6-31G(d) level of theory. The relative Gibbs free energies (ΔG_{rel}^{0}) are given in kcal/mol with respect to the global minimum *Z*-(*M*)-(*S*a)-**2.**

***Z*-(*M*)-(*S*a)-2**	***Z*-(*M*)-(*R*a)-2**	**TS-1** ($\Delta G^{\neq}$)	**TS-2** ($\Delta G^{\neq}$)	**TS-3** ($\Delta G^{\neq}$)	**TS-4** ($\Delta G^{\neq}$)
0.00	2.02	13.69 (+11.67)	18.47 (+16.45)	12.72 (+10.70)	17.04 (+15.02)

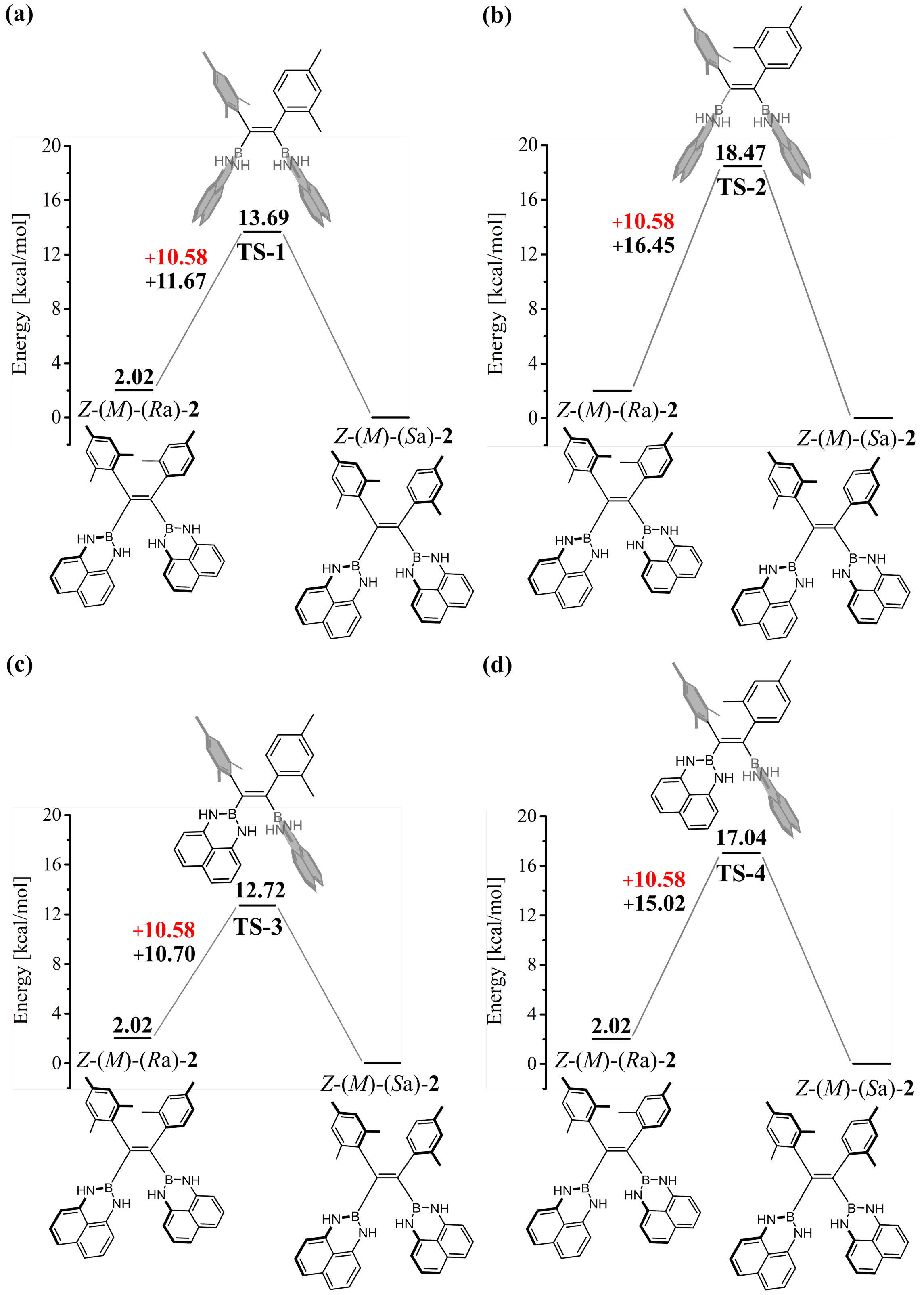

Figure S46. Theoretical descriptions of different atropisomerization mechanisms of Bdan **2** including (a) **TS-1**, (b) **TS-2**, (c) **TS-3** and (d) **TS-4** calculated at the B3LYP/6-31G(d) level of theory. Theoretically computed values are depicted in black, whereas the experimentally obtained Gibbs energies of activation for the atropisomerization process are shown in red.

10. NMR Spectra of Synthesized Compounds

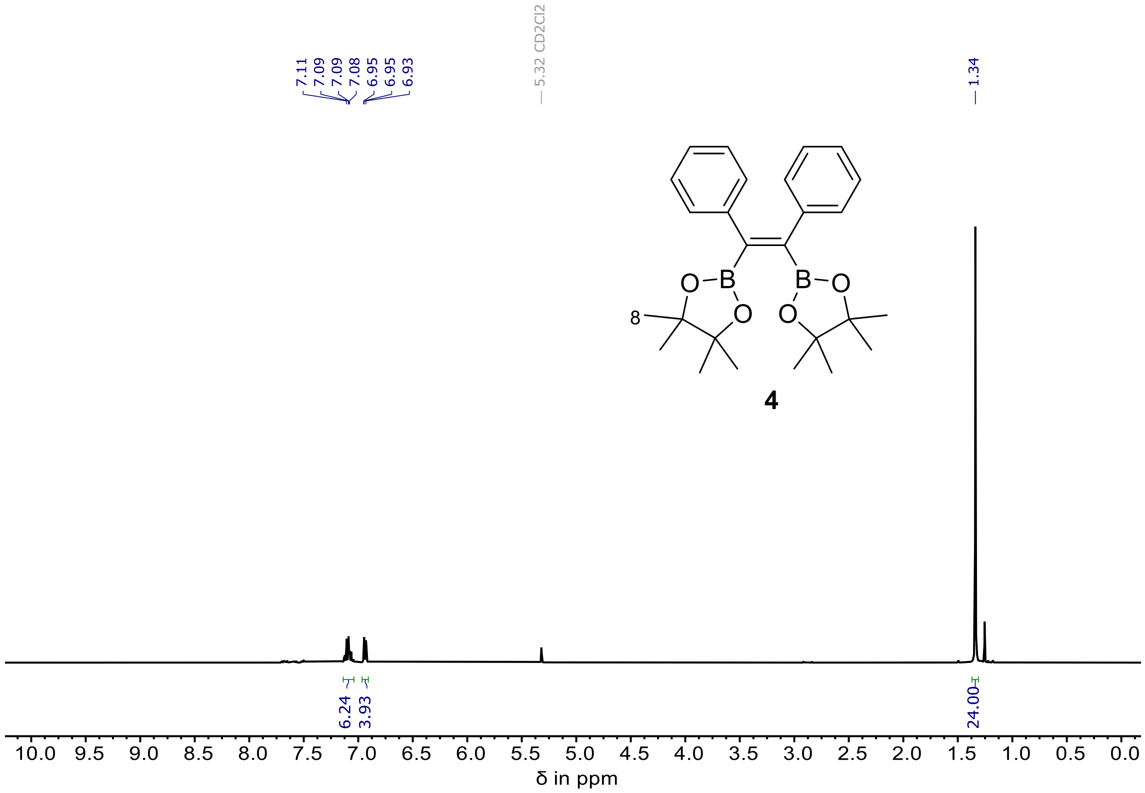

Figure S47. ^{1}H NMR spectrum (400 MHz, CD_2Cl_2, 23 °C) of compound **4**.

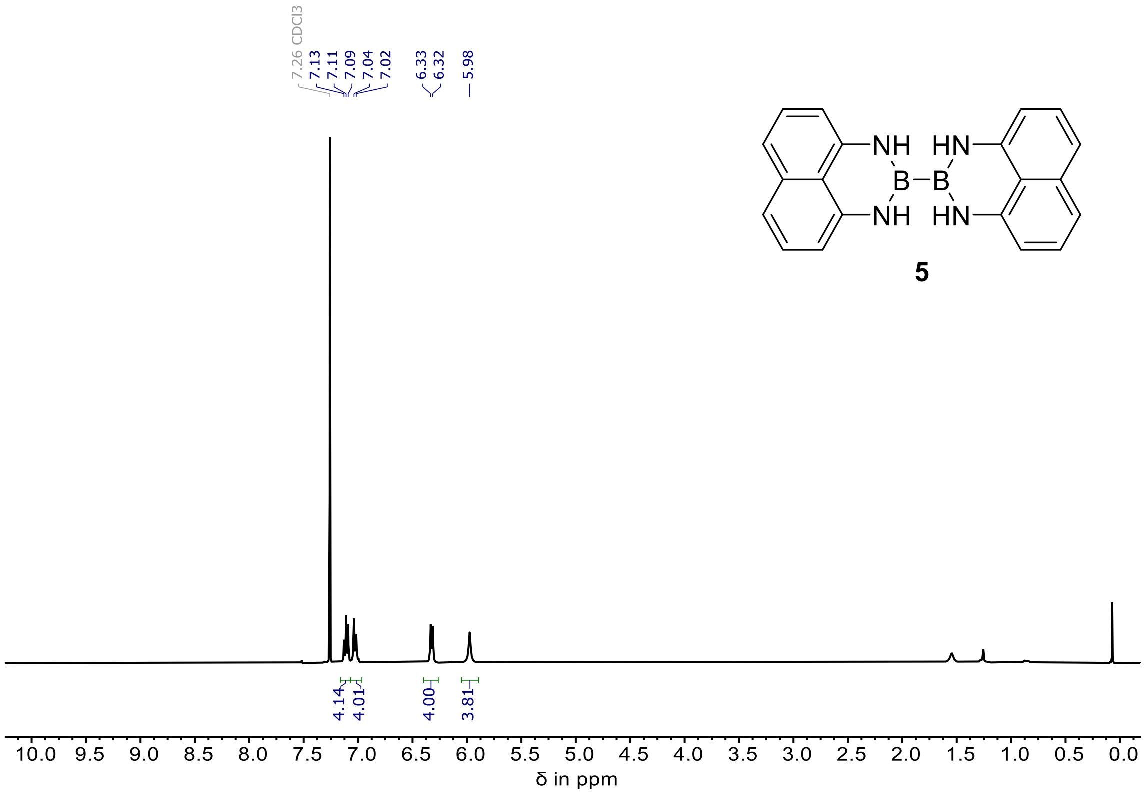

Figure S48. ^{1}H NMR spectrum (400 MHz, $CDCl_3$, 23 °C) of compound **5**.

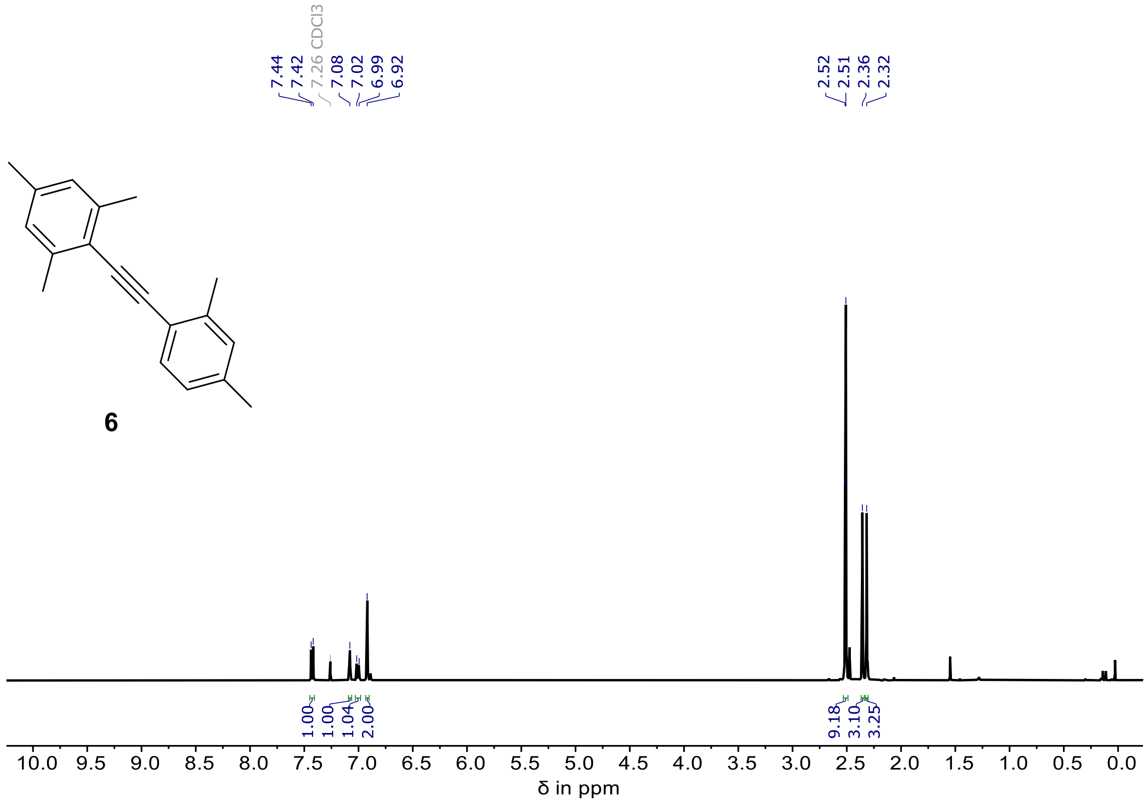

Figure S49. ^{1}H NMR spectrum (400 MHz, $CDCl_3$, 23 °C) of compound **6**.

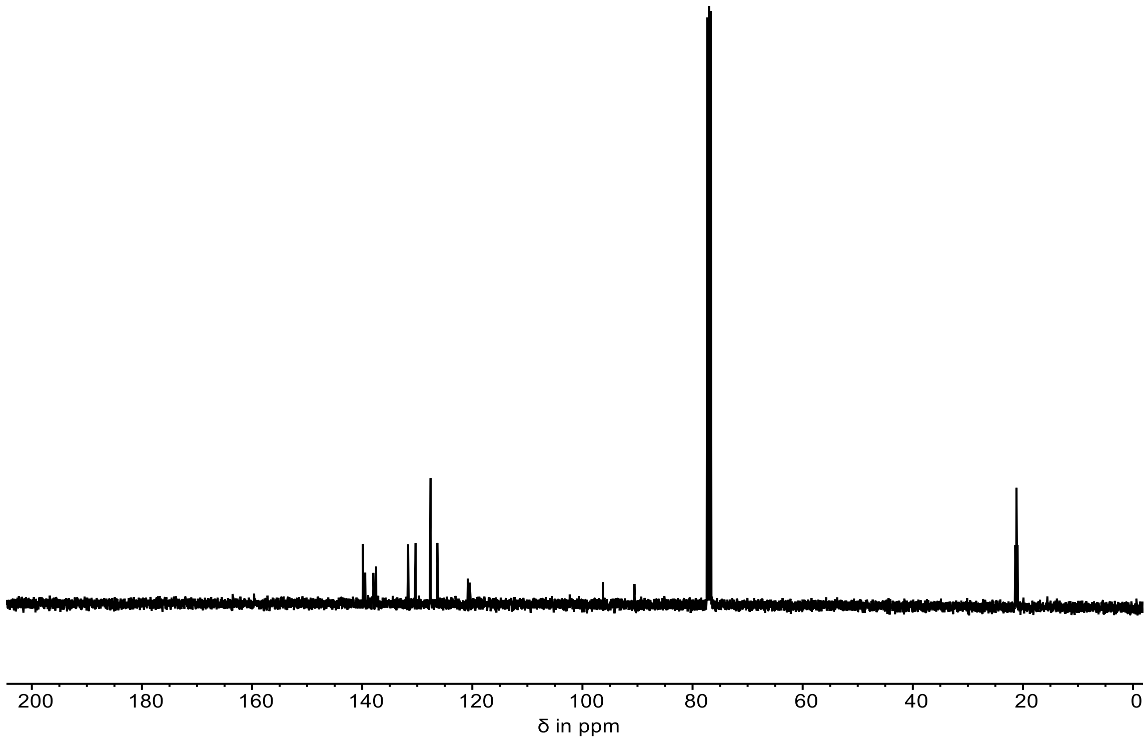

Figure S50. ^{13}C NMR spectrum (100 MHz, $CDCl_3$, 23 °C) of compound **6**.

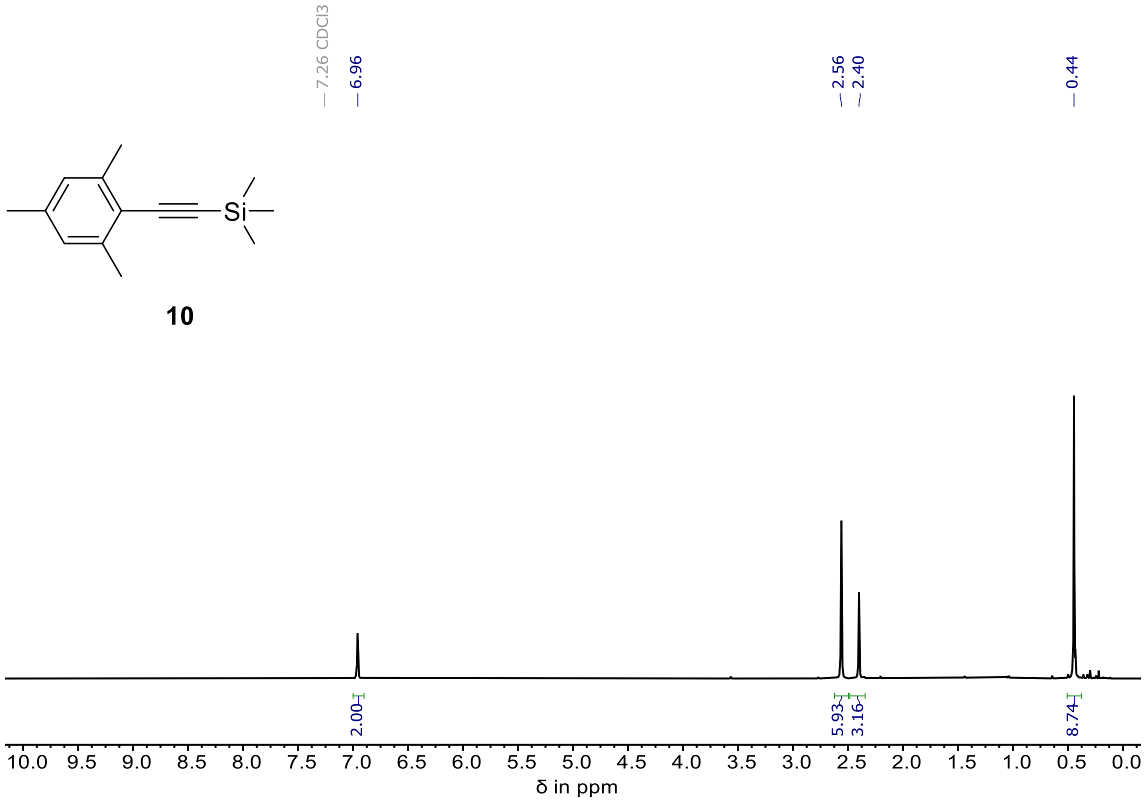

Figure S51. ^{1}H NMR spectrum (400 MHz, $CDCl_3$, 23 °C) of compound **10**.

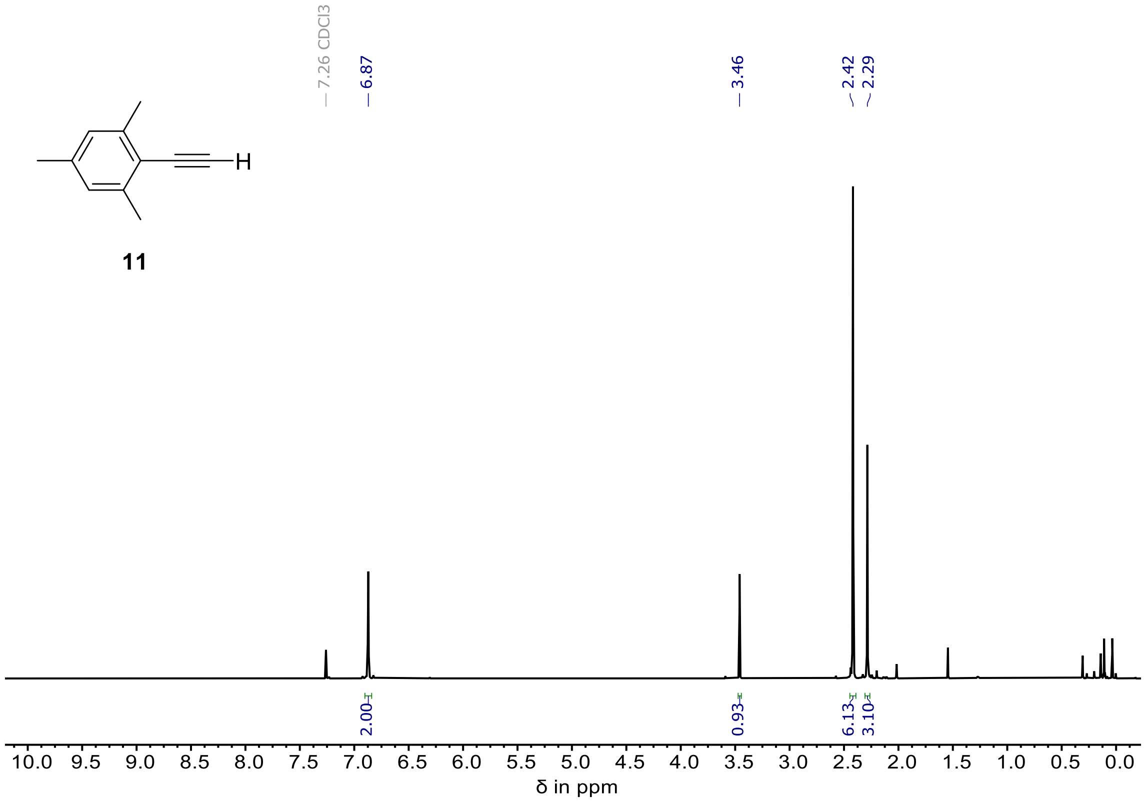

Figure S52. ^{1}H NMR spectrum (400 MHz, $CDCl_3$, 23 °C) of compound **11**.

11. Cartesian Coordinates of Optimized Structures

Bdan **1**			
N	0.40880300	-2.36926900	-0.88217900
C	1.69317300	-2.90937700	-0.82630200
C	-2.67460800	-1.96299000	-0.02046300
C	-1.59053500	-0.94313400	0.01662500
C	2.49844700	-2.59861600	0.30699400
C	4.10733700	-1.99941900	2.50824000
C	2.00009200	-1.78023900	1.36131800
C	-3.71298200	-1.89477800	-0.95246800
C	4.60973500	-2.79223300	1.51443400
C	3.81640700	-3.11334400	0.38667600
C	2.19213400	-3.70830600	-1.82765400
C	2.79966300	-1.48921200	2.44112200
C	4.29689400	-3.93199400	-0.66488600
C	-3.61994200	-4.03128400	0.81377300
C	3.49946600	-4.21827600	-1.73700200
C	-4.69008200	-2.87608700	-0.99737700
C	-4.65272800	-3.94341500	-0.10875100
C	-2.63341900	-3.05649900	0.84711300
B	-0.13619700	-1.54124900	0.13572500
H	2.41737200	-0.86148900	3.23644900
H	4.72084800	-1.75502500	3.36599900
H	5.61722500	-3.18332700	1.57282900
H	5.30337200	-4.32505700	-0.60188300
H	3.87305100	-4.84621700	-2.53581500
H	1.57323100	-3.94148500	-2.68535300
H	-1.82215700	-3.13707300	1.56279400
H	-3.57681800	-4.86377000	1.50412800
H	-5.41913000	-4.70670000	-0.14228300
H	-5.48487100	-2.80775500	-1.72903600
H	-3.74936000	-1.06256200	-1.64413300
H	0.39148600	-0.69010100	1.99967800
H	-0.14693700	-2.63819200	-1.67953800
N	0.58429100	1.14081300	-0.89729600

N	-0.67657900	2.68416200	0.45212800
C	0.35167500	3.62099600	0.41009100
C	-3.16122100	0.99958500	-0.01545600
C	-1.80242800	0.38974400	-0.06386300
C	1.54264400	3.27399900	-0.29054500
C	3.89424300	2.61259200	-1.64121800
C	1.66712900	2.01522600	-0.94491500
C	-4.07617400	0.65230000	0.98035900
C	3.79875600	3.83268100	-1.03133400
C	2.61825200	4.19513500	-0.33872300
C	0.24869800	4.84676500	1.02536700
C	2.83088900	1.69490800	-1.60438300
C	2.47624100	5.44715200	0.30881900
C	-4.78622000	2.56296400	-0.89902400
C	1.31933500	5.75618900	0.96774100
C	-5.32718300	1.24673500	1.03357400
C	-5.69002900	2.19878400	0.08934800
C	-3.52911400	1.97702100	-0.94192700
B	-0.60594000	1.41110900	-0.17240300
H	2.92364700	0.73002300	-2.08813000
H	4.80219800	2.33985600	-2.16390900
H	4.62092500	4.53578600	-1.06584000
H	3.29812500	6.15045000	0.27154400
H	1.21796200	6.71551000	1.45927900
H	-0.65965100	5.10480000	1.55571000
H	-2.82273800	2.27326100	-1.70943900
H	-5.05732100	3.30847100	-1.63545100
H	-6.66839500	2.65958100	0.12887200
H	-6.02204700	0.96524400	1.81433200
H	-3.79917000	-0.09598500	1.71271800
H	0.70707000	0.27624200	-1.40304700
H	-1.50795200	2.96386000	0.95057900

Bdan **2** (*Z*-(*M*)-(*S*a))			
N	0.35375600	2.82405700	0.49151900
N	-1.20269800	1.49640300	-0.77087200
C	-2.12231500	2.54190000	-0.79957200
C	2.53152000	0.93700700	-0.05806200
C	1.12570000	0.41755900	-0.05001500
C	-1.76417100	3.76925100	-0.17196400
C	-1.07969100	6.19005800	1.03739400
C	-0.50558900	3.91770700	0.47848500
C	3.34034500	0.86363500	1.08153000
C	-2.29666300	6.07381600	0.42555400
C	-2.67131500	4.85754800	-0.19626600
C	-3.34750900	2.41541400	-1.41230200
C	-0.17522200	5.11455900	1.07067500
C	-3.92135500	4.69164500	-0.84053800
C	4.30696000	2.08500200	-1.22064200
C	-4.24234700	3.49896700	-1.42700800
C	4.62606900	1.40300400	1.03760100
C	5.13260600	2.00550600	-0.10237400
C	3.01642400	1.57848000	-1.21157100
B	0.05222800	1.57336800	-0.11278300
H	0.78380000	5.22129200	1.56229100
H	-0.80012900	7.12351300	1.50928100
H	-2.98939000	6.90524200	0.40625700
H	-4.61463700	5.52264400	-0.85791000
H	-5.20136000	3.37794200	-1.91452600
H	-3.61826700	1.47631900	-1.87913300
H	4.67894500	2.56172400	-2.12178500
H	5.24543200	1.34584700	1.92648900
H	1.25244000	2.97171000	0.92754700
H	-1.50163300	0.65004600	-1.22967700
N	-0.91179700	-2.65639800	-0.70585600
N	-1.65397000	-1.01994600	0.89185900
C	-2.93233000	-1.57200700	0.91577800
C	1.93268800	-1.93393700	-0.02550700
C	0.85105400	-0.90328900	0.02309900

C	-3.18027300	-2.72333600	0.11421100
C	-3.68995400	-4.98816600	-1.43634100
C	-2.15554800	-3.28088100	-0.70365500
C	2.84833200	-1.93565900	-1.07705100
C	-4.69408200	-4.46993000	-0.66716400
C	-4.46505300	-3.32113000	0.12909900
C	-3.94098900	-1.03965700	1.68459000
C	-2.41415300	-4.39827900	-1.46284500
C	-5.47678900	-2.74833500	0.93744200
C	3.01927300	-3.90798400	0.82566000
C	-5.21344900	-1.63639400	1.68806800
C	3.83094600	-2.90723700	-1.17972600
C	3.93582900	-3.90840400	-0.22134600
C	2.01560400	-2.95223900	0.93948100
B	-0.60215600	-1.51273800	0.07835400
H	-1.63052100	-4.81765000	-2.08161000
H	-3.87261400	-5.86710500	-2.04148200
H	-5.67520600	-4.92676000	-0.65464800
H	-6.45835000	-3.20428800	0.94715400
H	-5.99320100	-1.20112200	2.30001600
H	-3.74896100	-0.15833300	2.28396600
H	3.08784400	-4.68193600	1.58353300
H	4.52435900	-2.88435300	-2.01210800
H	2.78225700	-1.16087000	-1.83059200
H	-0.20024500	-3.08904300	-1.27608900
H	-1.52391800	-0.20124400	1.46591700
C	1.05527600	-3.01890000	2.09798900
H	0.84495100	-2.02765800	2.50244500
H	0.09958000	-3.45604700	1.79594400
H	1.46224000	-3.63572200	2.89827500
C	5.02263400	-4.94475700	-0.29219700
H	4.67968900	-5.90391100	0.09566400
H	5.36245400	-5.08940600	-1.31723500
H	5.88609500	-4.63860900	0.30269700
C	2.86595000	0.21550900	2.35406800
H	2.98312100	-0.86924600	2.29948700

H	3.44712800	0.57211900	3.20369600
H	1.81142600	0.41973300	2.54363300
C	2.15032300	1.71178100	-2.43573200
H	1.34919800	2.43881900	-2.27405000
H	2.73618900	2.04862900	-3.28956200
H	1.67077600	0.76567800	-2.69513800
C	6.53176800	2.55461400	-0.14411800
H	6.54461400	3.56663300	-0.55102100
H	6.97526900	2.58005200	0.85035700
H	7.17019900	1.93875600	-0.78087500

Bdan **2** (*Z*-(*M*)-(*R*a))			
N	-0.09678800	2.62854000	0.60992000
N	-1.26897500	1.11514000	-0.85063700
C	-2.40661300	1.91758700	-0.82185700
C	2.49128700	1.22355600	-0.06981500
C	1.19136300	0.48849900	-0.09369900
C	-2.35624700	3.12858200	-0.07341900
C	-2.27984100	5.51703100	1.37096900
C	-1.18299100	3.49745900	0.64616200
C	3.40763600	1.09363600	0.98252400
C	-3.42117900	5.18479000	0.69642400
C	-3.48946000	3.97904400	-0.04431300
C	-3.55568200	1.57348900	-1.49534500
C	-1.15272000	4.67671900	1.35336500
C	-4.65212500	3.59458800	-0.75476700
C	3.97750600	2.81442600	-1.12621000
C	-4.67610500	2.42032300	-1.45493600
C	4.60144400	1.81753100	0.93978800
C	4.91260900	2.67071900	-0.10367700
C	2.77790700	2.12255600	-1.12100400
B	-0.08698700	1.41061200	-0.12135900
H	-0.25736500	4.95148000	1.89722800
H	-2.23504000	6.44083000	1.93380200
H	-4.28687800	5.83415500	0.71746600
H	-5.51803500	4.24352000	-0.73017000

H	-5.57077000	2.12937800	-1.99061200
H	-3.59224600	0.64393700	-2.05048500
H	4.19408400	3.48500800	-1.95151600
H	5.30242100	1.70710600	1.76071000
H	0.72619300	2.92499800	1.11346700
H	-1.34316500	0.28256400	-1.41625000
N	-0.83350400	-2.36587400	-0.98858600
N	-1.20116600	-1.38368100	1.17932600
C	-2.47679100	-1.93868100	1.25323500
C	2.21115800	-1.82042400	0.04279900
C	1.07526100	-0.85710000	-0.02975000
C	-2.91743900	-2.75142600	0.16928800
C	-3.80375000	-4.36249300	-1.93356400
C	-2.08446800	-2.98091400	-0.96326200
C	2.15328700	-2.78139700	1.06388100
C	-4.62708300	-4.15458500	-0.86294700
C	-4.20491800	-3.34174100	0.21781700
C	-3.30392200	-1.72409600	2.33042100
C	-2.52701300	-3.77614300	-1.99379900
C	-5.02727200	-3.09915000	1.34426200
C	4.31347500	-2.79238800	-0.61128100
C	-4.58144700	-2.30824300	2.36630400
C	3.16581200	-3.69522400	1.26813800
C	4.28089900	-3.70547900	0.43038500
C	3.30332400	-1.85306900	-0.83387200
B	-0.34382200	-1.54173900	0.06056700
H	-1.88725000	-3.94774100	-2.85075000
H	-4.13268900	-4.98640000	-2.75488000
H	-5.61001200	-4.60597900	-0.82357700
H	-6.01158000	-3.54785800	1.37880700
H	-5.21734700	-2.12327000	3.22264900
H	-2.96634800	-1.09929200	3.14799100
H	5.15498700	-2.80984300	-1.29666400
H	3.09277500	-4.41145600	2.07834600
H	1.29534500	-2.78606200	1.72811200
H	-0.25588600	-2.57818000	-1.78736500

H	-0.94283500	-0.77580300	1.94126900
C	3.43231600	-0.95649100	-2.03398900
H	2.47172700	-0.53848200	-2.32840800
H	4.10661300	-0.12210300	-1.82968300
H	3.84004200	-1.51840500	-2.87507900
C	5.39278600	-4.69357900	0.64601500
H	5.01336800	-5.71667900	0.63165700
H	6.15736000	-4.60295900	-0.12415900
H	5.86846200	-4.53683300	1.61591200
C	3.16944900	0.22056700	2.18610500
H	3.67798000	-0.73971600	2.07554400
H	3.56312600	0.70519300	3.07997800
H	2.11177700	0.01217000	2.33919000
C	1.81402900	2.33174900	-2.26012300
H	0.97277600	2.96065600	-1.95645500
H	2.31104300	2.82558000	-3.09378100
H	1.39496500	1.39106400	-2.61943400
C	6.20632700	3.43566500	-0.13821500
H	6.02293300	4.51145300	-0.15644600
H	6.82111800	3.21060800	0.73207900
H	6.78055300	3.18875300	-1.03291300

**TS-1** for Bdan **2**			
N	0.91332300	1.42980400	1.14988500
N	0.70099100	1.75492100	-1.22363800
C	1.89212400	2.48540300	-1.27013900
C	-2.35560500	1.52868200	0.11627900
C	-1.27504100	0.47777600	-0.01771100
C	2.60555900	2.68315400	-0.04847600
C	4.02965900	3.07236300	2.33844100
C	2.11500000	2.14463200	1.18024600
C	-2.83576000	2.24020000	-1.00560200
C	4.52542400	3.60533300	1.16869400
C	3.82858800	3.42497700	-0.05644600
C	2.38601800	3.01313400	-2.45361200
C	2.82464900	2.34113000	2.35499400

C	4.30034200	3.95065900	-1.28969700
C	-3.76312200	2.91363400	1.53570900
C	3.59122100	3.74475600	-2.45265000
C	-3.76551600	3.27187300	-0.82527000
C	-4.23543300	3.63653400	0.43566600
C	-2.83832600	1.87609400	1.40262600
B	0.14794600	1.19663800	-0.03126300
H	2.44867800	1.92772800	3.28794800
H	4.57110400	3.21348200	3.27043000
H	5.45531600	4.16750100	1.16619400
H	5.22890000	4.51494000	-1.29917100
H	3.96046700	4.14985200	-3.39145300
H	1.84005100	2.86050600	-3.38190300
H	-4.13004600	3.16181100	2.53015500
H	-4.13521500	3.79979100	-1.70300500
H	0.63149200	1.02961300	2.03362400
H	0.23188900	1.64490000	-2.11127000
N	0.64366100	-1.97597700	1.14500600
N	0.80750000	-1.78872000	-1.25000300
C	2.09328000	-2.34190900	-1.24418800
C	-2.68379400	-1.67166700	-0.17808400
C	-1.40492700	-0.88572400	-0.10732300
C	2.64772500	-2.73225800	0.01388500
C	3.76054000	-3.51021300	2.47198000
C	1.91603500	-2.55030700	1.22816700
C	-3.92767700	-1.01116500	-0.30667800
C	4.49006900	-3.69468600	1.31830500
C	3.95519400	-3.31089300	0.05878500
C	2.82195500	-2.52246200	-2.40915500
C	2.47181900	-2.93905200	2.43724800
C	4.66988300	-3.47937000	-1.15780300
C	-3.97743300	-3.74383100	-0.22944500
C	4.11104500	-3.09096500	-2.35542200
C	-5.14297900	-1.67765000	-0.38012600
C	-5.19554100	-3.07278200	-0.33287900
C	-2.73356700	-3.09901900	-0.15623800

B	0.01599900	-1.59381100	-0.08001400
H	1.91006800	-2.80094600	3.35841500
H	4.17812700	-3.80672100	3.43078600
H	5.48332500	-4.13366200	1.35498800
H	5.66404000	-3.91672800	-1.12628800
H	4.66645600	-3.22167400	-3.28058100
H	2.39575300	-2.22304000	-3.36381200
H	-3.98721300	-4.83187800	-0.20925800
H	-6.05952900	-1.09992500	-0.48089300
H	-3.95003400	0.06535900	-0.35989100
H	0.15551100	-1.87769300	2.02540500
H	0.45678700	-1.52034400	-2.15968200
C	-1.53102200	-4.01703600	-0.06575200
H	-0.78619400	-3.81721000	-0.84182700
H	-1.01560300	-3.94502500	0.89686300
H	-1.84944800	-5.05684700	-0.18594900
C	-6.50673300	-3.81849900	-0.38330900
H	-7.02804300	-3.77302200	0.58237800
H	-7.18296000	-3.39008800	-1.13219800
H	-6.35873600	-4.87580100	-0.62607900
C	-2.41164100	1.90117400	-2.41920800
H	-2.02747800	0.87975300	-2.49498800
H	-3.25687100	1.99091700	-3.11026700
H	-1.63616800	2.58997900	-2.78115900
C	-2.39455600	1.12921900	2.64087200
H	-1.34754800	1.34536100	2.88897200
H	-2.99710500	1.42217500	3.50622700
H	-2.48704200	0.04546800	2.51499900
C	-5.21129900	4.77600900	0.61254200
H	-5.75987100	4.98123300	-0.31272200
H	-5.94309300	4.56017400	1.39917000
H	-4.69475800	5.70223400	0.89812500

**TS-2** for Bdan **2**			
N	-0.67629800	-2.00621300	-1.16516500

N	-0.90139600	-1.20174100	1.08908200
C	-2.12240900	-1.86412600	1.24790900
C	2.38963100	-1.54786900	0.04200200
C	1.31020100	-0.51903600	-0.18856800
C	-2.61666100	-2.62916000	0.14800900
C	-3.60948200	-4.13088300	-2.00485000
C	-1.88659400	-2.70108700	-1.07723100
C	2.76779600	-2.47797700	-0.94948900
C	-4.33439000	-4.07923500	-0.83456900
C	-3.85941800	-3.32619600	0.27320700
C	-2.84704500	-1.79960100	2.42819700
C	-2.38475600	-3.44482500	-2.13660100
C	-4.57147700	-3.23589800	1.49945400
C	3.85681400	-2.70383300	1.60313500
C	-4.07145400	-2.48835800	2.54331000
C	3.68145700	-3.49360200	-0.64196100
C	4.23469200	-3.63421900	0.62873500
C	2.95021800	-1.67460600	1.34051900
B	-0.11676400	-1.23170400	-0.10330300
H	-1.82695000	-3.49708700	-3.06898300
H	-3.98121600	-4.70839000	-2.84754700
H	-5.27796600	-4.61029300	-0.74297500
H	-5.51642400	-3.76322600	1.59823900
H	-4.62500600	-2.42273600	3.47654900
H	-2.46894500	-1.21302300	3.26209000
H	4.28056500	-2.78304700	2.60280900
H	3.97354700	-4.18844200	-1.42798700
H	-0.20335100	-2.09243100	-2.05241000
H	-0.61682700	-0.63543000	1.87597800
N	-0.62203100	2.10262600	1.00192000
N	-0.86343800	1.61384700	-1.34324300
C	-2.14641300	2.17220900	-1.36772400
C	2.55212300	1.80643600	-0.31844400
C	1.39312000	0.85079500	-0.19855500
C	-2.66363100	2.71441900	-0.15087000
C	-3.69938600	3.79690000	2.22429100

C	-1.89414600	2.68389400	1.05285200
C	2.23268200	3.15847700	-0.02987000
C	-4.46480900	3.83824400	1.07994400
C	-3.96889500	3.29954200	-0.13816300
C	-2.90814000	2.21491700	-2.52463600
C	-2.41184700	3.22174800	2.22095600
C	-4.71925700	3.32138600	-1.34462200
C	4.85592500	2.59997400	-0.48548100
C	-4.19513600	2.79012300	-2.50248500
C	3.16711700	4.18173600	0.04826100
C	4.52182100	3.91327600	-0.15310200
C	3.93218800	1.54872500	-0.58415500
B	-0.04310900	1.54377400	-0.18224000
H	-1.82108300	3.19839800	3.13402200
H	-4.08684800	4.21214300	3.15115900
H	-5.45648100	4.28215900	1.09229600
H	-5.71218600	3.76261600	-1.33730700
H	-4.77722300	2.81040000	-3.42022400
H	-2.51029100	1.80015700	-3.44776300
H	5.89747000	2.37418600	-0.70713000
H	2.83500100	5.19315000	0.27188400
H	1.19915100	3.42682500	0.15342700
H	-0.09668400	2.14052500	1.86579700
H	-0.53981200	1.23264900	-2.22216200
C	4.49455300	0.25163700	-1.11905000
H	5.44401900	0.44784500	-1.62812800
H	4.68675100	-0.48872100	-0.33859300
H	3.82002200	-0.20571500	-1.84456100
C	5.57334800	4.98830200	-0.02835700
H	5.20160200	5.95598200	-0.38315300
H	5.87931800	5.12426900	1.01791000
H	6.47332200	4.73926700	-0.60028900
C	2.27971600	-2.38874800	-2.38121600
H	1.79627700	-1.42993300	-2.59160900
H	3.11661900	-2.49086400	-3.08208300
H	1.57533800	-3.19705900	-2.61837900

C	2.59910200	-0.71937100	2.46087100
H	2.83712800	0.31831100	2.20536900
H	1.53002100	-0.75754600	2.70330900
H	3.14977500	-0.97732600	3.37076200
C	5.19992600	-4.75023400	0.95227900
H	4.73979200	-5.49695800	1.61257400
H	5.52829800	-5.26989100	0.04640300
H	6.09229600	-4.37400300	1.46667900

**TS-3** for Bdan **2**			
N	0.24515100	2.65804600	0.42760100
N	-1.26528700	1.13665100	-0.64959600
C	-2.28362700	2.09457400	-0.64919700
C	2.54416300	1.07900900	-0.10986800
C	1.25076100	0.29867000	-0.08742200
C	-2.00647400	3.37783500	-0.08955900
C	-1.46542600	5.90698400	0.99535100
C	-0.71874200	3.66607100	0.45564500
C	3.13891300	1.51705900	1.09701400
C	-2.71747600	5.65518600	0.47767800
C	-3.02292300	4.38388800	-0.08016700
C	-3.53820500	1.82700700	-1.17907700
C	-0.45869000	4.91997800	0.99019800
C	-4.29460100	4.07097300	-0.63093800
C	4.23107900	2.30710200	-1.34808500
C	-4.53573800	2.82238300	-1.16313000
C	4.26985100	2.34121300	1.04435300
C	4.83247700	2.75182500	-0.16705700
C	3.09616900	1.49081900	-1.34263800
B	0.03628100	1.34336900	-0.10135900
H	0.52349000	5.13676200	1.40356600
H	-1.24123300	6.88298000	1.41871100
H	-3.48632600	6.42311300	0.48790900
H	-5.07163500	4.83069400	-0.62492700
H	-5.51279800	2.59161700	-1.58028000
H	-3.74927200	0.84611600	-1.59804900

H	4.65336100	2.60911000	-2.30496200
H	4.72535300	2.66475600	1.97869400
H	1.15720900	2.92849600	0.77305900
H	-1.51989300	0.24701600	-1.05789900
N	-1.18055300	-1.84331400	-1.12410400
N	-1.03001400	-1.75899000	1.28074500
C	-2.35326600	-2.19338400	1.41828800
C	2.25006100	-2.08367500	-0.02936600
C	1.12751500	-1.07382600	-0.05660000
C	-3.08930000	-2.48526200	0.22776600
C	-4.55823900	-3.07058200	-2.09515000
C	-2.49936100	-2.31857700	-1.06321300
C	3.59045900	-1.67490100	0.16922800
C	-5.15431800	-3.23717700	-0.86507300
C	-4.43911700	-2.94829800	0.32890000
C	-2.95079000	-2.35322800	2.65812000
C	-3.22923700	-2.60921400	-2.20504800
C	-5.01470800	-3.10019000	1.61884600
C	3.16550400	-4.35208600	-0.13207800
C	-4.28321100	-2.80545300	2.74826700
C	4.66783100	-2.54859300	0.21145600
C	4.47832300	-3.92247000	0.05402700
C	2.05516700	-3.49455300	-0.17672300
B	-0.36918400	-1.58971100	0.03192000
H	-2.77355500	-2.48257600	-3.18445200
H	-5.11331700	-3.29416300	-3.00240300
H	-6.17933300	-3.58961900	-0.78991800
H	-6.04061200	-3.44897400	1.69828100
H	-4.73277700	-2.92161200	3.73093300
H	-2.38764200	-2.12819100	3.56060900
H	2.98556400	-5.41880100	-0.24870500
H	5.66656500	-2.14850200	0.37375000
H	3.80928900	-0.62945800	0.30015900
H	-0.77712100	-1.80483900	-2.05177800
H	-0.54572200	-1.55886300	2.14611400
C	0.72795100	-4.19572500	-0.39362700

H	-0.00715600	-3.97494800	0.38520500
H	0.26893100	-3.94278400	-1.35378100
H	0.88089200	-5.27885300	-0.38715800
C	5.63438900	-4.89194400	0.07341900
H	6.22072000	-4.83046100	-0.85310900
H	6.32143300	-4.67931300	0.90090100
H	5.29011000	-5.92600700	0.17667200
C	2.61152400	1.05449900	2.43907900
H	2.73247300	-0.03000500	2.55599200
H	3.15026500	1.54060700	3.25847400
H	1.54420400	1.27133700	2.55994500
C	2.48453800	1.04326700	-2.65068600
H	1.43584600	1.35522100	-2.73326400
H	3.02800800	1.46542200	-3.50165200
H	2.50128200	-0.04896700	-2.74629400
C	6.03511600	3.66605100	-0.20130000
H	6.69995300	3.42314600	-1.03761100
H	5.73582500	4.71598400	-0.32178400
H	6.61699300	3.59799200	0.72394500

**TS-4** for Bdan **2**			
N	-0.02153500	2.50148100	0.54711400
N	-1.18784800	1.01508300	-0.94119300
C	-2.32312200	1.83181100	-0.92331200
C	2.51296300	1.29060700	-0.15673100
C	1.31735100	0.37994000	-0.20639200
C	-2.27840400	3.03105200	-0.14847800
C	-2.20485900	5.39741100	1.35893600
C	-1.10854500	3.37555800	0.59666300
C	3.18220500	1.51028100	1.06842100
C	-3.34710700	5.08874300	0.65311100
C	-3.41609600	3.89755500	-0.11969200
C	-3.46829200	1.50967200	-1.63679000
C	-1.08022800	4.54718400	1.33881600
C	-4.56944700	3.53315200	-0.86507400
C	3.89090000	3.01111800	-1.17967100

C	-4.58631700	2.36675500	-1.59909800
C	4.19709800	2.47738100	1.13152500
C	4.57347800	3.23450600	0.02206300
C	2.86281500	2.07369100	-1.28286100
B	-0.00080000	1.28035200	-0.19548400
H	-0.18749200	4.80593600	1.90331400
H	-2.16165300	6.31123600	1.94633100
H	-4.20929300	5.74975900	0.67714000
H	-5.43673600	4.18760100	-0.84434700
H	-5.47523000	2.09418900	-2.16234800
H	-3.50228400	0.58934600	-2.21462600
H	4.16308500	3.58984600	-2.06080900
H	4.70836200	2.63605700	2.07929900
H	0.80459600	2.80601300	1.04687600
H	-1.28033500	0.16384700	-1.48049400
N	-0.90282400	-2.11789200	-1.04950500
N	-0.95848900	-1.51011700	1.28360900
C	-2.26963300	-1.98047600	1.41434900
C	2.30089300	-2.08212900	-0.16799700
C	1.27208700	-0.98420000	-0.03733900
C	-2.89555600	-2.55057700	0.26215800
C	-4.14518800	-3.68898100	-1.98205700
C	-2.20579200	-2.63082400	-0.98721100
C	1.79488700	-3.37015800	0.16045600
C	-4.83550300	-3.62277100	-0.79230900
C	-4.23139600	-3.05293200	0.36139900
C	-2.95856800	-1.91459600	2.61501600
C	-2.82861800	-3.19374900	-2.09026100
C	-4.90369800	-2.96484800	1.60982700
C	4.48260600	-3.19134400	-0.17433600
C	-4.27642300	-2.40744100	2.70236300
C	2.56919700	-4.51223300	0.29847100
C	3.95544500	-4.44011400	0.15294300
C	3.72152700	-2.02272200	-0.33418400
B	-0.21316300	-1.55043900	0.07252200
H	-2.29720600	-3.25497300	-3.03729200

H	-4.61501700	-4.12786200	-2.85849500
H	-5.85003200	-4.00475400	-0.71855100
H	-5.91960900	-3.34241900	1.68689100
H	-4.79936300	-2.34239100	3.65297300
H	-2.47919400	-1.47801900	3.48789100
H	5.55621100	-3.11394000	-0.33487700
H	2.08891400	-5.45763300	0.54073500
H	0.73200800	-3.49158700	0.33420200
H	-0.41699800	-2.27268600	-1.92404000
H	-0.55515000	-1.10948200	2.12033800
C	4.50131300	-0.83200300	-0.84838200
H	3.96086900	-0.30423600	-1.63515900
H	4.73575400	-0.09914500	-0.07243400
H	5.45090800	-1.17455600	-1.27220700
C	4.83815000	-5.64942700	0.33923800
H	4.94054600	-5.90523700	1.40231800
H	4.42234400	-6.53055100	-0.16313100
H	5.84445900	-5.47739800	-0.05573500
C	2.84739900	0.71926800	2.31591000
H	3.10868300	-0.33972900	2.20652400
H	3.39452900	1.11102000	3.17918800
H	1.77679200	0.75745100	2.54899500
C	2.15498300	1.88412600	-2.60578900
H	1.09175400	2.14628800	-2.53941600
H	2.60325000	2.51058100	-3.38312500
H	2.19986000	0.84128500	-2.94351400
C	5.67130200	4.26894100	0.10915500
H	6.12470700	4.28773200	1.10529900
H	6.46881400	4.06930500	-0.61728600
H	5.29099800	5.27629900	-0.10293400

12. References

[1] J. N. Nayak, M. I. Aralaguppi, B. V. Kumar Naidu, T. M. Aminabhavi, *Journal of Chemical & Engineering Data* **2004**, *49*, 468-474.

[2] M. Frisch, G. Trucks, H. Schlegel, G. Scuseria, M. Robb, J. Cheeseman, G. Scalmani, V. Barone, G. Petersson, H. Nakatsuji, *Wallingford CT* **2016**.

[3] M. Frisch, G. Trucks, H. Schlegel, G. Scuseria, M. Robb, J. Cheeseman, G. Scalmani, V. Barone, G. Petersson, H. Nakatsuji, Wallingford, CT, **2016**.

[4] T. Lu, F. Chen, *Journal of computational chemistry* **2012**, *33*, 580-592.

[5] P. K. Mandali, D. K. Chand, *Catalysis Communications* **2014**, *47*, 40-44.

[6] A. Grirrane, A. Corma, H. Garcia, *Chemistry–A European Journal* **2011**, *17*, 2467-2478.

[7] T. Yasuda, Y. Yoshigoe, S. Saito, *Organic Letters* **2023**, *25*, 2093-2097.

[8] S. Ito, M. Fukazawa, F. Takahashi, K. Nogi, H. Yorimitsu, *Bulletin of the Chemical Society of Japan* **2020**, *93*, 1171-1179.

[9] L. J. Bourhis, O. V. Dolomanov, R. J. Gildea, J. A. Howard, H. Puschmann, *Foundations of Crystallography* **2015**, *71*, 59-75.

[10] O. V. Dolomanov, L. J. Bourhis, R. J. Gildea, J. A. Howard, H. Puschmann, *Applied Crystallography* **2009**, *42*, 339-341.

[11] G. M. Sheldrick, *Foundations of crystallography* **2008**, *64*, 112-122.

[12] G. M. Sheldrick, *Crystal Structure Communications* **2015**, *71*, 3-8.

[13] I. Ino, L. P. Wu, M. Munakata, T. Kuroda-Sowa, M. Maekawa, Y. Suenaga, R. Sakai, *Inorganic Chemistry* **2000**, *39*, 5430-5436.

[14] D. F. Eaton, *Pure and Applied Chemistry* **1988**, *60*, 1107-1114.

[15] C. Würth, M. Grabolle, J. Pauli, M. Spieles, U. Resch-Genger, *Nature protocols* **2013**, *8*, 1535-1550.

[16] Y. Mutoh, K. Yamamoto, S. Saito, *ACS Catalysis* **2019**, *10*, 352-357.

[17] H. Gunther, in *NMR Spectroscopy*, Wiley, New York, NY, **1995**, pp. 335-390.

[18] M. T. Huggins, T. Kesharwani, J. Buttrick, C. Nicholson, *Journal of chemical education* **2020**, *97*, 1425-1429.

[19] F. P. Gasparro, N. H. Kolodny, *Journal of Chemical Education* **1977**, *54*, 258.

[20] H. Qu, X. Tang, X. Wang, Z. Li, Z. Huang, H. Zhang, Z. Tian, X. Cao, *Chemical Science* **2018**, *9*, 8814-8818.

[21] S. E. Biali, Z. Rappoport, *Journal of the American Chemical Society* **1984**, *106*, 477-496.

[22] E. Gur, Y. Kaida, Y. Okamoto, S. E. Biali, Z. Rappoport, *The Journal of Organic Chemistry* **1992**, *57*, 3689-3693.

[23] Z. Rappoport, S. E. Biali, *Accounts of chemical research* **1997**, *30*, 307-314.
